# Supplementary material for: Community control strategies for scabies: A cluster randomised noninferiority trial
Source: PLoS Med. 2021 Nov 10;18(11):e1003849. doi: 10.1371/journal.pmed.1003849 (PMC8612541; doi:10.1371/journal.pmed.1003849)
Supplement: S1 Table — (PDF) [file pmed.1003849.s001.pdf]

Supporting information file for *Community control strategies for scabies: a cluster randomised non-inferiority trial*

S1 Table. Medication dosing schedule by weight or age

Medication	Dose
**Ivermectin,** oral, 200 µg/kg	
< 15 kg	0 tablet
15–23 kg	1 tablet (3 mg)
24–38 kg	2 tablets (6 mg)
39–53 kg	3 tablets (9 mg)
54–68 kg	4 tablets (12 mg)
69–83 kg	5 tablets (15 mg)
84–98 kg	6 tablets (18 mg)
≥ 99 kg	7 tablets (21 mg)
**Permethrin 5%,** topical	
Age < 2 months	Apply for 4 hours
Age ≥ 2 months	Apply for 8 hours
**Diethylcarbamazine,** oral, 6mg/kg	
< 15 kg	0 tablet
15–25 kg	1 tablet (100 mg)
26–41 kg	2 tablets (200 mg)
42–58 kg	3 tablets (300 mg)
59–75 kg	4 tablets (400 mg)
76–92 kg	5 tablets (500 mg)
≥ 93 kg	6 tablets (600 mg)
**Albendazole,** oral	
< 15 kg	0 tablet
≥ 15 kg	1 tablet (400 mg)
