# Supplementary material for: Community control strategies for scabies: A cluster randomised noninferiority trial
Source: PLoS Med. 2021 Nov 10;18(11):e1003849. doi: 10.1371/journal.pmed.1003849 (PMC8612541; doi:10.1371/journal.pmed.1003849)
Supplement: S3 Table — IQR: interquartile range; IVM-1, one-dose ivermectin-based MDA; IVM-2, two-dose ivermectin-based MDA; MDA, mass drug administration; SAT, screen and treat with 1-dose permethrin to index cases of scabies and their household contacts. aVillages 1–17 are on Rotuma; Villages 18–35 are on Gau; median village size 125. (PDF) [file pmed.1003849.s003.pdf]

Supporting information file for *Community control strategies for scabies: a cluster randomised non-inferiority trial*

S3 Table. Population and participant demographics at 12-month follow-up

	Population demographics					Participant demographics					
	Total	Male		Age		Total		Male		Age	
**Village**[a]	N	n	%	Median	IQR	N	%	n	%	Median	IQR
IVM-2											
1	175	126	72.0	27	19-37	132	75.4	106	80.3	27	21-37.5
5	227	124	54.6	32	12-52	144	63.4	77	53.5	25.5	10.5-49
9	45	24	53.3	39	12-58	30	66.7	13	43.3	29.5	11-53
11	188	89	47.3	32.5	12-52	110	58.5	53	48.2	27.5	11-47
12	136	66	48.5	41.5	18.5-60.5	96	70.6	44	45.8	47.5	18-61
14	36	19	52.8	46	15-59	30	83.3	16	53.3	36.5	12-57
20	125	67	53.6	22	9-45	111	88.8	59	53.2	23	9-45
21	107	62	57.9	32	12-46	95	88.8	54	56.8	31	10-48
23	127	70	55.1	30	9-46	112	88.2	64	57.1	27.5	8.5-44.5
28	147	75	51.0	33	9-47	130	88.4	68	52.3	30.5	8-45
31	206	100	48.5	24	8-44	195	94.7	92	47.2	22	8-44
33	111	54	48.6	26	8-50	94	84.7	47	50.0	20	8-49
Total IVM-2	1630	876	53.7	30	11-49	1279	78.5	693	54.2	28	10-47
IVM-1											
6	80	43	53.8	42	12-56	63	78.8	33	52.4	46	11-56
7	69	36	52.2	28	13-45	51	73.9	26	51.0	24	11-41
10	272	157	57.7	32	13-51	233	85.7	139	59.7	28	12-48
13	66	28	42.4	46	14-64	52	78.8	24	46.2	32.5	13.5-53.5
15	156	73	46.8	32	13-53	109	69.9	50	45.9	25	12-52
17	98	52	53.1	18	9-45	89	90.8	47	52.8	16	9-40
19	97	58	59.8	24	10-44	79	81.4	43	54.4	21	8-44
22	125	64	51.2	31	9-51	119	95.2	59	49.6	31	9-51
24	48	28	58.3	39	27-53.5	38	79.2	20	52.6	41	28-54
25	94	44	46.8	30.5	9-51	82	87.2	38	46.3	21	9-47
27	165	86	52.1	24	10-45	126	76.4	64	50.8	23.5	10-45
35	173	82	47.4	17	15-19	155	89.6	74	47.7	16	15-19
Total IVM-1	1443	751	52.0	26	12-49	1196	82.9	617	51.6	23	11-47
SAT											
2	17	9	52.9	56	27-66	11	64.7	5	45.5	49	15-66
3	222	121	54.5	28.5	12-49	163	73.4	86	52.8	19	10-45
4	115	57	49.6	33	9-49	81	70.4	40	49.4	26	9-43
8	108	50	46.3	38	13.5-56	82	75.9	37	45.1	39	11-55
16	102	50	49.0	30.5	11-52	89	87.3	44	49.4	29	11-47
18	263	134	51.0	26	9-39	234	89.0	118	50.4	24.5	8-38
26	310	167	53.9	23	11-48	284	91.6	152	53.5	21.5	10.5-48.5
29	299	159	53.2	25	10-43	258	86.3	139	53.9	23	9-44
30	70	31	44.3	38	24-55	56	80.0	27	48.2	35.5	24-55
32	132	84	63.6	28	10.5-51	126	95.5	81	64.3	28	10-51
34	41	24	58.5	37	21-56	39	95.1	22	56.4	38	20-56
Total SAT	1679	886	52.8	29	11-49	1423	84.8	751	52.8	26	10-47
Total all	4752	2513	52.9	28	11-49	3898	82.0	2061	52.9	26	10-47

IVM-2: two-dose ivermectin-based mass drug administration; IVM-1: one-dose ivermectin-based mass drug administration; SAT: screen and treat with one-dose permethrin to index cases of scabies and their household contacts; IQR: interquartile range

[a] Villages 1–17 are on Rotuma; Villages 18–35 are on Gau; Median village size 125
