# Supplementary material for: Community control strategies for scabies: A cluster randomised noninferiority trial
Source: PLoS Med. 2021 Nov 10;18(11):e1003849. doi: 10.1371/journal.pmed.1003849 (PMC8612541; doi:10.1371/journal.pmed.1003849)
Supplement: S4 Table — CI, confidence interval; ICC, intracluster correlation; IVM-1, one-dose ivermectin-based MDA; IVM-2, two-dose ivermectin-based MDA; MDA, mass drug administration; SAT, screen and treat with 1-dose permethrin to index cases of scabies and their household contacts. aVillages 1–17 are on Rotuma; Villages 18–35 are on Gau. bOne-sided 97.5% CI. cAdjusted for clustering by village and stratified by island. dAdjusted for clustering by village. The ICC coefficient for scabies at baseline was 0.120, and 12 months was 0.207. (PDF) [file pmed.1003849.s004.pdf]

Supporting information file for *Community control strategies for scabies: a cluster randomised non-inferiority trial*

S4 Table. Scabies prevalence by village at baseline and 12 months

	Scabies prevalence								
	Baseline				12 months				Absolute reduction
**Village**[a]	N	n	%	(95% CI)	N	n	%	(95% CI)	%
IVM-2									
1	88	8	9.1	(4.0-17.1)	132	1	0.8	(0.0-4.1)	8.3
5	180	17	9.4	(5.6-14.7)	144	6	4.2	(1.5-8.8)	5.3
9	48	10	20.8	(10.5-35.0)	30	2	6.7	(0.8-22.1)	14.2
11	141	11	7.8	(4.0-13.5)	110	3	2.7	(0.6-7.8)	5.1
12	114	4	3.5	(1.0-8.7)	96	1	1.0	(0-5.7)	2.5
14	32	1	3.1	(0.1-16.2)	30	0	0.0	(0.0-11.6)[b]	3.1
20	90	14	15.6	(8.8-24.7)	111	2	1.8	(0.2-6.4)	13.8
21	78	18	23.1	(14.3-34.0)	95	0	0.0	(0.0-3.8)[b]	23.1
23	127	31	24.4	(17.2-32.8)	112	0	0.0	(0.0-3.2)[b]	24.4
28	146	8	5.5	(2.4-10.5)	130	0	0.0	(0.0-2.8)[b]	5.5
31	178	23	12.9	(8.4-18.8)	195	1	0.5	(0.0-2.8)	12.4
33	115	12	10.4	(5.5-17.5)	94	0	0.0	(0-3.8)[b]	10.4
Total IVM-2[c]	1337	157	11.7	(8.5-16.0)	1279	16	1.3	(0.6-2.5)	10.7[d]
IVM-1									
6	67	6	9.0	(3.4-18.5)	63	0	0.0	(0.0-5.7)[b]	9.0
7	47	8	17.0	(7.6-30.8)	51	1	2.0	(0.0-10.4)	15.1
10	204	12	5.9	(3.1-10.0)	233	5	2.1	(0.7-4.9)	3.7
13	59	1	1.7	(0.0-9.1)	52	0	0.0	(0-6.8)[b]	1.7
15	126	36	28.6	(20.9-37.3)	109	2	1.8	(0.2-6.5)	26.7
17	78	8	10.3	(4.5-19.2)	89	15	16.9	(9.8-26.3)	-6.6
19	84	16	19.0	(11.3-29.1)	79	0	0.0	(0.0-4.6)[b]	19.0
22	116	27	23.3	(15.9-32.0)	119	5	4.2	(1.4-9.5)	19.1
24	40	2	5.0	(0.6-16.9)	38	0	0.0	(0-9.3)[b]	5.0
25	75	3	4.0	(0.8-11.2)	82	1	1.2	(0.0-6.6)	2.8
27	127	11	8.7	(4.4-15.0)	126	3	2.4	(0.5-6.8)	6.3
35	159	50	31.4	(24.3-39.3)	155	0	0.0	(0.0-2.4)[b]	31.4
Total IVM-1[c]	1182	180	15.2	(9.4-23.8)	1196	32	2.7	(1.1-6.5)	11.1[d]
SAT									
2	18	2	11.1	(1.4-3.5)	11	0	0.0	(0.0-28.5)[b]	11.1
3	167	2	1.2	(0.1-4.2)	163	1	0.6	(0.0-3.4)	0.6
4	84	7	8.3	(3.4-16.4)	81	0	0.0	(0.0-4.5)[b]	8.3
8	78	5	6.4	(2.1-14.3)	82	4	4.9	(1.3-12.0)	1.5
16	86	5	5.8	(1.9-13.0)	89	0	0.0	(0.0-4.1)[b]	5.8
18	215	62	28.8	(22.9-35.4)	234	2	0.9	(0.1-3.1)	28.0
26	266	34	12.8	(9.0-17.4)	284	5	1.8	(0.6-4.1)	11.0
29	169	44	26.0	(19.6-33.3)	258	4	1.6	(0.4-3.9)	24.5
30	67	4	6.0	(1.7-14.6)	56	0	0.0	(0.0-6.4)[b]	6.0
32	106	9	8.5	(4.0-15.5)	126	0	0.0	(0.0-2.9)[b]	8.5
34	37	2	5.4	(0.7-18.2)	39	0	0.0	(0.0-9.0)[b]	5.4
Total SAT[c]	1293	176	13.6	(7.9-22.4)	1423	16	1.1	(0.6-2.0)	10.1[d]
Total All[c]	3812	513	13.5	(10.4-17.3)	3898	64	1.6	(1.0-2.7)	10.6[d]

IVM-2: two-dose ivermectin-based mass drug administration; IVM-1: one-dose ivermectin-based mass drug administration; SAT: screen and treat with one-dose permethrin to index cases of scabies and their household contacts

[a] Villages 1–17 are on Rotuma; Villages 18–35 are on Gau

[b] One-sided 97.5% CI

[c] Adjusted for clustering by village and stratified by island

[d] Adjusted for clustering by village

The intracluster correlation coefficient for scabies at baseline was 0.120 and 12 months was 0.207
