# Supplementary material for: Community control strategies for scabies: A cluster randomised noninferiority trial
Source: PLoS Med. 2021 Nov 10;18(11):e1003849. doi: 10.1371/journal.pmed.1003849 (PMC8612541; doi:10.1371/journal.pmed.1003849)
Supplement: S5 Table — CI, confidence interval; ICC, intracluster correlation; IVM-1, one-dose ivermectin-based MDA; IVM-2, two-dose ivermectin-based MDA; MDA, mass drug administration; SAT, screen and treat with 1-dose permethrin to index cases of scabies and their household contacts. aVillages 1–17 are on Rotuma; Villages 18–35 are on Gau. bOne-sided 97.5% CI. cAdjusted for clustering on village and stratified by island. dAdjusted for clustering on village. The ICC coefficient for impetigo at baseline was 0.07, and 12 months was 0.06. (PDF) [file pmed.1003849.s005.pdf]

Supporting information file for *Community control strategies for scabies: a cluster randomised non-inferiority trial*

S5 Table. Impetigo prevalence by village at baseline and 12 months

	Impetigo prevalence								
	Baseline				12 months				Absolute reduction
**Village[a]**	N	n	%	(95% CI)	N	n	%	(95% CI)	%
IVM-2									
1	88	6	6.8	(2.5-14.3)	132	1	0.8	(0-4.1)	6.1
5	180	1	0.6	(0-3.1)	144	5	3.5	(1.1-7.9)	-2.9
9	48	2	4.2	(0.5-14.3)	30	0	0.0	(0-11.6)[b]	4.2
11	141	5	3.5	(1.2-8.1)	110	0	0.0	(0-3.3)[b]	3.5
12	114	1	0.9	(0-4.8)	96	0	0.0	(0-3.8)[b]	0.9
14	32	1	3.1	(0.1-16.2)	30	1	3.3	(0.1-17.2)	-0.2
20	90	1	1.1	(0-6.0)	111	1	0.9	(0-4.9)	0.2
21	78	0	0.0	(0-4.6)[b]	95	0	0.0	(0-3.8)[b]	0.0
23	127	0	0.0	(0-2.9)[b]	112	1	0.9	(0-4.9)	-0.9
28	146	1	0.7	(0-3.8)	130	0	0.0	(0-2.8)[b]	0.7
31	178	5	2.8	(0.9-6.4)	195	2	1.0	(0.1-3.7)	1.8
33	115	2	1.7	(0.2-6.1)	94	2	2.1	(0.3-7.5)	-0.4
Total IVM-2[c]	1337	25	1.9	(1.1-3.3)	1279	13	1.0	(0.5-2.0)	1.1[d]
IVM-1									
6	67	1	1.5	(0-8.0)	63	0	0.0	(0-5.7)[b]	1.5
7	47	2	4.3	(0.5-14.5)	51	0	0.0	(0-7.0)[b]	4.3
10	204	2	1.0	(0.1-3.5)	233	3	1.3	(0.3-3.7)	-0.3
13	59	1	1.7	(0-9.1)	52	0	0.0	(0-6.8)[b]	1.7
15	126	3	2.4	(0.5-6.8)	109	1	0.9	(0-5.0)	1.5
17	78	2	2.6	(0.3-9)	89	4	4.5	(1.2-11.1)	-1.9
19	84	1	1.2	(0-6.5)	79	0	0.0	(0-4.6)[b]	1.2
22	116	6	5.2	(1.9-10.9)	119	0	0.0	(0-3.1)[b]	5.2
24	40	0	0.0	(0-8.8)[b]	38	0	0.0	(0-9.3)[b]	0.0
25	75	0	0.0	(0-4.8)[b]	82	0	0.0	(0-4.4)[b]	0.0
27	127	4	3.1	(0.9-7.9)	126	0	0.0	(0-2.9)[b]	3.1
35	159	5	3.1	(1-7.2)	155	1	0.6	(0-3.5)	2.5
Total IVM-1[c]	1182	27	2.3	(1.5-3.4)	1196	9	0.8	(0.3-1.8)	1.6[d]
SAT									
2	18	0	0.0	(0-18.5)[b]	11	0	0.0	(0-28.5)[b]	0.0
3	167	0	0.0	(0-2.2)[b]	163	0	0.0	(0-2.2)[b]	0.0
4	84	2	2.4	(0.3-8.3)	81	0	0.0	(0-4.5)[b]	2.4
8	78	0	0.0	(0-4.6)[b]	82	1	1.2	(0-6.6)	-1.2
16	86	3	3.5	(0.7-9.9)	89	0	0.0	(0-4.1)[b]	3.5
18	215	11	5.1	(2.6-9.0)	234	1	0.4	(0-2.4)	4.7
26	266	5	1.9	(0.6-4.3)	284	1	0.4	(0-1.9)	1.5
29	169	1	0.6	(0-3.3)	258	2	0.8	(0.1-2.8)	-0.2
30	67	3	4.5	(0.9-12.5)	56	0	0.0	(0-6.4)[b]	4.5
32	106	4	3.8	(1-9.4)	126	0	0.0	(0-2.9)[b]	3.8
34	37	1	2.7	(0.1-14.2)	39	0	0.0	(0-9.0)[b]	2.7
Total SAT[c]	1293	30	2.3	(1.3-4.1)	1423	5	0.4	(0.2-0.7)	2.0[d]
Total all[c]	3812	82	2.2	(1.6-2.9)	3898	27	0.7	(0.4-1.1)	1.5[d]

IVM-2: two-dose ivermectin-based mass drug administration; IVM-1: one-dose ivermectin-based mass drug administration; SAT: screen and treat with one-dose permethrin to index cases of scabies and their household contacts

[a] Villages 1–17 are on Rotuma; Villages 18–35 are on Gau

[b] One-sided 97.5% CI

[c] Adjusted for clustering on village and stratified by island

[d] Adjusted for clustering on village

The intracluster correlation coefficient for impetigo at baseline was 0.07 and 12 months was 0.06
