# Supplementary material for: Community control strategies for scabies: A cluster randomised noninferiority trial
Source: PLoS Med. 2021 Nov 10;18(11):e1003849. doi: 10.1371/journal.pmed.1003849 (PMC8612541; doi:10.1371/journal.pmed.1003849)
Supplement: S6 Table — IQR: interquartile range; IVM-1, one-dose ivermectin-based MDA; IVM-2, two-dose ivermectin-based MDA; MDA, mass drug administration; SAT, screen and treat with 1-dose permethrin to index cases of scabies and their household contacts. aParticipants allocated to treatment group of their current resident village in 2018. (PDF) [file pmed.1003849.s006.pdf]

Supporting information file for *Community control strategies for scabies: a cluster randomised non-inferiority trial*

S6 Table. Scabies prevalence at baseline and 12 months by treatment and demographic groups

	Treatment group																	
	IVM-2						IVM-1						SAT					
	Baseline			12 months[a]			Baseline			12 months[a]			Baseline			12 months[a]		
	N	n	%	N	n	%	N	n	%	N	n	%	N	n	%	N	n	%
**Sex**																		
Male	694	72	10.4	693	5	0.7	599	82	13.7	617	14	2.3	673	82	12.2	751	8	1.1
Female	643	85	13.2	586	11	1.9	583	98	16.8	579	18	3.1	620	94	15.2	672	8	1.2
**Age (years)**																		
Median (IQR)	8	(5-12)		6.5	(5-12.5)		12	(6.5-16)		7	(4-9.5)		9	(6-12.5)		5	(1.5-10.5)	
<2	35	8	22.9	30	1	3.3	20	6	30.0	38	2	5.3	38	11	28.9	44	4	9.1
2-4	94	25	26.6	73	2	2.7	74	22	29.7	64	8	12.5	80	13	16.3	87	3	3.4
5-9	210	62	29.5	202	7	3.5	149	45	30.2	143	14	9.8	182	71	39.0	212	4	1.9
10-14	201	37	18.4	166	3	1.8	158	42	26.6	156	4	2.6	172	49	28.5	193	4	2.1
15-24	121	10	8.3	110	1	0.9	207	38	18.4	214	2	0.9	134	12	9.0	149	1	0.7
25-34	140	4	2.9	166	0	0.0	124	8	6.5	121	0	0.0	154	5	3.2	181	0	0.0
35-49	257	8	3.1	238	1	0.4	192	5	2.6	197	2	1.0	236	4	1.7	245	0	0.0
50-64	188	2	1.1	195	1	0.5	170	9	5.3	170	0	0.0	207	8	3.9	218	0	0.0
≥65	91	1	1.1	99	0	0.0	88	5	5.7	93	0	0.0	90	3	3.3	94	0	0.0
**Island**																		
Rotuma	603	51	8.5	542	13	2.4	581	71	12.2	597	23	3.9	433	21	4.8	426	5	1.2
Gau	734	106	14.4	737	3	0.4	601	109	18.1	599	9	1.5	860	155	18.0	997	11	1.1
**Scabies total**	1337	157	11.7	1279	16	1.3	1182	180	15.2	1196	32	2.7	1293	176	13.6	1423	16	1.1

IVM-2: two-dose ivermectin-based mass drug administration; IVM-1: one-dose ivermectin-based mass drug administration; SAT: screen and treat with one-dose permethrin to index cases of scabies and their household contacts; IQR: interquartile range

[a] Participants allocated to treatment group of their current resident village in 2018
