# Supplementary material for: Community control strategies for scabies: A cluster randomised noninferiority trial
Source: PLoS Med. 2021 Nov 10;18(11):e1003849. doi: 10.1371/journal.pmed.1003849 (PMC8612541; doi:10.1371/journal.pmed.1003849)
Supplement: S7 Table — IQR: interquartile range; IVM-1, one-dose ivermectin-based MDA; IVM-2, two-dose ivermectin-based MDA; MDA, mass drug administration; SAT, screen and treat with 1-dose permethrin to index cases of scabies and their household contacts. aParticipants allocated to treatment group of their current resident village in 2018. (PDF) [file pmed.1003849.s007.pdf]

Supporting information file for *Community control strategies for scabies: a cluster randomised non-inferiority trial*

S7 Table. Impetigo prevalence at baseline and 12 months by treatment and demographic groups

	**Treatment group**																	
	**IVM-2**						**IVM-1**						**SAT**					
	Baseline			12 months[a]			Baseline			12 months[a]			Baseline			12 months[a]		
	N	n	%	N	n	%	N	n	%	N	n	%	N	n	%	N	n	%
**Sex**																		
Male	694	14	2.0	693	5	0.7	599	12	2.0	617	7	1.1	673	12	1.8	751	3	0.4
Female	643	11	1.7	586	8	1.4	583	15	2.6	579	2	0.3	620	18	2.9	672	2	0.3
**Age (years)**																		
Median (IQR)	6	(4-13)		5	(4-7)		9	(4-13)		7	(4-15)		7.5	(5-18)		9	(9-10)	
<2	35	3	8.6	30	2	6.7	20	2	10.0	38	2	5.3	38	5	13.2	44	0	0.0
2-4	94	4	4.3	73	3	4.1	74	6	8.1	64	1	1.6	80	1	1.3	87	1	1.1
5-9	210	10	4.8	202	6	3.0	149	7	4.7	143	2	1.4	182	12	6.6	212	2	0.9
10-14	201	2	1.0	166	1	0.6	158	7	4.4	156	1	0.6	172	4	2.3	193	1	0.5
15-24	121	1	0.8	110	1	0.9	207	4	1.9	214	2	0.9	134	2	1.5	149	0	0.0
25-34	140	0	0.0	166	0	0.0	124	0	0.0	121	0	0.0	154	3	1.9	181	0	0.0
35-49	257	1	0.4	238	0	0.0	192	0	0.0	197	1	0.5	236	1	0.4	245	0	0.0
50-64	188	4	2.1	195	0	0.0	170	0	0.0	170	0	0.0	207	2	1.0	218	1	0.5
≥65	91	0	0.0	99	0	0.0	88	1	1.1	93	0	0.0	90	0	0.0	94	0	0.0
**Island**																		
Rotuma	603	16	2.7	542	7	1.3	581	11	1.9	597	8	1.3	433	5	1.2	426	1	0.2
Gau	734	9	1.2	737	6	0.8	601	16	2.7	599	1	0.2	860	25	2.9	997	4	0.4
**Impetigo total**	1337	25	1.9	1279	13	1.0	1182	27	2.3	1196	9	0.8	1293	30	2.3	1423	5	0.4

IVM-2: two-dose ivermectin-based mass drug administration; IVM-1: one-dose ivermectin-based mass drug administration; SAT: screen and treat with one-dose permethrin to index cases of scabies and their household contacts; IQR: interquartile range

[a] Participants allocated to treatment group of their current resident village in 2018
