# Supplementary figures and images for: Community control strategies for scabies: A cluster randomised noninferiority trial
Source: PLoS Med. 2021 Nov 10;18(11):e1003849. doi: 10.1371/journal.pmed.1003849 (PMC8612541; doi:10.1371/journal.pmed.1003849)

S1 Fig. Randomisation and treatment flowchart

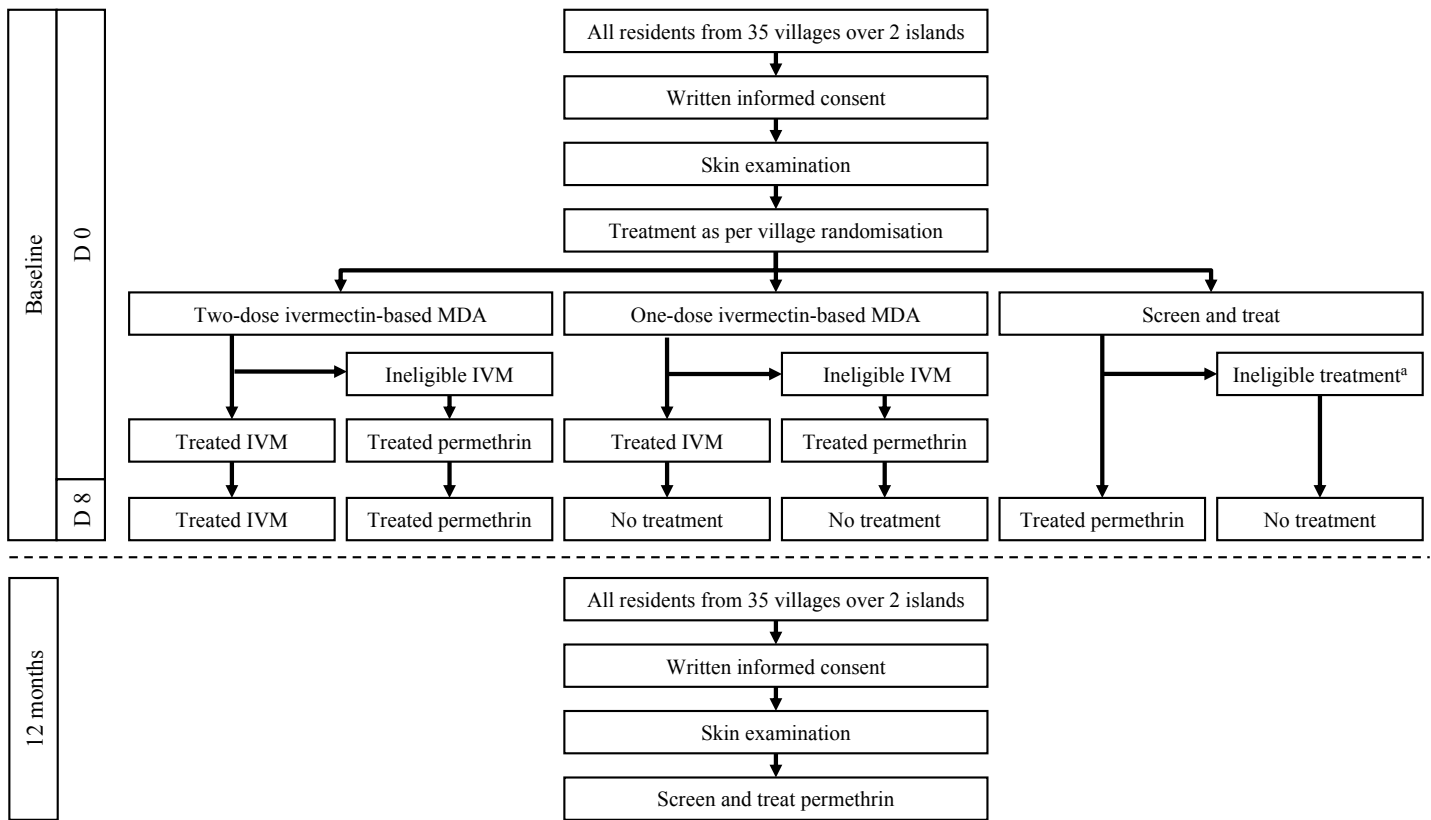

Supplement: S1 Fig — D, day; IVM, ivermectin; MDA, mass drug administration. aIneligible for treatment if not found to have scabies and no household contacts had scabies. (PDF) [file pmed.1003849.s008.pdf]
