# Supplementary material for: Community control strategies for scabies: A cluster randomised noninferiority trial
Source: PLoS Med. 2021 Nov 10;18(11):e1003849. doi: 10.1371/journal.pmed.1003849 (PMC8612541; doi:10.1371/journal.pmed.1003849)
Supplement: S1 Protocol — (PDF) [file pmed.1003849.s010.pdf]

Supporting information file for *Community control strategies for scabies: a cluster randomised non-inferiority trial*

S1 Protocol. Community Based Safety of 2-drug (Diethylcarbamazine and Albendazole) versus 3-drug (Ivermectin, Diethylcarbamazine and Albendazole) Therapy for Lymphatic Filariasis in Fiji – Protocol v6·0 6th August 2019

Sections relevant to scabies control include:
2.1 Fiji specific background, pages 14-15
3.3 Potential participant benefits, pages 16-17
4.1 Study objectives, page 18
4.2 Study design, pages 18-19
4.9 Eligibility, pages 22-27
4.10 Pregnant and breastfeeding females, pages 27-28
4.13 Efficacy and effectiveness of IDA vs DA, page28
4.14 Retreatment, page 28
5.1 Study drug background, page 30
6.1 Triple drug therapy (IDA) and two-drug therapy (DA), page 32
6.3 Skin examination, pages 33-34
9 Statistical considerations, page42
Appendix 2 – Participant data collection form example, pages 60-61
Appendix 6 – Participant information statement and consent form, pages 82-85
Appendix 6A – Parent information statement and consent form, pages 88-91
Appendix 7 – Treatment acceptability study protocol, pages 103-104

"Community Based Safety Study of 2-drug (Diethylcarbamazine and Albendazole) versus 3-drug (Ivermectin, Diethylcarbamazine and Albendazole) Therapy for Lymphatic Filariasis in Fiji"

Protocol Identifier: DOLF_IDA_Fiji

Type: Community Based Mass Drug Administration

Support Provided by: Bill and Melinda Gates Foundation

Project Principal Investigators:

Gary Weil, MD, Washington University, USA

Christopher King, MD, PhD, MPH, Case Western Reserve University, USA

Study Principal Investigators:

Professor Andrew Steer, MBBS, FRACP, PhD, Group A Streptococcus Group, Murdoch Children's Research Institute, Melbourne, Australia

Dr Josaia Samuela, MBBS, MPH, Eastern Division, Ministry of Health, Fiji

Study Co-investigators:

Dr Mike Kama, MBBS, National Director of Neglected Tropical Diseases, Ministry of Health, Fiji

Dr Meciusela Tuicakau, MBBS, PGDipDerm, Dermatologist, Twomey Hospital, Fiji

Dr Margot Whitfeld, MBBS, DTM&H, FACD, Sydney, Australia

Prof John Kaldor, PhD, Kirby Institute, Sydney, Australia

Dr Lucia Romani, MSocDev, PhD, Kirby Institute, Sydney, Australia

Dr Myra Hardy, MBBS, BMSc, FRACP, Group A Streptococcus Group, Murdoch Childrens Research Institute, Melbourne, Australia

Initial Protocol: v6.0 6th August 2019

INVESTIGATOR AGREEMENT

"Community Based Safety Study of 2-drug (Diethylcarbamazine and Albendazole) versus 3-drug (Ivermectin, Diethylcarbamazine and Albendazole) Therapy for Lymphatic Filariasis in Fiji"

DOLF_IDA_Fiji: v6.0 6th August 2019

I have read the protocol, including the appendices, and I agree that it contains all necessary details for me and my staff to conduct this study as described. I will conduct this study as outlined and make a reasonable effort to complete the study within the time designated.

I will provide all study personnel, participating in the study under my supervision copies of the protocol and access to all study related information provided by the DOLF project. I will discuss with them to ensure they are full informed about the study drug(s) and the study procedures.

Principle Investigator: **Dr Josaia Samuela**

Name/Title (Print/Type)

Signed: ______________________ **Date:** 12/8/2019

NOTE: Both the Project PI and local PI should have signed investigator agreements on file.

TABLE OF CONTENTS

TABLE OF CONTENTS 4
1 PROTOCOL SUMMARY 9
2 BACKGROUND INFORMATION AND RATIONALE 11
2.1 Fiji Specific Background 12
3 Potential Risks and Benefits 16
3.1 Risks of Blood Draw 16
3.2 Risks of Study Drugs 16
3.3 Potential Participant and Community Benefit 16
3.4 Subject Participation and Cost 17
3.5 Compensation for Injury 17
4 Study DESIGN AND OBJECTIVES 18
4.1 Study Objectives 18
4.1.1 Secondary Objectives 18
4.2 Study Design 18
4.3 Study Screening and Enrolment 20
4.4 Preparatory Activities 20
4.4.1 Social Mobilization 20
4.4.2 Household Enumeration and Census 20
4.5 Pre-Treatment Assessments 20
4.6 Informed Consent 21
4.7 Screening for Filarial Antigenemia and Microfilaria 22
4.8 Baseline Physical Examination and Pre-Treatment Adverse Event Monitoring 22
4.9 Eligibility 22
4.9.1 Inclusion Criteria for Treatment 22
4.9.2 Exclusion Criteria for Treatment 27
4.10 Pregnant and breastfeeding Females 27
4.11 Randomization – Community Assignment 28
4.12 Withdrawal 28
4.13 Efficacy and Effectiveness of IDA vs DA 28
4.14 Retreatment 28
4.15 Termination of the Study 28
4.16 Triple Drug Regimen Acceptability 29
5 INVESTIGATIONAL PRODUCT 30
5.1 Study Drug Background 30
5.1.1 Product Supply and Storage 31
6 Study Procedures/evaluations/Schedule 32
6.1 Triple Drug Therapy (IDA) and Two-Drug Therapy (DA) 32
6.2 Lymphatic Filariasis Testing 33
6.3 Skin Examination 33
6.4 Stool Collection 34
6.5 Clinic Records Review 35

6.6 Adverse Event Monitoring 35
6.7 Overall Study Schedule and Timeline 35
7 SAFETY REPORTING AND SAFETY MONITORING 37
7.1 Definitions 37
7.2 Assessment of Adverse Events 38
7.2.1 Serious Adverse Event (SAE) Assessment and Management 38
7.3 Reporting of Pregnancy 38
7.4 Safety Monitoring by the Oversight Committee 38
7.5 Country Specific Safety Reporting 39
8 Clinical Management of Events 40
8.1 Adverse Event Monitoring and Management 40
8.1.1 Mild Localized Symptoms 40
8.1.2 Moderate Adverse Events 40
8.1.3 Severe Adverse Events 40
8.2 Rapid Response Teams for Management of Adverse Events 40
9 STATISTICAL CONSIDERATIONS 42
9.1.1 Safety 42
9.1.2 Efficacy 42
10 DATA HANDLING/RECORD KEEPING/SOURCE DOCUMENTS 43
10.1 Types of Data Collected 43
10.2 Study Records Retention 43
10.3 Source Documents 44
11 Responsibilities 45
11.1 Investigator Responsibilities 45
11.1.1 Good Clinical Practice 45
11.2 Institutional Review Board (IRB)/Ethics Committee (EC) 45
11.3 Informed Consent 45
11.3.1 Informed Consent Training 45
11.4 Participant Privacy 46
11.5 Data Ownership 46
12 PUBLICATION POLICY 47
13 LITERATURE REFERENCES 52
14 List of Appendices 54
APPENDIX 1: Study Flow Diagram - Fiji 55
APPENDIX 2: Participant Enrollment Form [Example conversion to electronic database] 56
APPENDIX 3: Participant Monitoring Of Adverse Events Form [Example conversion to electronic database] 66
APPENDIX 4: Guide to Assigning Adverse Event Severity 72
APPENDIX 5: Serious Adverse Event (SAE) Form [Example] 75
APPENDIX 5A: Required Reporting & Guidelines for SAE(s) 78
APPENDIX 6: Participant Information Statement and Consent Form [Example] 81
APPENDIX 6A: Parent/Guardian Information Statement And Consent Form [Example] 87
APPENDIX 7: Treatment Acceptability Study Protocol 93

LIST OF ABBREVIATIONS

GENERAL PROJECT ABBREVIATIONS

AE	Adverse Event/Adverse Experience
AEERF	Adverse Event Evaluation and Report Form
Ag	Antigenemia
ALB	Albendazole
ARF	Acute Rheumatic Fever
CDD	Community Drug Distributor
CONSORT	Consolidated Standards of Reporting Trials
CRF	Case Report Form also referred to as eCRF (electronic case report form)
CRO	Contract Research Organization
DA	Two Drug Therapy (diethylcarbamazine and albendazole)
DCC	Data Coordinating Center
DEC	Diethylcarbamazine
DOLF	Death for Onchocerciasis and Lymphatic Filariasis
DOT	Directly Observed Treatment
DSMB, DSRB or DMC	Data and Safety Monitoring Board also called Data Safety Review Board or Data Monitoring Committee
EC	Ethics Committee (may also be called IRB or Institutional Review Board)
EDC	Electronic Data Capture
FDG	Focus Group Discussion
FIT	Fiji Integrated Therapy
FTS	Filariasis Test Strip
GCP	Good Clinical Practice

GPELF	Global Programme to Eliminate Lymphatic Filariasis
GPS	Global Positioning System
ICF	Informed Consent Form
ICH	International Conference on Harmonization
ICT	Immunochromatographic Test
IDA	Triple Drug Therapy (Ivermectin, Diethylcarbamazine,and Albendazole)
IMA	IMA World Health
IMCI	Integrated Management of Childhood Illnesses
IRB	Institutional Review Board (may also be called EC)
IVM	Ivermectin
LF	Lymphatic Filariasis
MDA	Mass Drug Administration
Mf	Microfilaria(e)
Mg	Milligram
NTD	Neglected Tropical Diseases
NLM	National Library of Medicine
PacELF	Pacific Programme to Eliminate Lymphatic Filariasis
PCR	Polymerase Chain Reaction
PI	Principal Investigator
PK	Pharmacokinetic(s)
PD	Pharmacodynamic(s)
QA	Quality Assurance
QC	Quality Control

RHD	Rheumatic Heart Disease
SAE	Serious Adverse Event/Serious Adverse Experience
SOP	Standard Operating Procedure
STH	Soil Transmitted Helminths
TAS	Transmission Assessment Surveys
TNT	Test and Treat
UNID	Unique Study Identification Numbers
USAID	United States Agency for International Development
WASH	Water And Sanitation and Hygiene

COUNTRY SPECIFIC ABBREVIATIONS

MCRI	Murdoch Childrens Research Institute
NHMRC	National Health and Medical Research Council
RCH	Royal Children's Hospital
SHIFT	Skin Health Intervention Fiji Trial
AIM	Azithromycin and Ivermectin Mass Drug Administration

1 PROTOCOL SUMMARY

**Study Title**	Community Based Safety Study of 2-drug (Diethylcarbamazine and Albendazole) versus 3-drug (Ivermectin, Diethylcarbamazine and Albendazole) Therapy for Lymphatic Filariasis in Fiji
**Type of Study**	Mass Drug Administration
**Population**	The whole population residing on the selected islands of Rotuma and Gau will be invited to participate.
**Number of Treated Areas**	Two islands from the Eastern Division, Rotuma and Gau.
**Duration of Subject Participation**	Approximately 1 year.
**Study Drugs**	Ivermectin (3 mg tablets) **not included in two drug treatment arm*<br>Diethylcarbamazine (100 mg tablets)<br>Albendazole (400 mg tablets)<br>Permethrin 5% cream
**Primary Objectives**	Determine the frequency, type, and severity of adverse events following triple drug therapy (IVM+DEC+ALB) compared to the standard two drug treatment (DEC+ALB) in infected and uninfected individuals in a community.
**Secondary Objectives**	Compare the efficacy of IDA (3 drug therapy) to DA (2 drug therapy) administered in communities for clearance of mf and filarial antigenemia (Ag).<br>Assess the effect of intensity of filarial infection on the frequency and severity of adverse events.<br>To evaluate the impact of IDA MDA on scabies and impetigo prevalence and the effect of 1 versus 2 doses of ivermectin.<br>To evaluate the impact of IDA MDA on STH (hookworm, ascaris, trichuris and strongyloides) prevalence.<br>To compare acceptability and feasibility of IDA MDA to DA MDA in communities at risk of LF, scabies and STH.
**DOLF PROJECT**	This protocol is specific to Fiji, but results will also be included in the larger DOLF project. Data will be available and reviewed at a country level and at the project level.

STUDY DESIGN

General Flow Diagram:

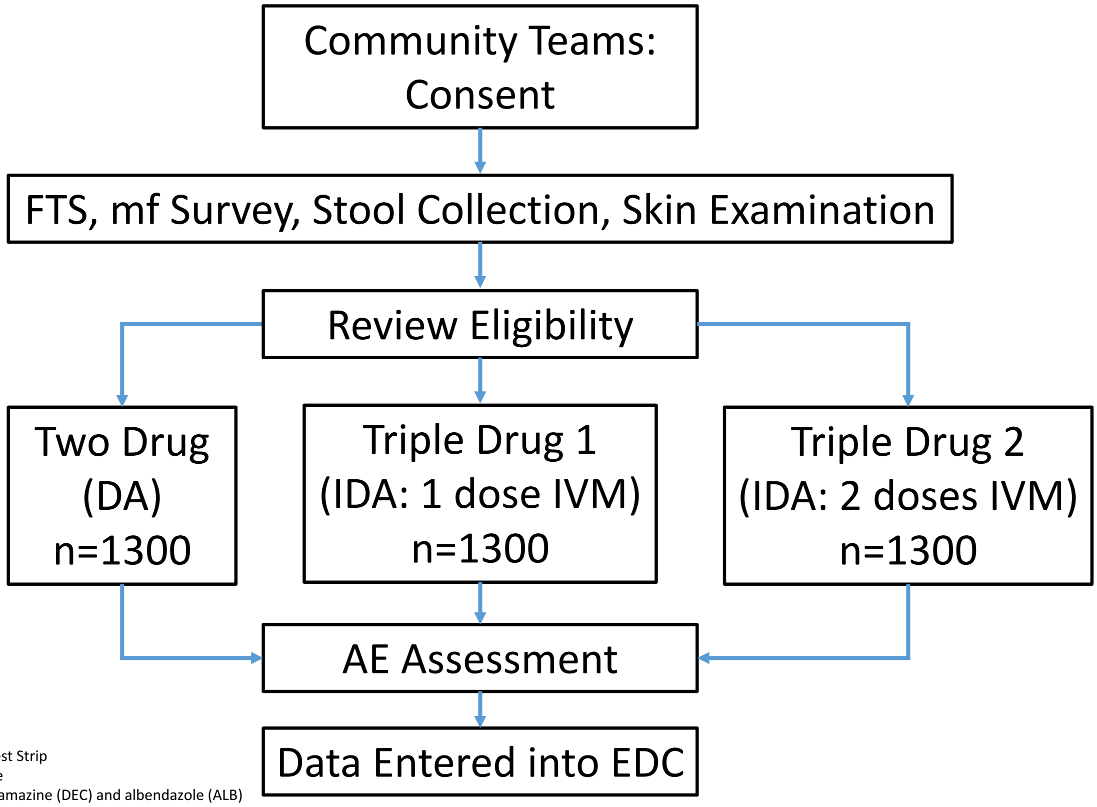

FTS: Filariasis Test Strip
mf: microfilariae
DA: Diethylcarbamazine (DEC) and albendazole (ALB)
IDA: Ivermectin (IVM), DEC and ALB
EDC: Electronic Data Capture

**DOLF_IDA_Fiji Study**	**ARM 1**	**Sample Size: 1300**	**Two Drug (DA)**
	**ARM 2**	**Sample Size: 1300**	**Triple Drug 1 (IDA: 1 dose IVM)**
	**ARM 3**	**Sample Size: 1300**	**Triple Drug 2 (IDA: 2 doses IVM)**

A Study Flow Diagram specific for Fiji is provided in Appendix 1.

2 BACKGROUND INFORMATION AND RATIONALE

In 2000, the World Health Organization (WHO) launched the Global Programme to Eliminate Lymphatic Filariasis (GPELF) to eliminate lymphatic filariasis as a public health problem by 2020. To interrupt transmission, WHO recommends therapy using combinations of two medicines delivered to entire at-risk populations through a strategy known as mass drug administration (MDA). Ivermectin and albendazole are administered in areas where onchocerciasis is co-endemic; diethylcarbamazine and albendazole are administered in areas where onchocerciasis is not co-endemic.

Results of a pilot study in Papua New Guinea suggest that triple drug therapy (ivermectin, diethylcarbamazine and albendazole) is superior to the currently recommended two-drug regimen.(1) A single dose of the triple therapy rapidly achieved complete clearance of *Wuchereria bancrofti* microfilariae from the blood of 12 individuals for at least one year post-treatment. All six individuals tested at 24 months were still amicrofilaremic, suggesting that the triple therapy might permanently sterilize adult filarial worms. Many people treated in these studies experienced transient systemic adverse events commonly associated with diethylcarbamazine or ivermectin treatment of filariasis. Adverse events were more frequent after the triple therapy than after the usual combination of two drugs. However, no serious adverse events were observed. Preliminary results from two larger clinical trials in Papua New Guinea and in Cote d'Ivoire (West Africa) are consistent with results from the pilot study. The dramatic reduction and sustained clearance of microfilaremia along with the safety profiles seen in these studies suggest that the triple drug therapy may be a useful tool for achieving the goal of eliminating lymphatic filariasis as a public health problem by 2020.

Although the studies mentioned above have clearly demonstrated the superiority of the triple drug therapy for clearing *W. bancrofti* microfilariae from the blood, more safety and efficacy data are needed before triple therapy can be rolled out on a large scale as a mass drug administration regimen in lymphatic filariasis endemic countries. WHO recommends a best practice called "cohort event monitoring" for demonstrating safety of new drug regimens for public health program use. Establishing safety through such methodology requires pre and post treatment assessments from at least 10,000 people treated with the triple therapy across multiple settings.

2.1 Fiji Specific Background

Map of Fiji:

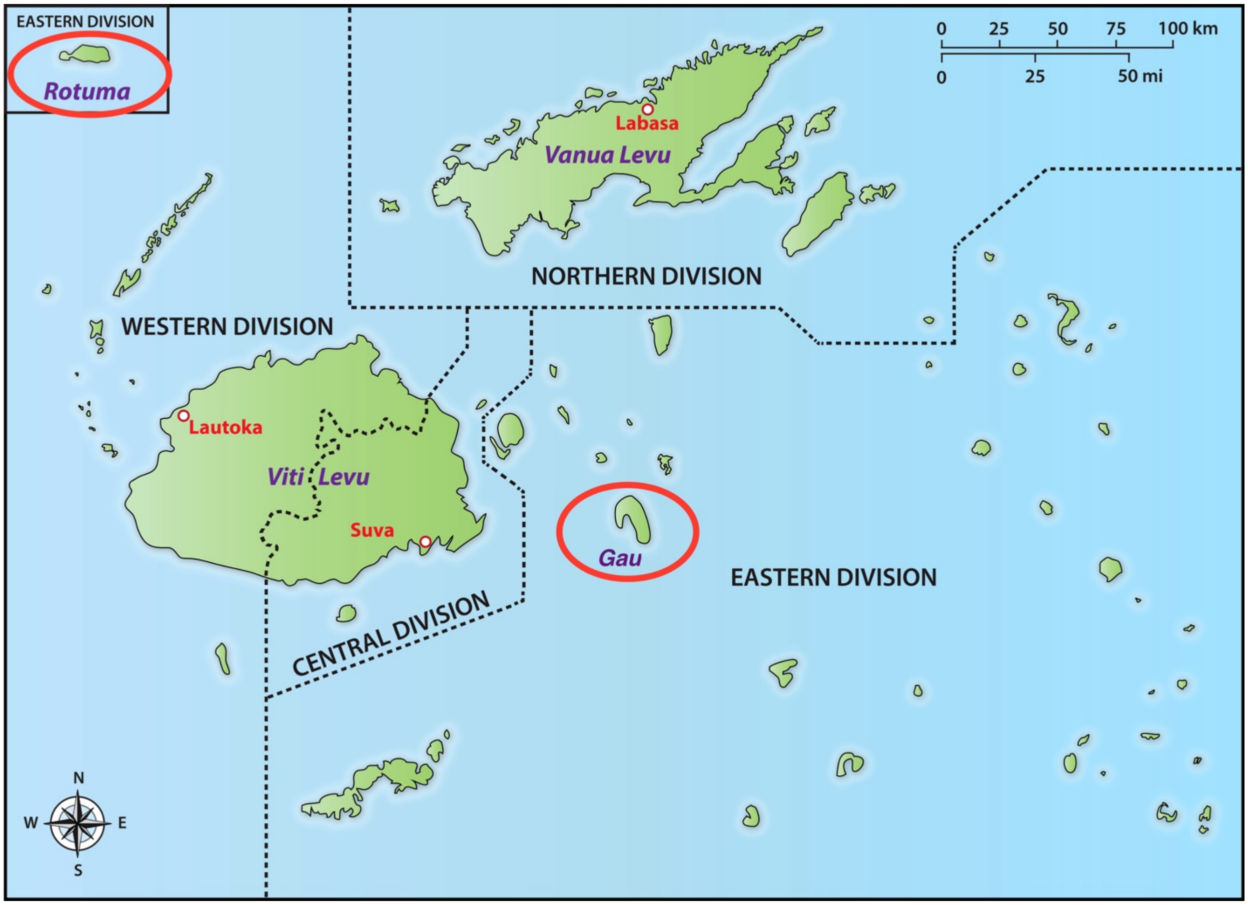

In Fiji, the occurrence of elephantiasis was described as early as 1876, but it is probable that lymphatic filariasis (LF) has plagued the population of Fiji long before that date. Opportunistic testing of patients at the Colonial Hospital was carried out in 1905 and found a mf prevalence of 25.7%. A nationwide survey of 57,000 individuals in 1956 found a 14.2% mf prevalence. Efforts in the 1960s and 1970s by Dr. Mataika and his team led to a vast reduction in LF prevalence.

Before the 1950s, control measures were concentrated on vector control and sanitation. Some experimental use of Diethylcarbamazine (DEC) occurred in the 1950s and a pilot Mass Drug Administration (MDA) was conducted in 1961. A nationwide MDA was conducted from 1969 to 1975 in which 5mg/kg of DEC were given weekly for 6 weeks and then monthly for 22 months. Microfilarial prevalence fell to less than 1% in all areas, but increased again by 1983. A study conducted between 1984 and 1991 showed that giving yearly doses of DEC for 3 years was most effective in reducing mf rates. Surveys between 1991 and 1995 showed an overall mf prevalence of 5.1%.

Fiji joined PacELF in 1999. In 2000-2001, a nationwide baseline blood survey showed that 16.6% of the tested population was LF antigen positive. Yearly rounds of MDA using DEC and albendazole began in 2002. In 2007, a survey to evaluate the 5 yearly rounds of MDA was

conducted in the four divisions: Western, Northern, Central, and Eastern. This survey showed an overall Fiji LF ICT prevalence of 9.5% and mf prevalence of 1.4%.

In contrast to other parts of Fiji, where control efforts have been largely successful, Rotuma and Gau islands have ongoing high LF Ag. The reasons for the lack of successful control of lymphatic filariasis in these communities remains unclear. Rotuma and Gau are two islands separated by significant distance but are both grouped within the Eastern Division of Fiji and are similar in size.

Rotuma Island:

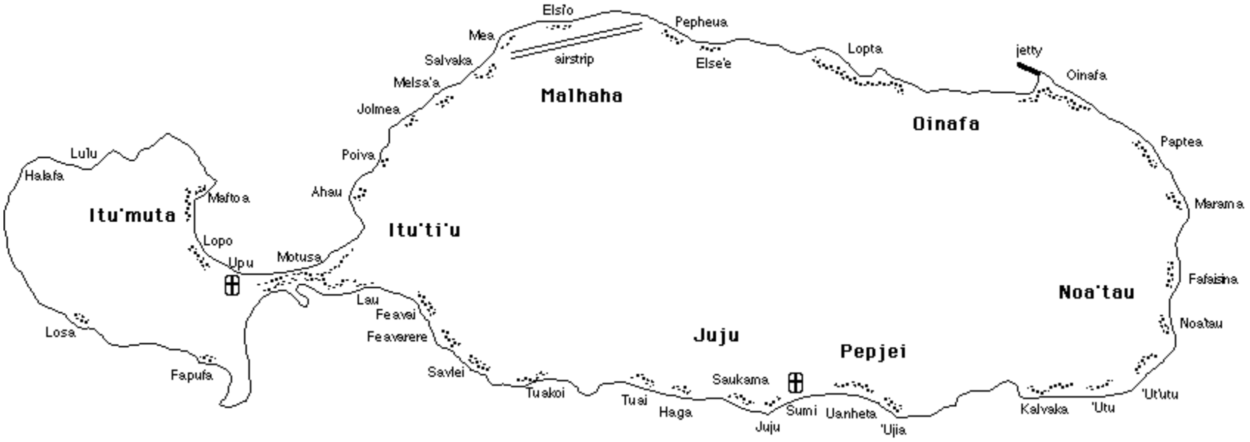

Rotuma is a volcanic island of 43 square kilometres located approximately 465 kilometres north of Fiji. It has a population of 2205 with an average village size of 69 (according to the 2007 census). Surveys undertaken since 2005 demonstrate a fluctuating LF Ag despite MDA and Test and treat programmes yearly since 2002 (except for 2007). The most recent survey in 2013 of 226 people demonstrated an ICT prevalence of 13.3%. There has been further treatment in 2014 (but with poor coverage of 41%) and 2015 with return to a higher coverage of 76%. The last mf prevalence data was from 2011 which demonstrated a high level of 9.8% with a corresponding ICT of 10.9%.

Gau Island:

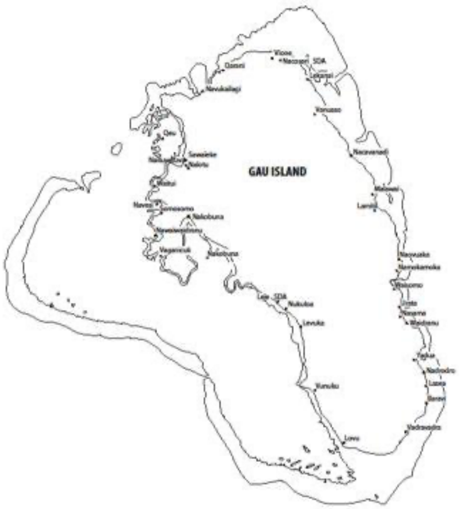

Gau is the 5th largest island in Fiji, approximately 136 square kilometres and lies 90 kilometres east of the capital Suva. It has a population of 3200 with an average village size of 97 (according to the 2007 census). They have also implemented a combination of MDA and test and treat strategy in more recent years. A test and treat program in 2012 found a ICT prevalence of 2.9% in those tested. The last cluster survey in Gau in 2013 of 129 people demonstrated an ICT prevalence of 1.6%.

The experience in Rotuma and Gau is not unique with other “hard-core” foci of individual countries maintaining a high prevalence, including in the pacific, with large areas in Papua New Guinea (PNG) reporting prevalence above 30%. It is clear in both these islands, despite multiple rounds of MDA and TNT, there remains fluctuating and persisting prevalence of LF. The program, therefore, has been in search of additional tools to help interrupt transmission in these areas and ensure that it meets the goal of elimination by 2020 (2).

Scabies is very common in Fiji and is considered an important public health issue by the Ministry of Health. A recent national prevalence study of scabies found an overall prevalence of 20% with a peak prevalence of up to 50% in young children.(3) Scabies is reported by local staff to be a common clinical problem in Rotuma and Gau. Scabies infestation frequently leads to secondary bacterial infection of the skin (impetigo) which in turn can cause serious complications such as bloodstream infection and kidney and heart disease. (4-7) This tremendous burden of infectious disease has been neglected, largely because no effective intervention was available. However, MDA with ivermectin represents a highly promising strategy to control scabies because it can break the transmission cycle. A recently published trial of ivermectin MDA for the control of scabies in Fiji reports a 94% reduction in scabies prevalence at 12 months compared to 32% in the standard care arm (permethrin to index case and contacts only). (8) There was a concomitant reduction in impetigo prevalence.

Ivermectin kills the scabies mite but not its eggs. Therefore, practice has been to give a second dose of IVM 1-2 weeks later to kill mites from any newly hatched eggs. For this reason, the published trial in Fiji used 1 dose of IVM for all participants and a second dose for those with scabies at baseline. However, a retrospective review of scabies cases presenting to a health clinic

in areas of LF MDA with IVM (one dose) and ALB in Zanzibar over a period of 6 years, demonstrated a decrease in scabies prevalence during this time period. (9) A single dose of IVM has clear advantages for the rollout of MDA for scabies as a public health program and its integration with other NTD programs. This study aims to review the question of whether one dose of IVM is not inferior to 2 doses of IVM.

STH are also common in Fiji and is a priority of the Ministry of Health; a study conducted on the island of Taveuni found a prevalence of intestinal worms between 14% and 33% in school children. (10) A previous unpublished survey of STH revealed a prevalence in women of childbearing age of 20% (personal communication J Samuela).

With the high burden of LF that has been recalcitrant to previous MDA with DEC and ALB, and the high prevalence of scabies and STH, Fiji is an ideal site to obtain data on the efficacy and safety of triple therapy MDA for LF in conjunction with its effect on scabies, impetigo and STH.

3 POTENTIAL RISKS AND BENEFITS

3.1 Risks of Blood Draw

Blood collection via finger prick is considered to be minimal risk and little or no discomfort is anticipated. This risk of infection is minimized by the use of standard sterile techniques. On occasion a participant may faint during or after the finger prick. Study personnel will be alert to participant reactions after the blood collection and will provide aid as needed.

3.2 Risks of Study Drugs

The combinations of ivermectin plus albendazole or DEC plus albendazole are widely used for MDA. There also have been clinical trials of DEC plus Ivermectin and for triple drug therapy that show no significant drug interactions.(1) Risks of each drug separately, with some indication of how likely these are to occur, are summarized below:

Diethylcarbamazine (DEC): The most common side effects reported are itching and swelling of face, headache, joint pain, unusual tiredness or weakness. These are transient. Less common are dizziness, nausea or vomiting. Fever, painful and tender glands in groin, neck and armpits or skin rash can occur, and are usually associated with high burdens of infection as judged by the level of blood microfilaremia.

Albendazole (ALB): The most common side effects reported are headache, nausea, stomach pain and vomiting are most common, and usually associated with heavy soil-transmitted helminths infections. Severe allergic reactions occur rarely, and include rash, hives, itching, difficulty breathing, tightness in the chest, swelling of the mouth, face, lips, or tongue, dark urine. Mild elevation in liver transaminases can occur, but normalize with cessation of treatment. These AEs are usually associated with prolonged ALB therapy.

Ivermectin (IVM): The most common side effects reported are diarrhea, dizziness and nausea. Rare side effects include rash, hives, itching, difficulty breathing, chest tightness, swelling of the mouth, face, lips, or tongue, eye pain, fainting, fast heartbeat. Mild decrease in leukocyte counts, elevated liver function tests, and cardiovascular effects that included tachycardia and orthostatic hypotension have been described. Infrequently, treatment can exacerbate bronchial asthma. These AEs are also associated with prolonged therapy. Ivermectin has previously been used and its side effects monitored in the Fijian population as part of the research project SHIFT. The most common symptoms reported were itch, headache, abdominal pain, joint pain and dizziness. (8)

Permethrin 5%: The most common side effects reported are generally mild and transient. In clinical trials, burning and stinging following application of the cream on the skin is reported in 10% of patients and associated with the severity of infestation. Itch was reported in 7% and erythema, numbness, tingling and rash reported in less than 2%. Other adverse events reported but not confirmed as associated include headache, fever, dizziness, abdominal pain, diarrhea, nausea and/or vomiting, and seizure. (11)

3.3 Potential Participant and Community Benefit

Infected participants, who sign an informed consent, will be treated for the LF infection. LF transmission to the community will be reduced by participation in either treatment arm. A broader community benefit may be facilitated by the triple drug regimen as it is believed the triple drug regimen has the potential to markedly reduce the number of MDA treatments needed to achieve transmission interruption and elimination of LF.

Both regimens provide treatment for intestinal worms, and the triple drug treatment has the added benefit of providing an effective treatment for scabies, head lice and strongyloides (another intestinal worm infection).

If the triple drug intervention proves successful, the triple therapy is likely to be adopted in many LF endemic areas globally. Dr Samuela, Dr Kama and Dr Tuicakau are investigators from the Fijian Ministry of Health, and with support from investigators based in Australia, will be able to action the implementation of new treatment guidelines for individuals and MDA programs across Fiji. The results from the Fiji site will be combined with other sites so that aggregated conclusions can be shared with the World Health Organization for further global distribution and subsequent implementation.

3.4 Subject Participation and Cost

Participation is voluntary and participants may decline participation without consequences. There will be no cost to the subject to participate in the study and they will not be paid for their participation. The study will cover cost associated with laboratory test, study drugs, and clinical monitoring.

3.5 Compensation for Injury

The study drugs have been widely used for treatment of lymphatic filariasis and it is anticipated that injury resulting from treatment will be rare. In the event that a participant experiences a serious adverse event (SAE) attributable to study treatment, the project will help in supporting the medical treatment and/or hospitalization required.

In addition, DOLF have trial insurance that will provide compensation to participants for serious trial-related events if required.

4 STUDY DESIGN AND OBJECTIVES

4.1 Study Objectives

To determine the frequency, type and severity of adverse events following triple-drug therapy (IVM+DEC+ALB, IDA) compared to the standard two-drug treatment (DEC+ALB, DA) in infected and uninfected individuals in a community.

Secondary Objectives

1. To compare the efficacy of IDA (3 drug therapy) to DA (2 drug therapy) administered in communities for clearance of mf and filarial antigenemia (Ag).
2. To assess the effect of intensity of filarial infection on the frequency and severity of adverse events.
3. To evaluate the impact of IDA MDA on scabies and impetigo prevalence and the effect of 1 versus 2 doses of ivermectin.
4. To evaluate the impact of IDA MDA on STH (hookworm, ascaris, trichuris and strongyloides) prevalence.
5. To compare acceptability and feasibility of IDA MDA to DA MDA in communities at risk of LF, scabies and STH.

4.2 Study Design

This trial will be an open labelled three-armed study (see Figure 4.2.1). In addition to LF MDA there will be a combined scabies treatment or MDA (see Figure 4.2.2) depending on eligiblity criteria (see section 4.9). The three arms are:

1) MDA with the currently used combination of DA (two-drug regimen).
2) MDA with IDA (triple drug therapy) with one dose of ivermectin (day 0).
3) MDA with IDA (triple drug therapy) with two doses of ivermectin (days 0 and 8).

There are multiple stages to the study over a period of approximately 12 months. Key points are Community Awareness in the weeks before the study begins, Treatment Day 0, AE monitoring Day 1-7, Treatment Day 8, 4 week Acceptability Study and repeat stool sampling, and 12 months repeat of baseline assessments. Please see Appendix 1 for further details.

Figure 4.2.1: Day 0-8 LF MDA

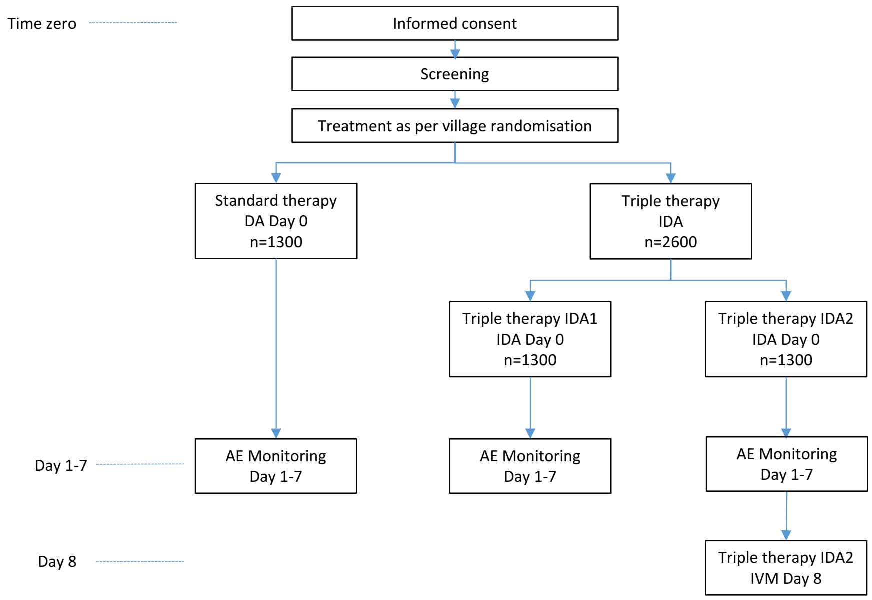

DA: Diethylcarbamazine (DEC) and Albendazole (ALB)
IDA: Ivermectin (IVM), DEC and ALB
MDA: mass drug administration
AE: Adverse event

Figure 4.2.2: Day 0-8, LF MDA and Scabies Treatment/MDA

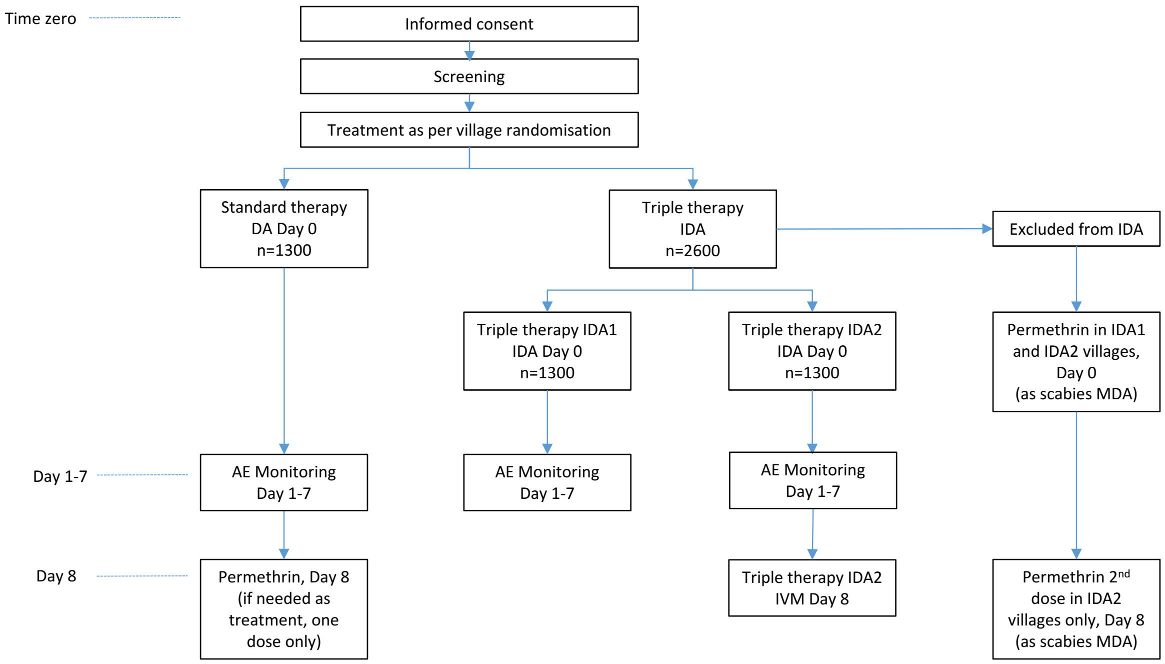

DA: Diethylcarbamazine (DEC) and Albendazole (ALB)
IDA: Ivermectin (IVM), DEC and ALB
MDA: mass drug administration
AE: Adverse event

4.3 Study Screening and Enrolment

Physicians working in the Eastern Division of Fiji are motivated by the ongoing LF burden in this region to be involved in the study. The two islands have been chosen due to their historical and anecdotal high LF prevalence as well as both having the necessary logistical support to conduct this study including an airstrip and established local health clinic.

4.4 Preparatory Activities

Social Mobilization

Prior to the administration of the drugs, intense social mobilization activities will be conducted to ensure maximum community participation. This will include development and distribution of key messages that will emphasize the acceptance and swallowing of the drugs along with their benefits and safety.

It is expected these messages will be delivered in the form of village meetings, posters in each village centre, information packs available through each village headman and radio announcements.

Household Enumeration and Census

In the month prior to the study team arrival, health workers who are permanent residents of the islands, will work with the community leaders to compile a list of members of their community with details including name, age, sex, house number and village. This will be accompanied by map of each village referencing the position of each house and its number. This list will be used as a village census to crosscheck enrolment and the map will be used to locate households for those potential participants that don't present and AE follow up as required.

4.5 Pre-Treatment Assessments

Prior to the study team arriving, local health workers will be communicating with the community regarding the study, steps involved and anticipated timeframe. These health workers have been used previously in LF MDA programs and are known by the local community. On the week prior to arrival a stool pot with collection instructions will be provided by the village health worker (a member of the community) to randomly selected individuals. They will be asked to collect a fresh (within 12 hours) sample of stool to bring to the designated Study Station on the Treatment day in their village.

The first visit to the village by the study team will be referenced as "Treatment Day" or "Day 0". All members of the village will be invited to participate. The whole village will be treated in one or consecutive days depending on village size with a maximum enrolment of 75 people per day. Prior to treatment there will be a series of steps (see Figure 4.5.1 and 4.5.2 below). First, the participants will be asked to give informed consent to be involved in the study (see section 4.6 for more details). They then will be enrolled with a unique study ID number allocated (see Appendix 2) and their name cross-referenced with village census. This will likely be done by the village health worker. Those that have been randomly chosen to participate in the STH component will be asked to provide their stool pot (with sample collected at home). This will be processed out of sight, Kato-katz will be performed and the remaining sample will be measured and have preservative added. This will enable PCR testing at a later stage in Australia. They will then move onto a nurse, medical/nursing student or experienced project coordinator who will collect their blood for the rapid FTS. Whilst waiting for the result they will be asked to provide a limited history

and undergo examination including skin assessment. If negative, final check of contraindications to treatment before being given the allocated treatment as per their village randomisation. If positive, the participant will have an additional finger prick bleed for mf testing before being given the allocated treatment as per their village randomisation.

For those members listed in the census that don't attend the clinic, a home visit will be undertaken to ask them if they would like to be involved in the study.

Figure 4.5.1: Treatment Day Flow

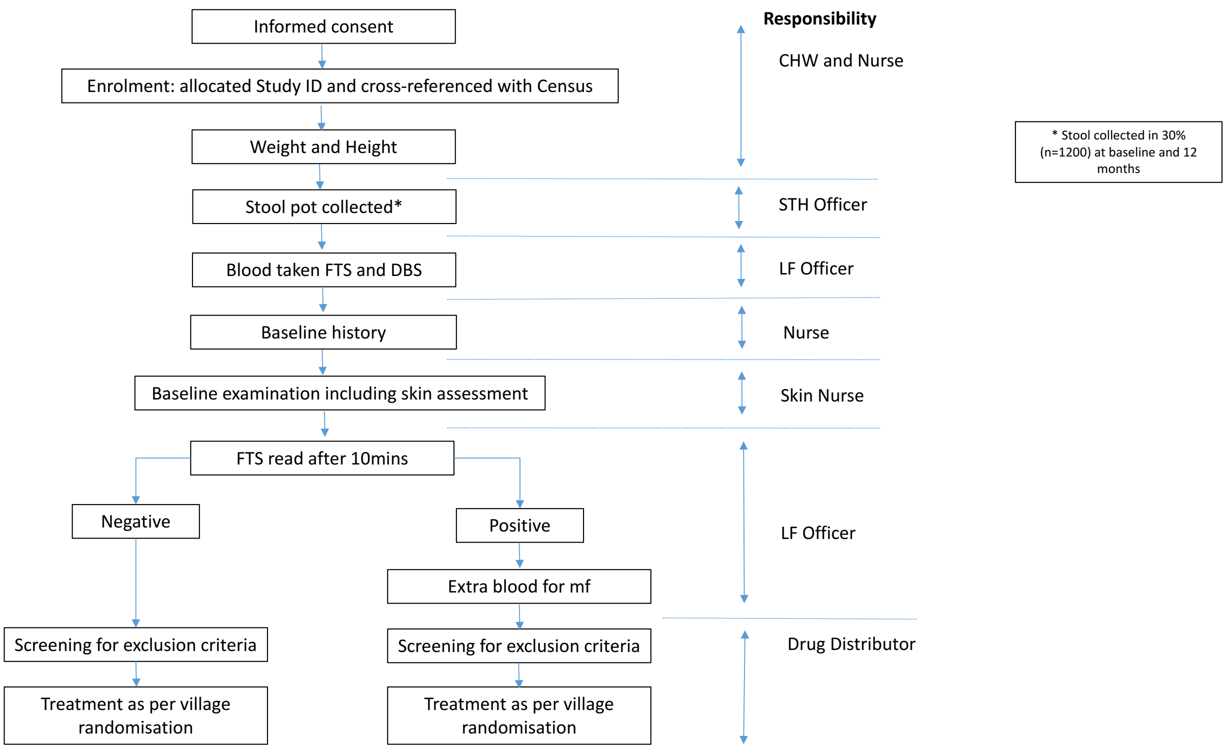

ID: identification number
FTS: Filariasis Test Strip
DBS: Dried Blood Spot
mf: microfilariae

4.6 Informed Consent

Prior to performing any study specific procedure, a signed consent form will be obtained for each subject. For subjects below 18 years of age, consent must be obtained from a parent, legal guardian, or person with power of attorney. For those children 13 years and older and less than 18 years a signed consent form will also be required. The consent form will describe the purpose of the study, the procedures to be followed, and the risks and benefits of participation. The village health worker and/or a study nurse will conduct the informed consent discussion and will check that the subject and their legally acceptable representative comprehend the information provided and answer any questions about the study. In the event that a subject is unable to read, another villager with sufficient reading and writing skills who is not involved in the implementation of the study, will act as a witness to the consenting process. Consent will be voluntary and free from coercion. The investigator that conducted the consent discussion will also sign the informed

consent form. A copy of the consent form will be given to the subject or their legally acceptable representative and the fact that the subject has been consented to the study will be documented in the subject's record. Please see Appendix 6 and 6A for a copy of the consent forms.

4.7 Screening for Filarial Antigenemia and Microfilaria

Approximately 75µl of capillary blood from each eligible individual will be collected via finger prick to be deposited on the rapid diagnostic test Filariasis Test Strip (FTS, Alere™, WHO approved)) for LF antigen detection in the field. The strength of the antigen line will also be scored from 0 – 3+ corresponding with intensity of infection. An additional 60µl of capillary blood from the same finger prick will be deposited on filter paper and dried for subsequent serological evaluation for the diseases under study - LF, scabies and STH. This dried blood spot (DBS) will be processed at Mataika House, after training is provided as required. If the Ministry of Health and Medical Services (MOHMS) requests additional serological tests, the DBS may be shipped to international laboratories, including the Centres for Disease Control and Prevention (CDC), and all results will be shared with the MOHMS. No HIV or genetic testing will be performed. Residual DBS will be destroyed upon completion of testing for FIT purposes.

Participants with positive FTS tests will have additional blood taken for mf testing (60µl measured volume blood smear- 3 lines, prepared according to the project SOP) collected by the finger prick method.

Universal precautions for individuals collecting and working with blood samples to include proper disposal of contaminated materials (test strips, lancets, capillary tubes, blood film slides) will be in accordance with the guidelines prescribed by the local health authorities.

4.8 Baseline Physical Examination and Pre-Treatment Adverse Event Monitoring

A medical history and examination to obtain baseline information and screen for contraindications to treatment will be undertaken. This process will also include the same questions and clinical monitoring that will be part of AE monitoring to establish symptoms and signs prior to treatment. Please see full details of this assessment in Appendix 2.

4.9 Eligibility

All community members will be invited to participate in the study.

Inclusion Criteria for Treatment

As a community-based study of mass drug administration, all community members, that have given written informed consent to participate, are able to be included in the study.

However due to certain restrictions for some of the medications, there will be modifications to the intervention drug protocol for LF and scabies described below and outlined in figures 4.9.1.1 – 4.9.1.6.

- If a child is <5 years but ≥2 years old they will receive the following according to their village randomization:
    - DA dual therapy: DA to all on day 0, and for those identified with scabies or a household contact of someone identified with scabies topical permethrin 5% on day 8.

- IDA triple therapy, 1 dose IVM: DA to all on day 0, and 1 dose of permethrin 5% to all on day 0 (since there is no safety data for IVM use under 5 years or less than 15kg).
- IDA triple therapy, 2 doses IVM: DA to all on day 0, and 2 doses of permethrin 5% to all on day 0 and day 8.

- If a child is <2 years or <15 kg they will receive the following according to their village randomization:
    - DA dual therapy: topical permethrin 5% only to those identified with scabies or a household contact of someone identified with scabies on day 8 (since this age group has been excluded from DA studies in the past due to limited safety data).
    - IDA triple therapy, 1 dose IVM: 1 dose of permethrin 5% to all, day 0.
    - IDA triple therapy, 2 doses IVM: 2 doses of permethrin 5% to all, day 0 and day 8.

- If a woman is pregnant or has delivered a baby in the last 7 days and is breastfeeding, she will be given the following according to her village randomization:
    - DA dual therapy: topical permethrin 5% on day 8, if she is identified as having scabies or being a household contact of someone with scabies
    - IDA triple therapy, 1 dose IVM arm: 1 dose of permethrin 5%, day 0
    - IDA triple therapy, 2 doses IVM arm: 2 doses of permethrin 5%, day 0 and day 8.

- If the participant has a severe illness or allergy to IVM, DEC or ALB they will be given the following according to their village randomization:
    - DA dual therapy: topical permethrin 5% on day 8, if they are identified as having scabies or being a household contact of someone with scabies
    - IDA triple therapy, 1 dose IVM arm: 1 dose of permethrin 5%, day 0
    - IDA triple therapy, 2 doses IVM arm: 2 doses of permethrin 5%, day 0 and day 8.

Figure 4.9.1.1: Village Randomised to DA, LF MDA Algorithm

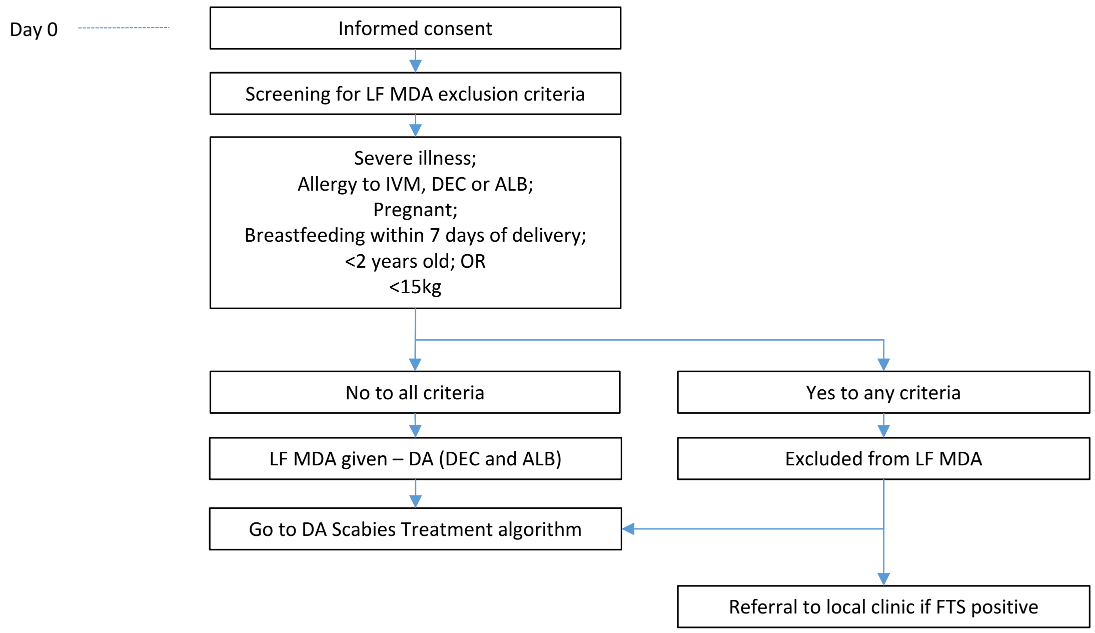

Figure 4.9.1.2: Village Randomised to DA, Scabies Treatment Algorithm

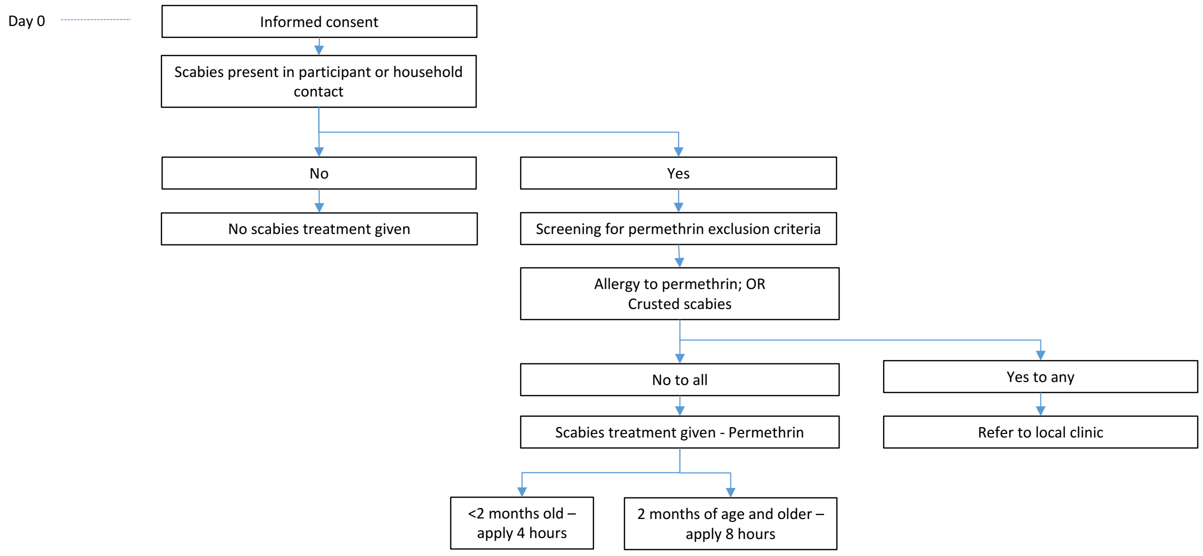

Figure 4.9.1.3: Village Randomised to IDA1, LF MDA Algorithm

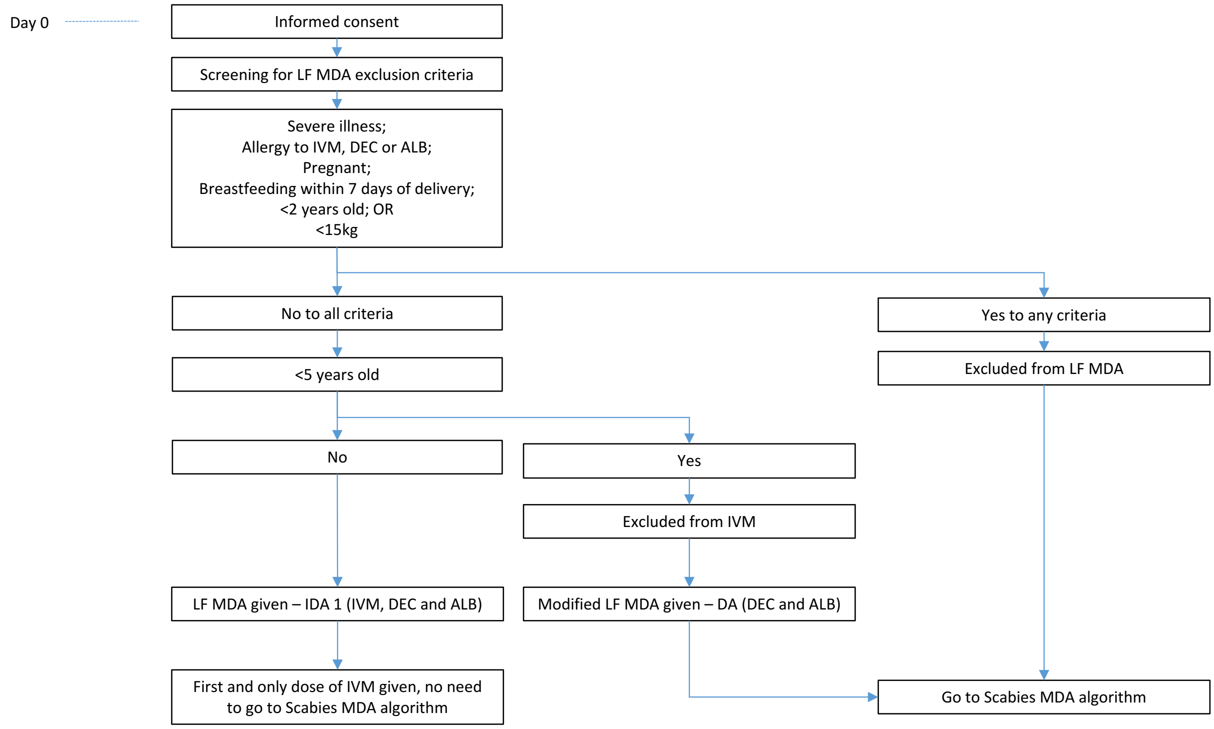

Figure 4.9.1.4: Village Randomised to IDA1, Scabies MDA Algorithm

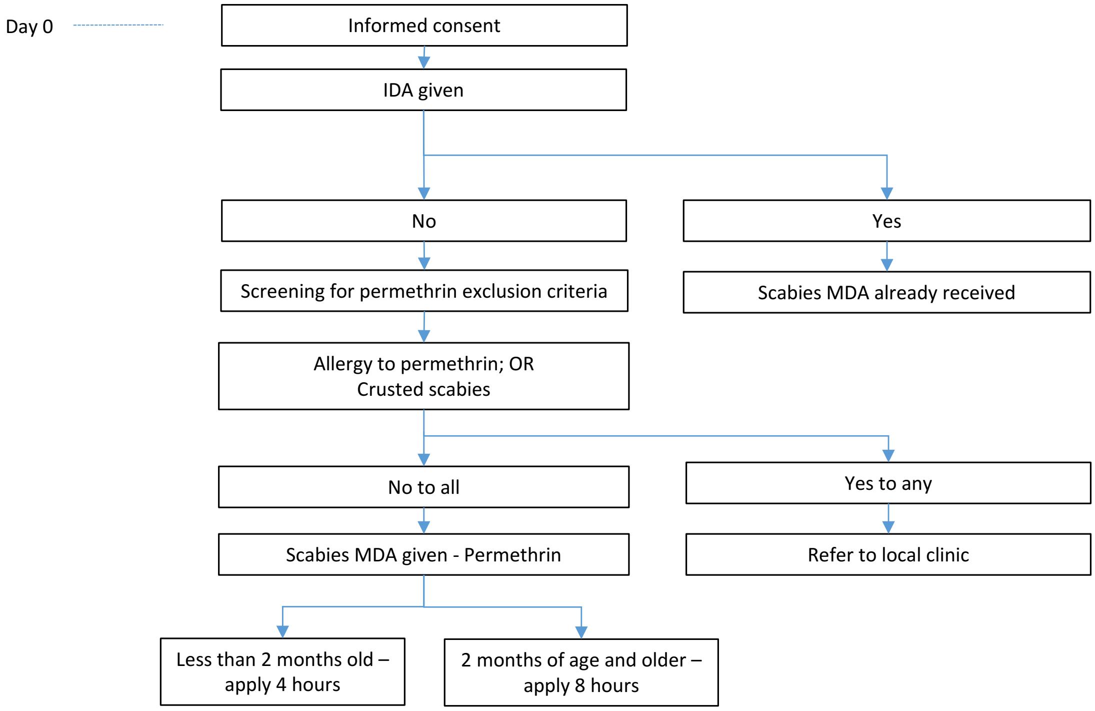

Figure 4.9.1.5: Village Randomised to IDA2, LF MDA Algorithm

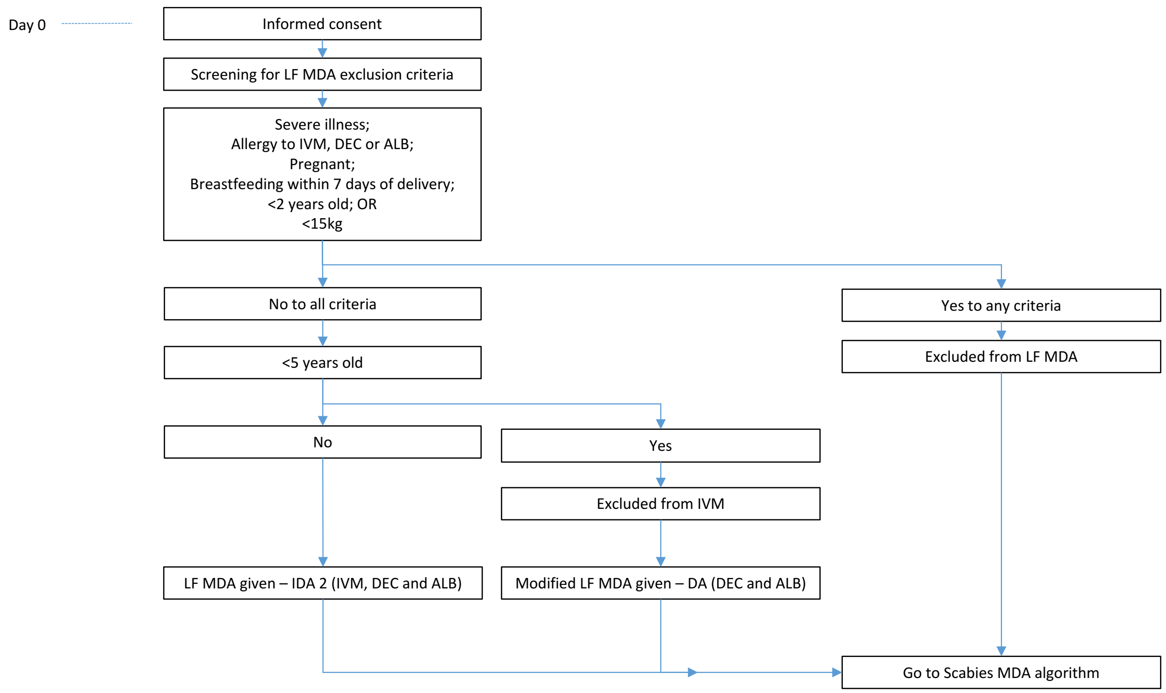

Figure 4.9.1.6: Village Randomised to IDA2, Scabies MDA Algorithm

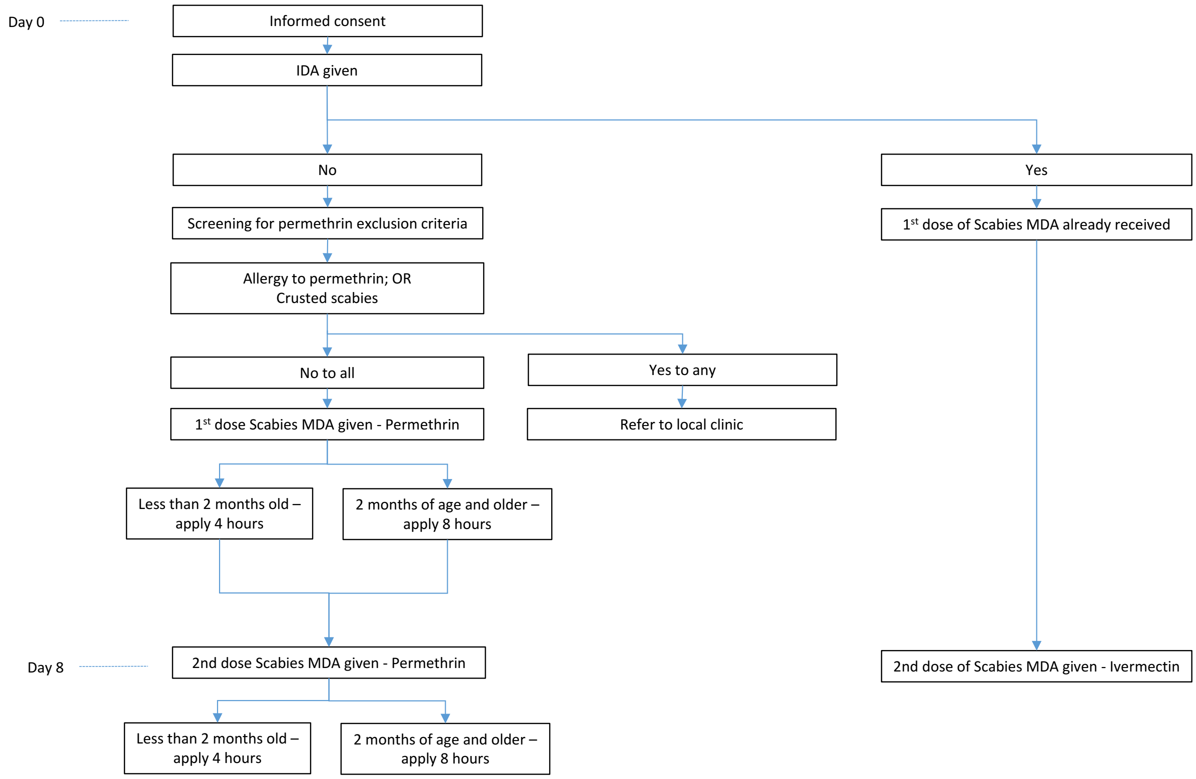

Exclusion Criteria for Treatment

Patients are ineligible to participate in receiving LF MDA (DA or IDA) as part of the trial, if they have any of the following:

1) Severe illness (chronic renal insufficiency, severe chronic liver disease, or any illness that is severe enough to interfere with activities of daily).
2) History of previous allergy to any of the components for the MDA drug regimen.
3) Pregnant
4) Breastfeeding within 7 days of delivery
5) Less than 2 years old
6) Less than 15kg.

Patients are ineligible to participate in receiving permethrin if they have any of the following:

1) Crusted scabies
2) History of allergy to permethrin.

If there is a clinical diagnosis of crusted scabies made as part of the study skin assessment, these participants will be referred to the local clinic and be recommended to receive 2 doses of IVM 1 week apart in combination with twice weekly permethrin cream for 1 month. Since crusted scabies is highly contagious, increased treatment efforts will be given to these individuals to attempt to reduce the impact of the overall effect of the IDA MDA on community scabies prevalence.

If the participant is identified as having LF or STH but is ineligible for treatment on treatment day, they will be referred to the local clinic for follow up in the future if/when it is likely that the reason for exclusion from treatment has resolved.

4.10 Pregnant and breastfeeding Females

Pregnant females and women who have delivered in the last 7 days and are breastfeeding will not be eligible to receive ivermectin, diethylcarbamazine or albendazole because of the unknown effects of the drugs and drug combination used in this study. However, they can still be included in the study for demographics, history, examination including scabies assessment and LF testing.

Female participants of child-bearing age will be asked whether they are pregnant. If unsure, a pregnancy test by urine dipstick will be offered. If a participant is pregnant, she will have her treatment modified as above in 4.9. If a participant is unsure but does not want to be tested, the participant will be treated as pregnant. To keep the results of the testing confidential it was recommended by Fijian colleagues that drug administration occur in a private portable booth so that the status of pregnant females will not be known by other family members.

Similarly, female participants will also be asked if they have had a child in the last 7 days and are breastfeeding. These women will also have their treatment modified as per 4.9 above.

If women are excluded from receiving DA or IDA, they can still safely use permethrin cream if applicable i.e. if they have been randomized to DA treatment and they or a household member

have scabies identified, or if they have been randomized to IDA treatment regardless of scabies identification.

4.11 Randomization – Community Assignment

Treatment arms will be randomised on a village level. Randomisation in the three study arms will be stratified by island with an allocation ratio of 1:1:1. The randomization schedule will be created by an independent statistician (Clinical Epidemiology and Biostatistics Unit, MCRI) using Stata software and a randomization protocol in line with the intuitional standard operating procedures.

4.12 Withdrawal

Participation in this project is completely voluntary, and participants may terminate participation at any time. Also if the well-being of the participant is compromised in any way, based on the opinion of the investigator, the subject can also be withdrawn from the study. Even if the participant leaves the project early, we will encourage them to contact us at any time within the month after treatment to report any possible study-related AEs.

4.13 Efficacy and Effectiveness of IDA vs DA

One year post MDA, all individuals resident in the communities during the study period will be assessed for LF in the same manner undertake at baseline i.e. FTS and DBS on all and if positive, mf by blood smear.

Similarly, all individuals at 12 months will have a skin assessment undertaken for review of effect on scabies and impetigo. A random 30% of participants will also have a stool sample collected to review the different MDA treatments on stool parasitology.

At 12 months all participants will be asked epidemiological questions related to risk factors for disease transmission that will assist in interpretation of effectiveness across treatment arms. Please see Appendix 2 – Participant Data Collection Form.

4.14 Retreatment

Participants with new *or* persistent infection (antigenemia or microfilaremia) at 12 months will be re-treated as part of the community MDA.

Aggregated safety data was presented to the WHO in 2017. Based on comparable safety between DA and IDA, WHO have recommended that Fiji adopts IDA for future rounds of LF MDA. The National Taskforce for LF Elimination in Fiji, have endorsed this recommendation. As such, all participants at the 12 month review will be offered IDA MDA. Since the effectiveness of one dose of ivermectin is yet to be evaluated, any participant who is identified as having scabies or their household member is identified as having scabies on skin examination, will be offered treatment with topical permethrin 5% (1 dose).

4.15 Termination of the Study

There are no pre-specified criteria for terminating the study early.

The study continuation should be reviewed by the DSMB if a treatment arm shows an increase from the expected baseline drug related SAE of 1:1000. The final decision to stop the trial is left to the recommendation of the DSMB. If the DSMB recommends discontinuation or modification of the study, the Chair of the DSMB will meet or speak to the DOLF Project Team and Fiji Project Team at the earliest opportunity to review the basis for the recommendation.

4.16 Triple Drug Regimen Acceptability

It is important to understand the community's acceptability of the 3 drug MDA regimen as well as gain insight into the feasibility of administering this strategy in the future. To gain a complete picture of the community's view approximately 150 people per treatment arm will be asked to complete a survey. Participants will be randomized by household taken from a sample of at least 8 villages. To complement this survey, a series of focus group discussions in the community as well as key informant interviews are proposed with community leaders, health personnel and drug distributors to assess perceptions about the 3-drug versus the 2-drug regimen. This will be conducted approximately 1 month after treatment day. Further details can be found in Appendix 7.

At the 12 month visit Acceptability will be assessed using a focused questionnaire see Appendix 2 – Participant Data Collection Form. The questionnaire will be administered to one individual per household, with additional individuals recruited where possible depending on study workflow and timing.

5 INVESTIGATIONAL PRODUCT

Each of the drugs used in this study is approved for human use and has a prior history of use in the treatment of Lymphatic Filariasis or scabies.

5.1 Study Drug Background

Albendazole (ALB) has been known to cause degenerative alterations in the tegument and intestinal cells of the worm by binding to the colchicine-sensitive site of tubulin, thus inhibiting its polymerization or assembly into microtubules (12). The loss of cytoplasmic microtubules leads to impaired uptake of glucose by larval and adult stages of the parasite, and depletes glycogen stores. Degenerative changes in endoplasmic reticulum and mitochondria of the germinal layer, and the subsequent release of lysosomal enzymes result in decreased production of adenosine triphosphate, which is the source of energy required for survival of the helminth. Due to diminished energy production, the parasite is immobilized and eventually dies. Adverse events are uncommon in persons who are treated with a single dose of albendazole (apart from AEs that result from parasite death). Some patients report mild gastrointestinal AEs such as nausea after ingesting the tablet.

Ivermectin (IVM) is an avermectin compound of macrocyclic lactones derived from the bacterium *Streptomyces avermitilis* (13). The mechanism by which IVM kills LF microfilariae is not known with certainty, but the drug interferes with glutamate gated ion channels that can affect parasite contractility and release of immunomodulatory molecules by the parasite (13). IVM also has a direct effect on the central nervous system and muscle function of worms as it enhances strength of inhibitory neurotransmission pathways. The main concern with the use of IVM in animals and humans is neurotoxicity, which can be manifest as ataxia. Neurotoxicity has not been observed in humans given single dose IVM for LF or other parasitic infections (14). IVM has been used to treat millions of people with LF and onchocerciasis. Peak IVM serum concentrations are reached approximately 4-5 hours after administration. The half-life of IVM in various populations ranges from 12 to 56 hours (15). There is no evidence of drug: drug interaction between ALB and IVM (16). IVM can cause nausea, dizziness and occasionally pruritus, but these are infrequent, transient and usually mild. Serious adverse events have occurred in patients with heavy *Loa loa* infections.

DEC (diethylcarbamazine citrate) is an anthelminthic drug that is structurally distinct from ALB and IVM. DEC inhibits arachidonic acid metabolism by LF, and inducible nitric oxide synthase and the cyclooxygenase pathway may be essential for activity in vivo. DEC also has anti-inflammatory properties. The mechanisms of action of DEC remain poorly understood. Its ability to kill mf and adult worms depends on the host immune responses since the drug has little direct activity on parasites in vitro. The drug has potent activity against LF microfilaria. DEC has about 50-70% efficacy in killing or sterilization of adult worms. The drug is rapidly absorbed from the gastrointestinal tract, has a serum half-life of 12 to 14 hours, and is excreted in the urine with little modification by liver metabolism. Adverse events from DEC are unusual apart from those that result from killing filarial worms.

Permethrin 5% cream is a topical scabicidal agent for the treatment of scabies infestation. It is a pyrethroid, which is also active against other pests including lice, ticks, fleas and other arthropods. It acts on the sodium channel of the nerve cell membrane to disrupt repolarization leading to paralysis of the pests. It is rapidly metabolized to inactive components which are excreted primarily in the urine. However, in absorption studies in moderate to severe scabies infestations the amount that is absorbed is 2% or less. Most adverse events are mild and transient and related to skin irritation following application. (11)

Product Supply and Storage

Only WHO approved drugs will be used in this study. DEC and albendazole will be provided by WHO. Ivermectin at baseline will be purchased from Merck, Sharp and Dohme (MSD) Australia. Permethrin will be purchased from Pharmatec, Fiji with product manufactured by Glenmark.

The donation program for ivermectin has been extended to include all regions endemic for LF. As such, ivermectin supply will be provided by WHO donation program if possible. If not possible to source donated ivermectin, it will be purchased from Merck, Sharp and Dohme (MSD) Australia.

All three study drugs (IVM, DEC and ALB) are approved and distributed globally by WHO as part of GPELF. Detailed information for each drug is available from the pharmaceutical manufacturer. All products should be maintained between18-30 °C.

6 STUDY PROCEDURES/EVALUATIONS/SCHEDULE

Residents of Rotuma and Gau will be invited to participate in the survey by letter followed by a visit from the local zone nurse. After informed consent, participants will be asked to provide basic demographic information such as age and gender, as well as a brief medical history.

6.1 Triple Drug Therapy (IDA) and Two-Drug Therapy (DA)

The triple-drug combination will consist of a IVM (200 µg /kg), DEC (6mg/kg) and ALB (flat dose of 400 mg). Triple-drug therapy will be randomized by village into half that receive one dose of IVM and the other half that will receive a second dose of ivermectin 8 days following the first. The two-drug combination will consist of a single dose of DEC (6mg/kg) and ALB (flat dose of 400 mg). All participants will be weighed to ensure accurate dosing of study medication (see Ivermectin and DEC dosing chart below). Drugs will be given after confirming informed consent obtained and review of exclusion criteria. The study population will be encouraged to eat before swallowing the medicine (without chewing the tablets) with a glass of water. For participants who can't swallow whole tablets, they will be crushed and mixed with yoghurt/custard or equivalent. Vomited doses will be replaced. Drug administration will be supervised (directly observed treatment or DOT) to ensure that all enrolled individuals swallow the drugs. There will be one supervisor of drug administration per study team.

Villages allocated to DA therapy will receive standard of care for scabies in Fiji, that is, permethrin to index cases and their household contacts. This will be provided with instructions for application on day 8, to ensure no interference with AE monitoring following DA therapy. Permethrin will be given to all excluded participants from the IDA treatment arm to complete MDA for scabies within that community. Furthermore, a second dose of permethrin on day 8 will be given to excluded participants from the IDA 2 villages to mimick the 2 doses of IVM that these communities receive.

The permethrin cream should be applied all over the body from neck to toe and washed off after a minimum of 8 hours (recommended to apply at night and washed off in the morning). The exception is that for infants <2 months of age the cream should be washed off after 4 hours. A demonstration of how to apply this cream at home will be given when the cream is distributed.

DOLF IDA DOSING CHART (by weight or age)	
**Albendazole (ALB)**	
**≥15 kg**	1 tablet (400 mg)
**Diethylcarbamazine citrate (DEC)**	(dosing 6mg/kg)
**15-25kg**	1 tablet (100 mg)
**26-41kg**	2 tablets (200 mg)
**42-58kg**	3 tablets (300 mg)
**59-75kg**	4 tablets (400 mg)
**76-92kg**	5 tablets (500 mg)
**≥93kg**	6 tablets (600 mg)
**Ivermectin (IVM)**	(dosing 200 ug /kg)
**15-23kg**	1 tablet (3 mg)
**24-38kg**	2 tablets (6 mg)
**39-53kg**	3 tablets (9 mg)
**54-68kg**	4 tablets (12 mg)
**69-83kg**	5 tablets (15 mg)
**84-98kg**	6 tablets (18 mg)
**≥99kg**	7 tablets (21 mg)
**Permethrin**	(dosing topical 5%)
**Age <2 months**	Apply for 4 hours
**Age ≥2months**	Apply for 8 hours

6.2 Lymphatic Filariasis Testing

All participants 2 years and older will have capillary blood obtained via finger-prick for LF antigenemia testing – FTS and DBS. A FTS result will be available in 10 minutes. If negative, they will proceed to allocated treatment. If positive, a further finger-prick blood test for a mf count will be undertaken prior to treatment. Once this is obtained the allocated treatment can be given. These procedures will be undertaken at baseline and at 12 months on all participants 2 years and older. This will provide information on efficacy of treatment, enable linkage of AEs with severity of infection and help to determine if there are new cases that represent ongoing transmission in the community.

6.3 Skin Examination

Scabies and impetigo prevalence will be determined by skin examination of all participants at baseline and again at 12 months. The WHO Integrated Management of Childhood Illness (IMCI) skin algorithm will be used as the guideline for clinical examination and diagnosis of scabies and impetigo.(17) As in SHIFT, scabies will be defined without the use of microscopy or dermatoscopy, but rather expert examiners will make a diagnosis on the basis of typical clinical findings, that is, pruritic inflammatory papules in a typical distribution.(8) Impetigo will be defined

as papular, pustular or ulcerative lesions surrounded by erythema with or without the presence of crusts, pus or bullae.

6.4 Stool Collection

At baseline and 12 months, 30% (n=1200) of randomly chosen participants will be given a stool container and instructions for bringing a fresh collection to the Study station on their village ‘Treatment Day’. They will be requested to provide their specimen of stool at baseline and Month 12 treatment day. If the same participants at 12 months cannot or decline to provide a second stool sample, then other randomly chosen participants will be asked to provide a stool sample. These samples will allow a review of the community effect of MDA for STH.

In addition, at 4 weeks following treatment, randomly selected individuals with a positive stool sample at baseline will be asked to provide another sample. The total sample size at 4 weeks is 150 per treatment arm. This sample size and timeframe will allow a review of the individual effectiveness of DA versus IDA treatment on STH before re-infection can occur. Please see Figure 6.4 for further details.

The stool will be assessed for STH on site on the day of collection by the Kato-Katz method. In addition, preservative will be added to remaining sample to allow the samples to be stored by the study team to allow batch testing with a multiplex PCR including strongyloides, at a later stage in Australia. Comparison of the two diagnostic methods for STH will be possible.

Figure 6.4: Soil Transmitted Helminths (STH) Sampling

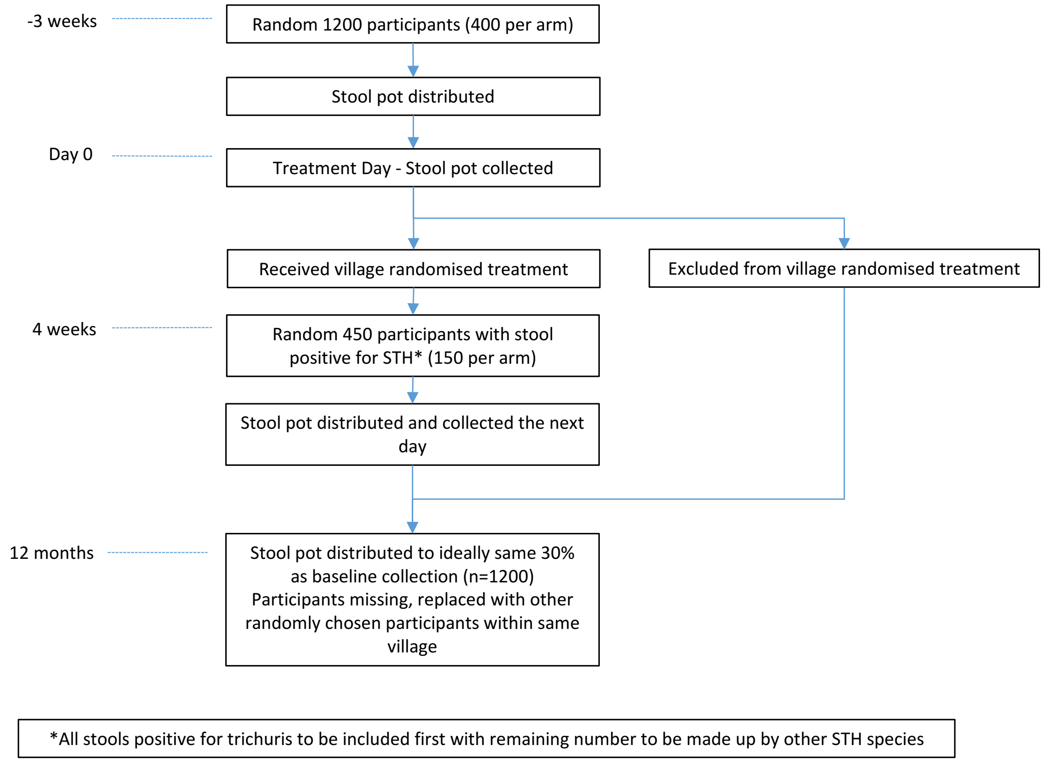

6.5 Clinic Records Review

A nurse will be trained to review records in the local clinic for presentations of skin sores, boils and skin abscesses, scabies, and acute rheumatic fever and rheumatic heart disease according to set case definitions. The two periods to compare will be the 12 months prior to Day 0 and the 12 months following Day 0. This will be undertaken in both Rotuma and Gau. The purpose will be to document any change in number of presentations of scabies and its known secondary complications, following MDA for scabies.

6.6 Adverse Event Monitoring

Following treatment on day 0, participants will be followed actively on a daily basis for the next 2 days (days 1 and 2 post treatment) and then passively between days 3 to 7. Please see the figure 6.6 below, as well as Section 7 and 8, and Appendix 3,4,5 and 5A for more details on this process.

Figure 6.6: AE flow Fiji

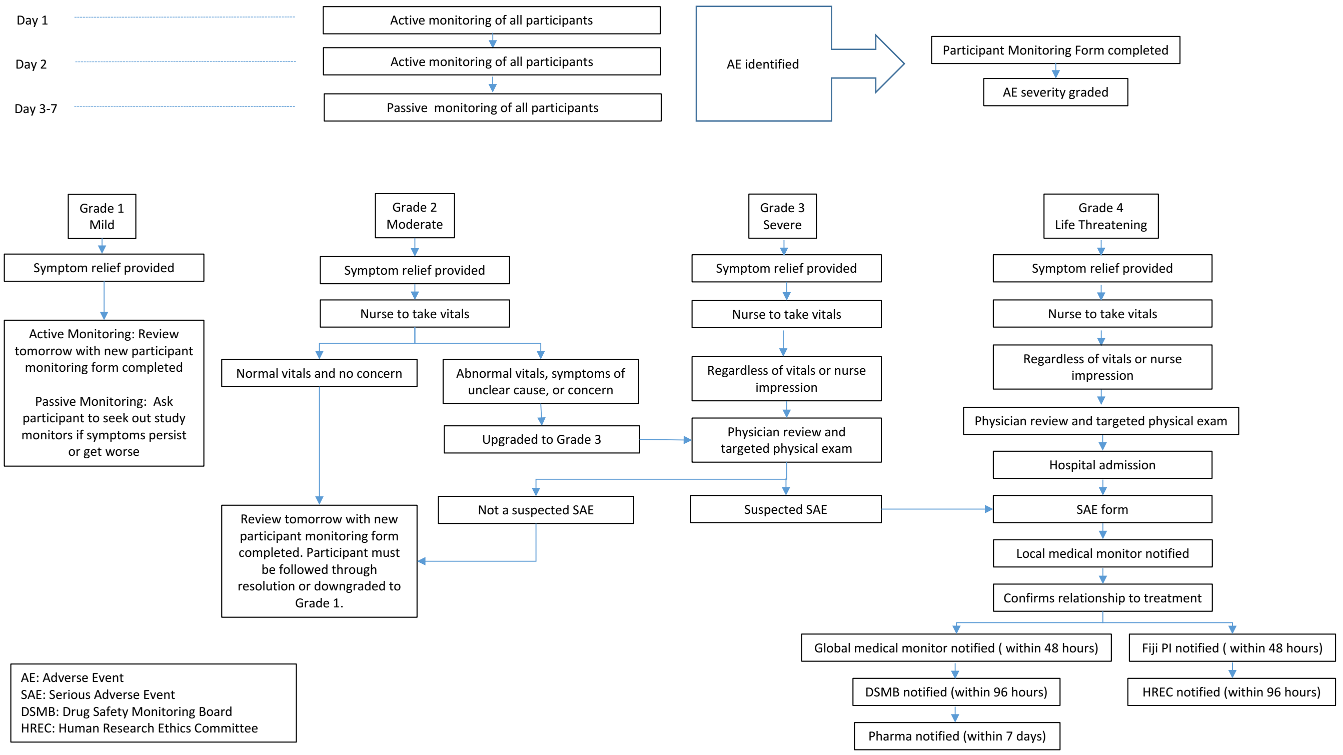

6.7 Overall Study Schedule and Timeline

The study schedule and timeline are illustrated in the figure below. A flow diagram illustrating the study events schedule is presented in 4.2 and Appendix 1.

Community engagement

- Recruitment and training of staff
- Distribution of stool pot and collection instructions to random 30%

Visit 1 "Treatment Day"

Informed Consent
Enrolment with Study ID
Demographic survey
Medical history
Physical examination including weight, skin survey
Stool sample received, Kato-katz, preserved for PCR
Review eligibility for study treatment
LF FTS and DBS
If FTS negative, treatment as per village randomisation
If FTS positive, extra blood for mf before treatment

Visit 2

- AE Active monitoring at 24hrs

Visit 3

- AE Active monitoring at 48hrs

Visit 4-8

- AE Passive monitoring Days 3-7

Visit 9 (only villages randomised to IDA 2 doses IVM)

- Second dose of IVM Day 8

Visit 10

- Acceptability survey of 150 per arm
- Focus Group discussion and informant interviews
- Stool sample from 150 per arm

Visit 11

- Repeat of Visit 1
- Clinic records review

7 SAFETY REPORTING AND SAFETY MONITORING

The post-treatment assessment team will be composed of individuals with basic medical training who are able to perform a medical history and a basic physical examination (physicians, local health workers, nursing and/or medical students). Physicians from the area will be available to assist in the evaluation and management of adverse events.

7.1 Definitions

Adverse Event (AE)

Any untoward medical occurrence in a clinical investigation subject who has received a study product intervention and that does not necessarily have to have a causal relationship with the study product. An AE can, therefore, be any unfavorable and unintended sign (including an abnormal laboratory finding, for example), symptom, or disease temporally associated with the use of a study medicinal product, whether or not considered related to the study medicinal product.

An AE does not include:

- Medical or surgical procedures (e.g. surgery, tooth extraction, transfusion). The condition that leads to the procedure is an adverse event;
- Pre-existing diseases or conditions or laboratory abnormalities present or detected prior to the screening visit that do not worsen.

Serious Adverse Event (SAE)

An SAE is any adverse event that results in any of the following outcomes:

- Death;
- Life-threatening (immediate risk of death);
- Inpatient hospitalization or prolongation of existing hospitalization;
- Persistent or significant disability or incapacity;
- Congenital anomaly/birth defect;
- Important medical events that may not result in death, be life threatening, or require hospitalization may be considered a serious adverse event when, based upon appropriate medical judgment, they may jeopardize the subject and may require medical or surgical intervention to prevent one of the outcomes listed in this definition. Examples of such medical events include allergic bronchospasm requiring intensive treatment in an emergency room or at home, blood dyscrasias or convulsions that do not result in inpatient hospitalization, or the development of drug dependency or drug abuse.

Unexpected

An adverse reaction, the nature or severity of which is not consistent with the applicable product information (e.g., Package Insert).

Expedited Safety Report

Documentation in appropriate form and format summarizing an SAE that meets expedited safety reporting criteria, submitted within the required reporting time frame of applicable regulatory authorities and/or IRBs/IECs of participating countries.

7.2 Assessment of Adverse Events

Active adverse event monitoring will be performed on days 1 and 2 following treatment, Appendix 1. This will be undertaken with an indirect questioning approach in that all participants will be asked if they feel well or unwell. They won't be directed to report or deny any specific symptoms. This is to avoid the potential of symptoms being reported only on the basis of being suggested by the interviewer. Possible adverse events that may occur have been brought to the attention of the participant in the information sheet provided with the consent form.

Any adverse event detected during AE monitoring period will be followed on a daily basis until resolved.

Evaluations will be documented on pre-printed Patient Monitoring forms (Appendix 3) using the scoring instructions for AEs (Appendix 4) or entered directly into an electronic form using tablet computers.

Most adverse events after mass drug administration are associated with killing of mf and are seen in the first 12-24h following treatment. However, occasional adverse events related to adult worm death may be delayed by several days.

To capture these adverse events and to assure that any systemic adverse events that occurred earlier have resolved, study personnel will also visit study villages daily on days 3 through 7 after treatment (passive AE monitoring). Individuals with AEs that interfere with activities of daily living (grade 2 or higher) will have more detailed assessments that will include a brief physical examination (including measurement of temperature, blood pressure and pulse).

Serious Adverse Event (SAE) Assessment and Management

Study participants with a definite or suspected serious AEs (see 7.1 above for details) will be referred to a physician for evaluation. These evaluations will be documented on the SAE form (Appendix 5) following the instructions (Appendix 5A).

7.3 Reporting of Pregnancy

Pregnancy is an exclusion criteria for this study. Although not AEs, pregnancies are reportable events. The pregnancy outcome (e.g., any premature terminations, elective or therapeutic, and any spontaneous abortions or stillbirths, as well as the health status of the mother and child including date of delivery and infant's gender and weight) should be reported. Any pregnant woman inadvertently dosed who has a miscarriage or spontaneous abortion within the week of follow-up will be reported as an SAE.

7.4 Safety Monitoring by the Oversight Committee

A Data Safety Monitoring Board consisting of 4 experts (including 3 physicians) knowledgeable in neglected tropical diseases will be in place to monitor the safety data per country and across countries participating in the DOLF project.

7.5 Country Specific Safety Reporting

The physician reviewing the participant makes the decision if there is a definite or suspected SAE. They will immediately notify the local medical monitor. The local medical monitor must notify within 48 hours the PI in Fiji and the global medical monitor. The PI/investigator must notify within 48 hours of them becoming aware of the SAE to the HREC. Similarly, the global medical monitor must notify the DSMB within 48 hours of them becoming aware. The Global medical monitor must notify the Pharma companies within 7 days.

8 CLINICAL MANAGEMENT OF EVENTS

Individuals who have basic medical training (physicians, nurses and/or nursing or medical students) are who are able to complete and pass a training course will be responsible for the initial adverse event evaluations.

8.1 Adverse Event Monitoring and Management

Adverse event monitoring and management will follow or exceed WHO guidelines. Participants will be visited on the two days following treatment by study personnel with medical training. Formal assessment of adverse events (with a standard form) will take place on days 1 and 2 and later if symptoms persist or start late.

Study personnel will use the toxicity table (Appendix 4) to score adverse events for severity. All grade 2 and above adverse events will be followed until settles to a grade 1 symptom or resolves completely. Study personnel will visit each study area daily for 7 days following MDA treatment to manage any adverse events as detailed in Figure 6.6 and paragraphs 8.1.1-8.1.3 below.

Mild Localized Symptoms

In the case of mild symptomatic reactions local health workers/study personnel will provide antipyretics/analgesics and anti-allergic agents at the time of follow-up. It is anticipated that the majority of adverse events will resolve within a day or two and will not require treatment.

Moderate Adverse Events

In the initial adverse event monitoring if any grade 2 symptom is reported they will be reviewed by a nurse. If the vital signs are outside the normal range, the symptoms are of unclear aetiology or the nurse is unsure they will be referred to a physician for review.

Severe Adverse Events

All grade 3 symptoms and above referred to a physician for review with an Adverse Event and Evaluation and Report form completed. The physician will provide any required immediate treatment and facilitate admission into the hospital or health center as deemed appropriate. If suspected to be a SAE, the physician will complete an SAE form and notify the local medical monitor.

8.2 Rapid Response Teams for Management of Adverse Events

Medical teams will be located at strategic places close to the study sites. Participants, and persons involved in the study (inclusion process and AE monitoring) will be informed about the location and phone numbers of these teams so that they can report directly to these teams if necessary. These teams will be in position from the day of drug administration until the completion of operations. Participants will receive a card and bracelet that have their unique study identifying barcode. These barcodes will be used to look up their treatment received and progress within the electronic database, therefore assisting in their ongoing care. The card will also have study emergency contact details (see a copy of the card below).

Participant Identification Card:

Fiji Integrated Therapy<br>LF, Scabies and STH	Name
Show this card when you go to the health centre or nursing station, if you become ill within 7 days of ____________________	
	Age
Clinic to notify Study Team of your arrival	Gender
Study Emergency Contact - Myra<br>7166748 (Digicel)<br>8011098 (Vodafone)<br>3560418 (TFL)	House No

Barcode Label

9 STATISTICAL CONSIDERATIONS

All subjects that sign the informed consent will be included in the analysis. Subset analyses for safety and efficacy for LF will be undertaken for those that received the study drugs according to their prescribed randomized treatment arm. All participants regardless of treatment will be included in the community prevalence of scabies, impetigo and STH analyses.

Safety

The WHO requires a total of 10,000 participants to detect a SAE rate of 0.1% for each of the treatment regimens. The Fiji site will contribute a sample size of approximately 1300 to the DA regimen and 2600 to the IDA therapy. To obtain the overall sample size required there will be concurrent recruitment in other countries (e.g., India, Indonesia, Papua New Guinea, and Haiti). It is well known that systemic AEs are related to killing of mf and that the severity of AEs is related to mf counts. Since mf rates in the study area are presumed to be relatively low, the Fiji study alone will not be powered to compare rates of SAEs between MDA regimens.

The analysis of the safety studies will be the rates of SAEs as well as the total AEs that occur in infected and in uninfected subjects within the first 7 days post MDA.

Efficacy

Assuming an mf-prevalence of 1% in the study population at baseline, the survey is expected to detect at least 13 mf positive participants in each arm. All participants will be retested for Ag at 12 months, with mf testing if their FTS is positive. This sample size is adequate to demonstrate superiority of the IDA regimen (assumptions: 90% reduction in mf prevalence after IDA and 60% reduction after DA, 80% power for detecting an effect size of 30%). The analysis for efficacy will be complete clearance of mf 12 months post MDA. Clearance of filarial antigenemia at 12 months will be a secondary endpoint for the efficacy analysis.

For the secondary objective of impact on scabies prevalence we have assumed a baseline prevalence of 30% in all three arms. With an estimated absolute reduction of 15 % in the DA arm, 22% in the IDA arm with one dose of ivermectin, and 29% in the IDA arm with two doses of ivermectin, the sample sizes of 1300 in each arm should provide power above 80% for the intended comparisons, taking correction for inflation of type I error (global rate: 0.05 two-sided) into account.

10 DATA HANDLING/RECORD KEEPING/SOURCE DOCUMENTS

Data will be collected using a tablet based system, pre-loaded with study templates. Field teams will be trained in the use of the instruments and data will be uploaded as entries are completed. There will be printed paper forms available as back up to documentation if there are technical difficulties.

10.1 Types of Data Collected

Enrollment Data will include (Appendix 2):

- Site Identification
- Participant Identifier
- Informed Consent Date
- Demographic Information
- Pregnancy/last menstrual period/urine pregnancy test/Breastfeeding and delivery history
- Medical History
- Presence of hydrocele and lymphedema
- History of prior MDA treatment
- Pre-treatment adverse event assessment
- Limited Physical Exam

Laboratory Results

- FTS (filarial antigen test)
- FTS score
- DBS serology for LF, scabies, STH
- mf slide (including mf count)
- STH Kato-katz and PCR diagnostics

Participant Monitoring Forms (24 & 48 hour post treatment) (Appendix 3):

- Adverse Event Assessment
- Physical Examination, as appropriate

Serious Adverse Event (SAE) Form (Appendix 5):

- Participant Identification
- MDA Treatment
- Concomitant Medication taken at the time of the MDA
- AE Description
- Start and Stop Date
- Outcome
- SAE Evaluation and causality to MDA (definite, probable, possible, or unrelated)

10.2 Study Records Retention

Study documents will be retained until the 25th birthday of the youngest participant, in accordance with the requirements of the Australian Therapeutic Goods Administration and Health Privacy Principals. No record will be destroyed in the first 3 years without the written consent of DOLF.

Each participating site will maintain appropriate medical and research records for this trial, in compliance with ICH E6, Section 4.9, regulatory and institutional requirements for the protection

of confidentiality of subjects. Each site participating in this study will permit authorized representatives of the sponsor and regulatory agencies to examine (and when required by applicable law, copy) clinical records for the purposes of clinical site monitoring, quality assurance reviews, audits, and evaluation of the study safety and progress.

10.3 Source Documents

This study will use both paper and electronic source and this may vary by location due to local availability. All sites will be provided with hard copy data collection forms derived from the eCRFs. If data is first entered on paper the study staff will enter the data into the electronic capture system.

11 RESPONSIBILITIES

11.1 Investigator Responsibilities

Good Clinical Practice

The investigator will ensure that the basic principles of Good Clinical Practice are followed along with the appropriate laws and regulations of the country in which the research is conducted.

11.2 Institutional Review Board (IRB)/Ethics Committee (EC)

The protocol and any accompanying material to be provided to the participants such as the informed consent will be submitted to the EC for review and approval. Approval from the committee must be obtained before starting the study and should be documented in correspondence to the investigator.

Any modifications to the protocol after receipt of the IRB or EC approval must be submitted to the committee for approval prior to implementation.

11.3 Informed Consent

It is the responsibility of the investigator to obtain written informed consent from each individual (or their guardian) participating in the study after adequate explanation of the aims, methods, objectives and potential risk of any study related procedures. The investigator must use an IRB/EC approved Participant Informed Consent Form (Appendix 6) for all participants 18 years and older. If the participant is below 18 years of age, or is an adult but doesn't have capacity to consent a Parent/Guardian Informed Consent Form (Appendix 6A) must be used. In addition, if the participant is 13 years and older and less than 18 years and is deemed capable to give consent, a signed Participant form will be required in addition to the Parent/Guardian Form. The investigators will accept either signed (cursive) or printed signatures or a witnessed mark in the case of illiterate study participants on the consent form.

Only the principal investigators or study staff authorized and certified to obtain consent will consent subjects for this study. Only individuals who have signed the consent form will be enrolled in the study.

Entry into the study and participation will be strictly voluntary. It will be made clear that refusal to participate or a decision to withdraw can occur at any time throughout the course of the study and will not influence their rights or the care they receive at local health facilities. Potential participants will be told that all of their health information will be confidential and that records will be coded without personal identifiers before they are shared with statisticians or project scientists outside of the village/region/country. They will also be told that no monetary or other gains are offered in exchange for participation.

Informed Consent Training

The study team will conduct on-site training sessions for study personnel who will be collecting study information, specimens, and obtaining consent from participants in the study. Each step of the study will be explained in detail to the local study personnel. The basic principles of informed consent process, documentation of informed consent, protection of subjects' rights, confidentiality, and handling of data will be covered in these training sessions. Study personnel will be monitored by the on-site project manager on a regular basis to ensure compliance with the

principles of informed consent. The investigators and study personnel who will obtain consent from study participants will also receive training in human subject regulations and good clinical practices (GCP).

11.4 Participant Privacy

Privacy of the study participants will be maintained by assigning study participants a unique study identification number (UNID). All data, blood samples and laboratory results will be recorded and analyzed by UNID with no personal identifiers. All information collected, including demographic information about enrolled subjects will be kept confidential and available only to the investigators and authorized study personnel such as the data manager.

Though most data will be collected on tablets, all written forms (i.e., consent and data collection forms paper forms are used) will be stored in a designated locked area. All forms will be labelled and filed in cabinets with the study protocol number, PI's names and collection dates. These cabinets will be metal and have functioning locks. Keys will be kept with the Project Manager. All electronic devices on which data are entered will be password protected. PIs and/or Project Manager will authorize access. The paper forms will be stored until the 25th birthday of the youngest participant.

11.5 Data Ownership

The data are the property of MCRI and Fiji Ministry of Health. The Principal Investigators, Co-investigators and key personnel may use the results of this study for publications, presentations at scientific meetings or as preliminary data for subsequent grant applications. Confidentiality of study participants will be maintained by not using names or personal identifiers. AFC and UR will provide de-identified data from the study to DOLF for use in publications and presentations that present results across different study sites. At least one Fiji specific researcher will be included as an author for any publications with data from Fiji.

The study site Project Coordinator will permit access to all documents and records that may require inspection by the funding agencies, governmental regulatory agencies, institutional review boards or its authorized representatives.

12 PUBLICATION POLICY

Manuscripts should be submitted for publication no later than one year following the date of the “last patient/last visit” at the 12 months review. This study includes follow-up data collection past the primary end point, including acceptability and efficacy results. It is not necessary to wait for the follow-up studies to be completed in order to publish the primary safety data.

Endemic country investigators have an obligation to publish the results of DOLF studies conducted in their country. These results benefit the national NTD programs and the citizens of the country where the study was completed. DOLF collaborating institutions are willing to help their endemic country partners with the data analysis, manuscript preparation, publication fees, etc. However, the lead author should be an investigator from the country where the study was performed.

DOLF scientists will be responsible for publishing the results from the aggregated data that combines the results from multiple study sites. The purpose of these manuscripts is to consider the similarities and differences in results obtained in different countries. These publications will not include as much detailed data or analyses as the country specific publications. Publications that report multi-country results will have at least one co-author from each country included in the manuscript.

13 AMENDMENT – EXTENSION 24 MONTH FOLLOW UP

13.1 Rationale

Our preliminary results of clearance of microfilaremia in infected individuals at 12 months is less than expected in Rotuma compared to what has been reported from the other four sites. To better understand the response of LF positive individuals to treatment with either DA, IDA, or a combination of both over 2 years, we aim to recruit 131 participants in Rotuma who were mf positive at baseline and/or 12 month review and reassess them for LF disease.

13.2 Objectives

1. To compare the efficacy of IDA (3 drug therapy) to DA (2 drug therapy) for clearance of mf and filarial antigenemia (Ag) at 12 months on Rotuma.
2. To compare the efficacy of two rounds of annual IDA to one round of DA and one round of IDA for clearance of mf and filarial antigenemia at 24 months on Rotuma.
3. To understand and compare features of circulating LF parasites between different treatment groups on Rotuma.
4. To compare acceptability of IDA MDA to DA MDA in individuals positive for LF disease on Rotuma.

13.3 Enrolment

Eligible participants will be any participant from Rotuma who at baseline and/or 12 months was positive for circulating microfilaremia on finger-prick blood smear. Participants will remain eligible if only enrolled at one previous time point. There were 106 participants with microfilaremia at baseline. There were an additional 27 participants with microfilaremia at 12 month review. We know of 2 participants who are deceased, leaving eligible cohort of 131. The youngest positive participant will be 5 years of age in 2019.

Eligible individuals will be approached and asked to provide written consent (as described in section 4.6). Information statements and consent forms are based on previously approved (Appendix 6 and 6A) and are attached as separate documents. Loss to follow up will be recorded with a reason: declined, permanently left island, temporarily away or deceased.

Participants will be re-identified by their unique study barcode and results linked to their previously collected data at baseline and/or 12 month review, including infection intensity and treatment received.

Participants data will be grouped according to how many months following first enrolment with the study eg the results from a participant first enrolled at 12 month timepoint, will be have their results from this extension study grouped in the 12 month analysis and not the 24 month cohort.

13.4 Study procedures

Figure 13.4 outlines the procedures for the 24 month review.

Blood tests

To allow the possibility of testing circulating microfilariae for drug resistance and parasite genomics, we will collect 6ml of blood. This volume requires venepuncture rather than finger-prick collection. Venous blood is suitable and will give equivalent results for FTS and DBS samples. Venous blood will be ***tested*** using the FTS (0.1ml) and membrane filtration (2ml), with the remaining ***stored*** (0.1 ml as DBS an 3.8ml as plasma/cells).

If the FTS is positive, participants will have a finger-prick blood collection of approximately 60µl for a three line blood smear. Microfilariae have a higher concentration in the capillaries compared to venous blood and therefore it is necessary to use finger-prick blood for this low volume method. It will also ensure that mf results are compatible and can be directly compared with results from baseline and 12 months.

"Membrane filtration" will be used to test response of microfilariae to treatment. This requires 1ml of venous blood per filtration and is performed in duplicate (2ml total). Due to the increase blood volume used compared to mf smears, the sensitivity of this method to detect low density microfilaremia is increased.

Any remaining venous blood (approximately 3.8ml) will be centrifuged, with plasma and cells/pellet with microfilariae, stored separately and frozen at -20 degrees. This will be shipped to co-investigators at Washington University for further in-depth laboratory analyses, that may include antigen/antibody response, drug resistance testing and parasite genomics. These tests will be required if our membrane filtration results show that participants from Rotuma are not clearing microfilariae after treatment as expected from other studies. If clearance has been adequate, the blood will be reserved for development of new tests (likely novel antigen/antibody markers) to monitor treatment success that can be adopted by filariasis programs in monitoring program response and thresholds for cessation of MDA.

If a participant declines venous blood sampling or if there are difficulties with venous blood collection, they will be offered finger-prick blood sampling for FTS, DBS and mf smears as previously described in section 4.7.

Acceptability assessment

Short focused acceptability surveys will be conducted with participants who were microfilaremic at either timepoint. We will use the same series of questions as used in the 12 month acceptability survey. This includes epidemiology questions exploring potential reasons for increased risk of transmission, as well as seeking the view of individuals on acceptance of treatment and barriers to future engagement in filariasis MDA.

Figure 13.4 Procedures for 24 m review

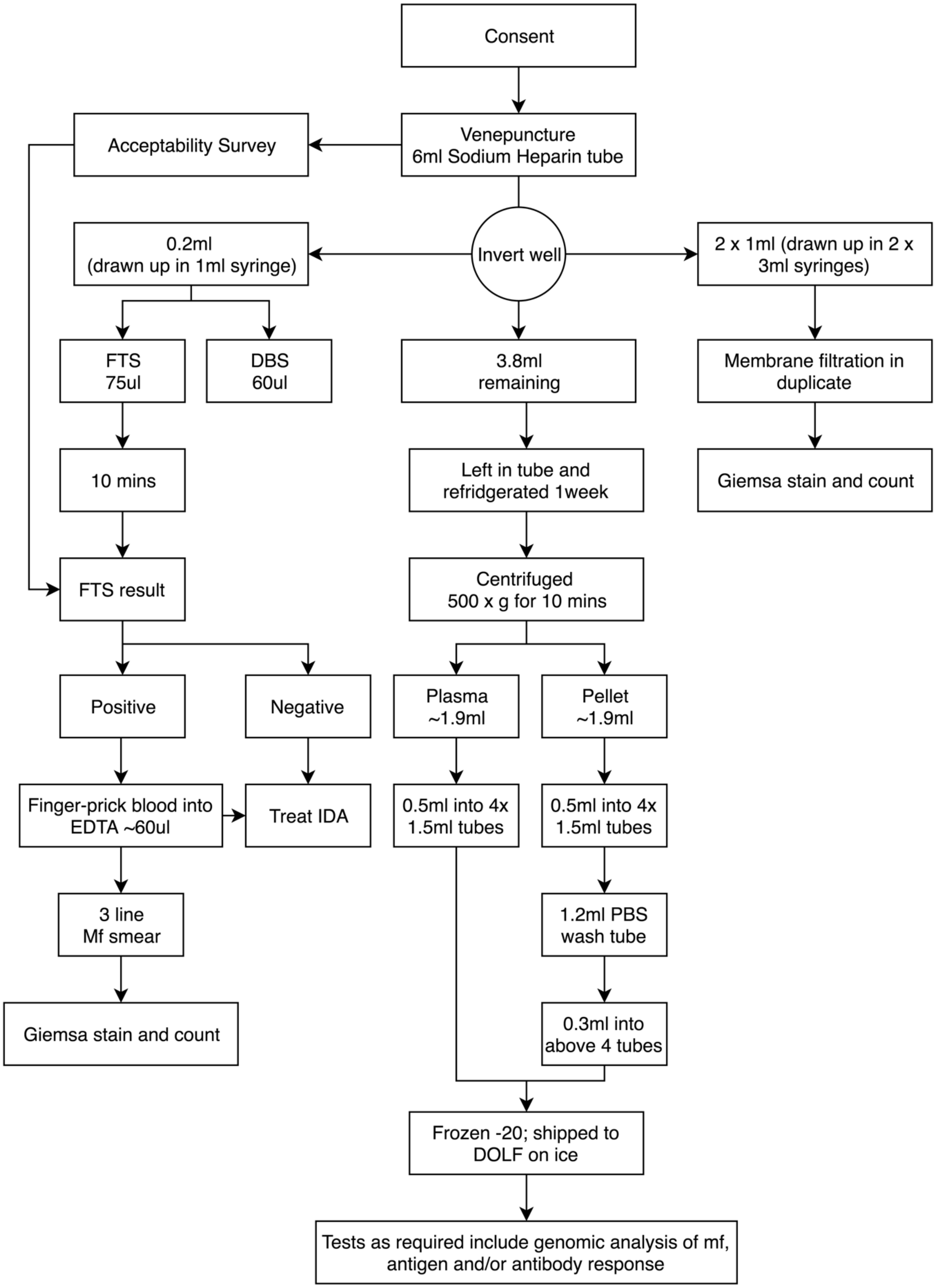

13.5 Treatment

Following LF assessments all participants will be offered LF treatment regardless of LF test results (unless ineligible: severe illness, pregnant, breastfeeding within 7 days of delivery, allergy to IDA and/or less than 15kg). IDA is the current recommended treatment for LF in Fiji and will be the preferred combination based on availability. WHO-donated medications will be used and sourced through the National LF Program in Fiji. Participants will be advised to seek review with their local nurse/doctor if feeling unwell after treatment.

14 LITERATURE REFERENCES

1. Thomsen EK, Sanuku N, Baea M, Satofan S, Maki E, Lombore B, et al. Efficacy, Safety, and Pharmacokinetics of Coadministered Diethylcarbamazine, Albendazole, and Ivermectin for Treatment of Bancroftian Filariasis. Clin Infect Dis. 2016;62(3):334-41.
2. Ichimori K. MDA-Lymphatic Filariasis. Trop Med Health. 2014;42(2 Suppl):21-4.
3. Romani L, Koroivueta J, Steer AC, Kama M, Kaldor JM, Wand H, et al. Scabies and impetigo prevalence and risk factors in Fiji: a national survey. PLoS Neglected Tropical Diseases [electronic resource]. 2015;9(3):e0003452.
4. Steer AC, Jenney AJ, Oppedisano F, Batzloff MR, Hartas J, Passmore J, et al. High burden of invasive beta-haemolytic streptococcal infections in Fiji. Epidemiol Infect. 2008;136(5):621-7.
5. Jenney A, Holt D, Ritika R, Southwell P, Pravin S, Buadromo E, et al. The clinical and molecular epidemiology of Staphylococcus aureus infections in Fiji. BMC Infect Dis. 2014;14:160.
6. Carapetis JR, Walker AM, Hibble M, Sriprakash KS, Currie BJ. Clinical and epidemiological features of group A streptococcal bacteraemia in a region with hyperendemic superficial streptococcal infection. Epidemiol Infect. 1999;122(1):59-65.
7. Carapetis JR, Steer AC, Mulholland EK, Weber M. The global burden of group A streptococcal diseases. Lancet Infect Dis. 2005;5(11):685-94.
8. Romani L, Whitfeld MJ, Koroivueta J, Kama M, Wand H, Tikoduadua L, et al. Mass Drug Administration for Scabies Control in a Population with Endemic Disease. N Engl J Med. 2015;373(24):2305-13.
9. Mohammed KA, Deb RM, Stanton MC, Molyneux DH. Soil transmitted helminths and scabies in Zanzibar, Tanzania following mass drug administration for lymphatic filariasis--a rapid assessment methodology to assess impact. Parasit Vectors. 2012;5:299.
10. Thomas M, Woodfield G, Moses C, Amos G. Soil-transmitted helminth infection, skin infection, anaemia, and growth retardation in schoolchildren of Taveuni Island, Fiji. N Z Med J. 2005;118(1216):U1492.
11. PERRIGO A, MI 49010. Permethrin Cream, 5% Treatment Only. Medication Leaflet.
12. Horton J. Albendazole: a review of anthelmintic efficacy and safety in humans. Parasitology. 2000;121 Suppl:S113-32.
13. Goa KL, McTavish D, Clissold SP. Ivermectin. A review of its antifilarial activity, pharmacokinetic properties and clinical efficacy in onchocerciasis. Drugs. 1991;42(4):640-58.
14. Edwards G. Ivermectin: does P-glycoprotein play a role in neurotoxicity? Filaria Journal. 2003;2(Suppl 1):S8-S.
15. Ottesen EA, Campbell WC. Ivermectin in human medicine. J Antimicrob Chemother. 1994;34(2):195-203.
16. Awadzi K, Edwards G, Duke BO, Opoku NO, Attah SK, Addy ET, et al. The co-administration of ivermectin and albendazole--safety, pharmacokinetics and efficacy against Onchocerca volvulus. Ann Trop Med Parasitol. 2003;97(2):165-78.
17. Steer AC, Tikoduadua LV, Manalac EM, Colquhoun S, Carapetis JR, Maclennan C. Validation of an Integrated Management of Childhood Illness algorithm for managing common skin conditions in Fiji. Bull World Health Organ. 2009;87(3):173-9.

18. Krentel A, Fischer PU, Weil GJ. A Review of Factors That Influence Individual Compliance with Mass Drug Administration for Elimination of Lymphatic Filariasis. PLoS neglected tropical diseases. 2013;7(11):e2447.
19. Babu BV, Babu GR. Coverage of, and compliance with, mass drug administration under the programme to eliminate lymphatic filariasis in India: a systematic review. Transactions of the Royal Society of Tropical Medicine and Hygiene. 2014;108(9):538-49.
20. Thomsen E, Sanuku N, Baea M, Satofan S, Maki E, Lombore B, et al. Efficacy, Safety, and Pharmacokinetics of Coadministered Diethylcarbamazine, Albendazole, and Ivermectin for Treatment of Bancroftian Filariasis. Clinical Infectious Diseases. 2016;62(3):334-41.
21. Ramaiah KD, Kumar KNV, Hosein E, Krishnamoorthy P, Augustin DJ, Snehalatha KS, et al. A campaign of 'communication for behavioural impact' to improve mass drug administrations against lymphatic filariasis: structure, implementation and impact on people's knowledge and treatment coverage. Annals of Tropical Medicine and Parasitology. 2006;100(4):345-61.
22. Babu BV, Suchismita M. Mass drug administration under the programme to eliminate lymphatic filariasis in Orissa, India: a mixed-methods study to identify factors associated with compliance and non-compliance. Transactions of the Royal Society of Tropical Medicine and Hygiene. 2008;102(12):1207-13.
23. Bockarie MJ, Tisch DJ, Kastens W, Alexander NDE, Dimber Z, Bockarie F, et al. Mass treatment to eliminate filariasis in Papua New Guinea. New England Journal of Medicine. 2002;347(23):1841-8.
24. Krentel A, Fischer P, Manoempil P, Supali T, Servais G, Rückert P. Using knowledge, attitudes and practice [KAP] surveys on lymphatic filariasis to prepare a health promotion campaign for mass drug administration in Alor District, Indonesia. Tropical Medicine & International Health. 2006;11(11):1731-40.
25. Cantey PT, Rao G, Rout J, Fox LM. Predictors of compliance with a mass drug administration programme for lymphatic filariasis in Orissa State, India 2008. Tropical Medicine and International Health. 2010;15(2):224-31.
26. Mathieu E, Lammie PJ, Radday J, Beach MJ, Streit T, Wendt J, et al. Factors associated with participation in a campaign of mass treatment against lymphatic filariasis, in Leogane, Haiti. Annals of Tropical Medicine and Parasitology. 2004;98(7):703-14.
27. Krentel A, Aunger R. Causal chain mapping: a novel method to analyse treatment compliance decisions relating to lymphatic filariasis elimination in Alor, Indonesia. Health Policy Plan. 2011.
28. Fraser M, Taleo G, Taleo F, Yaviong J, Amos M, Babu M, et al. Evaluation of the program to eliminate lymphatic filariasis in Vanuatu following two years of mass drug administration implementation: results and methodologic approach. The American journal of tropical medicine and hygiene. 2005;73(4):753-8.
29. Nandha B, Sadanandane C, Jambulingam P, Das P. Delivery strategy of mass annual single dose DEC administration to eliminate lymphatic filariasis in the urban areas of Pondicherry, South India: 5 years of experience. Filaria J. 2007;6:7.
30. Worrell C, Mathieu E. Drug Coverage Surveys for Neglected Tropical Diseases: 10 Years of Field Experience. American Journal of Tropical Medicine and Hygiene. 2012;87(2):216-22.
31. Witt C, Ottesen EA. Lymphatic filariasis: an infection of childhood. Tropical Medicine & International Health. 2001;6(8):582-606.

15 LIST OF APPENDICES

Appendix 1: Study Drug Flow Diagram - Fiji

Appendix 2: Participant Enrollment Form [Example conversion to electronic database]

Appendix 3: Participant Monitoring Form of Adverse Events Form [Example conversion to electronic database]

Appendix 4: Guide to Assigning Adverse Event Severity

Appendix 5: Serious Adverse Event (SAE) Form [Example]

Appendix 5A: Required Reporting & Guidelines for SAE(s)

Appendix 6: Participant Information Statement and Consent [Example]

Appendix 6A: Parent Information Statement and Consent [Example]

Appendix 7: Treatment Acceptability Study Protocol

APPENDIX 1: STUDY FLOW DIAGRAM - FIJI

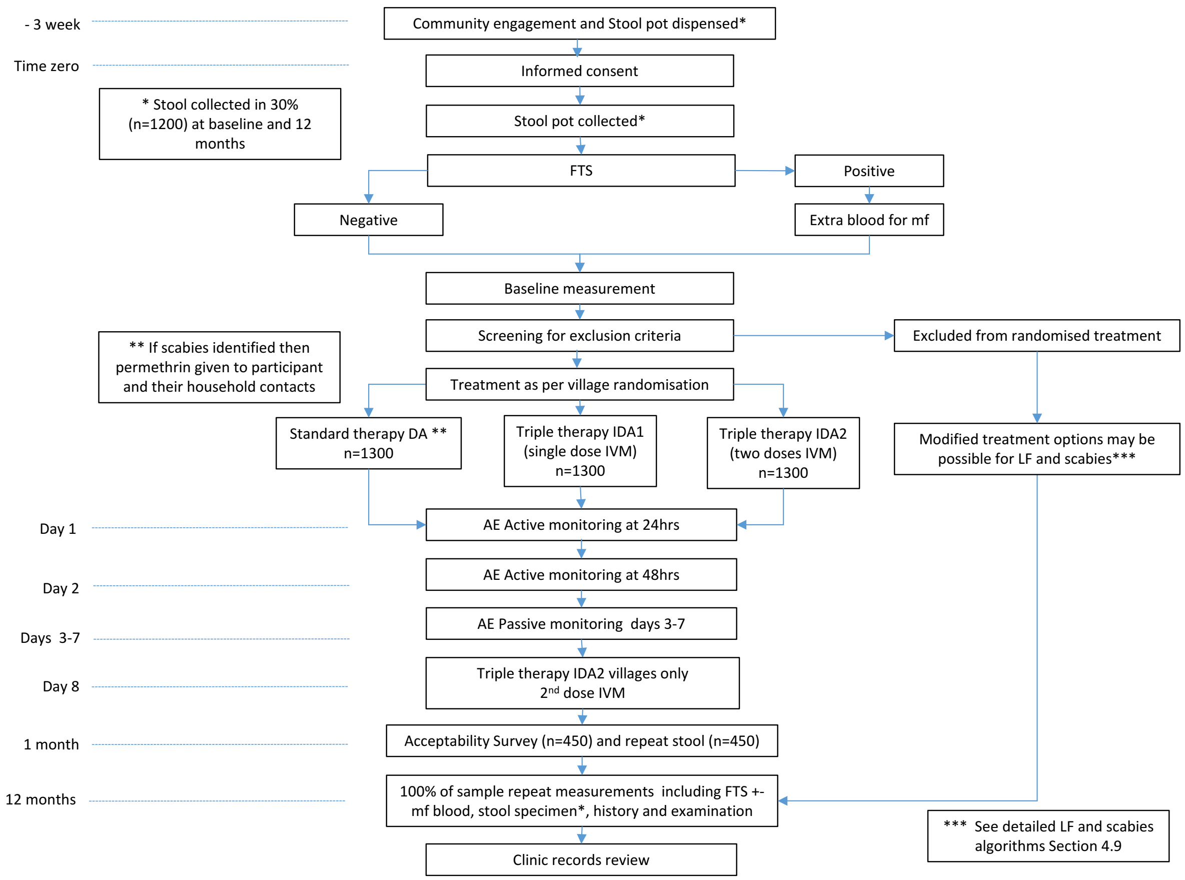

FTS: Filariasis Test Strip; Dried Blood Spot (DBS) also taken at this time
mf: microfilariae
DA: Diethylcarbamazine (DEC) and Albendazole (ALB)
IDA: Ivermectin (IVM), DEC and ALB
MDA: mass drug administration
AE: Adverse event
KAP: Knowledge, Attitudes and Practices

APPENDIX 2: PARTICIPANT DATA COLLECTION FORM [EXAMPLE CONVERSION TO ELECTRONIC DATABASE]

**DOLF IDA Safety Study Barcode:**	

1. Participant Monitoring:

**Is Participant New?**	☐ Yes ☐ No
**Was Participant found?**	☐ Yes ☐ No
**If not, why?**	☐Temporarily away<br>☐Permanently away<br>☐Deceased<br>☐Declined<br>☐Not home<br>☐Other
**If Other, please describe:**	

2. Demographics

<table>
<tr><td colspan="4">Informed Consent Date (required) (DD-MM-YYYY): ______-______-______</td></tr>
<tr><td colspan="2">Island: ☐ Gau</td><td colspan="2">☐Rotuma</td></tr>
<tr><td>Village (required)

☐ Lamiti
☐ Lekanai
☐ Levuka-i-Gau
☐ Lovu
☐ Malawai
☐ Nacavanadi
☐ Nadrodro
☐ Navukailagi
☐ Nawaikama
☐ Nukuloa
☐ Qarani</td><td>☐ Sawaieke
☐ Somosomo
☐ Vadravadra
☐ Vanuaso
☐ Vione
☐ Yadua
☐ Gau Secondary School
☐ Gau_1</td><td>Village (required)

☐ Ahau
☐ Fapufa
☐ Hapmak
☐ Itumuta
☐ Juju
☐ Kalvaka
☐ Lau/Feavai
☐ Lopta
☐ Losa</td><td>☐ Malha'a
☐ Motusa
☐ Noatau
☐ Oinafa
☐ Paptea
☐ Pepjei
☐ Savlei
☐ Tuakoi
☐ Rotuma_1</td></tr>
</table>

Gender:<br>☐M ☐F	*Note: if exact date is not known, list as January 01 with known birth year*			Age (Years):
	Birth Day (DD):	Birth Month (MM):	Birth Year (YYYY):	
Weight (in kg):				

3.

Section Header	Field Label	Choices, Calculations, OR Slider Labels	Branching Logic (Show field only if...)	Comments
FTS TEST RESULTS				
	FTS (filarial antigen test) results	1, Positive<br>2, Negative<br>3, Undetermined		If FTS is positive, we will need the form to popup – need mf smear<br><br>If Undetermined, repeat the teast – if 2nd test is undetermined, then treat this as positive<br><br>If negative, continue form
	FTS score	1, 1 – Weak<br>2, 2 – Medium<br>3, 3 - Strong<br>4, Not Done	If FTS positive…	

MF Smear Blood Taken?	Filter Paper Bleed?
☐Yes ☐Declined<br>☐No	☐Yes ☐Declined<br>☐No

4. Epidemiological Questions

Self-reported lymphedema: ☐Yes ☐No	
Lymphedema (grade 1-4):	☐ No lymphedema<br>☐ Grade 1

	☐ Grade 2<br>☐ Grade 3<br>☐ Grade 4
**Self-reported hydrocele (men only)?**	☐Yes ☐No
**Self-reported skin rash?**	☐Yes ☐No
**Does itching interfere with your sleep?**	☐Yes ☐No
**Have you left the island in the last 12 months? (Fiji Only)**	☐Yes ☐No
**Have you moved villages in the last 12 months? (Fiji Only)**	☐Yes ☐No
**If Yes, list other villages resided for one month or more. (Fiji Only)**	
**Did you use a bednet last night?**	☐Yes ☐ No
**Do you use screens on the windows, mosquito coils, or indoor spraying?**	☐Yes ☐No
**Do you mainly use water from: (Choose one)**	☐ public pump/well<br>☐ river/creek<br>☐ water tank<br>☐ public pipe
**Does your family have access to a private latrine or toilet?**	☐Yes ☐No

5. Acceptability Questions (14 years and older)

**NOT Including this year, how many times have you taken pills for LF?**	1. ☐ Never<br>2. ☐ One time<br>3. ☐ 2 or more times
**Last year, there was a special distribution of LF treatment to some households in your area. Did you receive treatment during this special distribution of LF treatment?**	4.<br>5. ☐ Yes<br>6. ☐ No

**If no, why not?**	7. ☐ Was not eligible to participate<br>8. ☐ Was away at the time of distribution<br>9. ☐ No/poor information provided<br>10. ☐ No/low risk of LF disease<br>11. ☐ No/low confidence or trust<br>12. ☐ Afraid<br>13. ☐ Other
**If other, please describe:**	14.<br>15.<br>16.<br>17.<br>18.<br>19.<br>20.<br>21.<br>22.<br>23.<br>24.<br>25.
**Do you think you should take these pills even if you don't feel sick?**	26. ☐ Yes<br>27. ☐ No<br>28. ☐ Don’t know
**How many people in your village do you think have LF?**	29. ☐ None<br>30. ☐ Some<br>31. ☐ Many<br>32. ☐ Don’t know

Here are some faces expressing various feelings. Which face comes closest to how you felt about the number of pills you received?

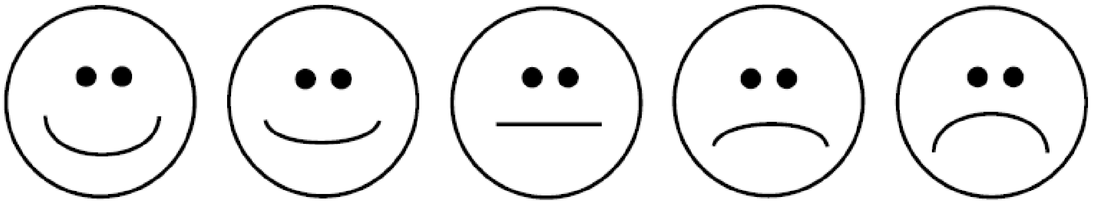

☐ Very Happy ☐ Happy ☐ Neutral ☐ Sad ☐ Very Sad

Please rate your opinion on the following statements:	Disagree a lot	Disagree	Agree	Agree a lot
**These drugs work against LF**				

**These drugs work against itching**				
**These drugs work against intestinal worms**				
**I would take this treatment again**				
**I would recommend this treatment to my relatives**				
**I would be willing to change my family's routine so that we took the treatment again**				
**I liked this treatment**				
**This treatment is a good way to help our health problems here**				
**Overall this treatment will help my community**				

6.

**Section Header**	**Field Label**	**Choices, Calculations, OR Slider Labels**	**Branching Logic (Show field only if...)**	**Comments**
**Special Interest Events - Scabies**				
History - Skin	Do you feel itchy?	0, No<br>1, Yes		PLEASE MAKE THIS ANSWER AUTOMATICALLY YES IF THEY ANSWERED IN "PRE-TREATMENT AE ASSESSMENT" YES TO ITCHY SKIN
	Does anyone else in the house feel itchy?	0, No<br>1, Yes		
Scabies<br><br>Make sure only medical trained personnel fill out.	Can you see scabies lesions?	0, No<br>1, Yes		
	Where are the scabies lesions?	1, Head and neck<br>2, Arms including hands<br>3, Legs including feet<br>4, Torso including armpits and groin	If Scabies lesions Yes	
	How many scabies lesions are there?	1, Mild (10 or less)<br>2, Moderate (11-49)<br>3, Severe (50 or more)	If Scabies lesions Yes	
	Is there any scaling and crusting of the skin consistent with Norwegian crusted scabies?	0, No<br>1, Yes	If Scabies lesions Yes	POPUP: If Yes, the patient must be treated off-study. Please contact the study coordinator.
	Do the scabies lesions look infected - pus filled	0, No<br>1, Yes	If Scabies lesions Yes	

	sores or crusted sores over scabies lesions?			
SKIN SORES	Can you see any skin sores?<br>Include infected scabies	0, No<br>1, Yes		
	Where are the skin sores?	1, Head and neck<br>2, Arms including hands<br>3, Legs including feet<br>4, Torso including armpits and groin	If Skin Sores Yes	
	How many skin sores are there?	1, Mild (10 or less)<br>2, Moderate (11-49)<br>3, Severe (50 or more)	If Skin Sores Yes	
	Do any of the sores look infected - crusted, pus filled or surrounding redness?	0, No<br>1, Yes	If Skin Sores Yes	
	Since there are signs of bacterial infection the person needs referral to the local clinic. Has a referral been made?	0, No<br>1, Yes	If infected scabies or infected sores yes	

7. Pregnancy History

**Have you had your menses in last 4 weeks?**	☐ Yes<br>☐ No<br>☐ Unsure<br>☐ No menarche<br>☐ Known menopause<br>☐ No, pregnant
**Pregnancy Test Results:**<br>☐Positive<br>☐Negative<br>☐Undetermined<br>☐Declined	*If pregnancy test is positive, undetermined or declined, participant cannot receive their allocated LF MDA treatment. They still need permethrin if they or someone in their house has scabies.*

8. Inclusion/Exclusion Criteria

**Inclusion Criteria for MDA:**	
Able to provide informed consent or parental consent to participate in the study (Forms to be attached)	☐No ☐Yes
**Exclusion Criteria for MDA:**	

Less than 2 years of age	☐No ☐Yes
Less than 15kgs in weight	☐No ☐Yes
FEMALE ONLY: Pregnant women (DEC, ivermectin and albendazole are contraindicated in pregnancy)	☐No ☐Yes/Unsure
FEMALE ONLY: Breastfeeding in first 7 days following delivery	☐No ☐Yes
Severe acute or chronic illness (for example: chronic renal failure, inability to care for oneself with activities of daily living) except for features of filarial disease	☐No ☐Yes
History of previous allergy to MDA drugs	☐No ☐Yes
**Additional Exclusion Criteria for Ivermectin:**	
Less than 5 years of age	☐No ☐Yes
**Inclusion Criteria for Permethrin:**	
Identified as having scabies or someone in their house has scabies	☐No ☐Yes
**Exclusion Criteria for Permethrin:**	
History of previous allergy to Permethrin	☐No ☐Yes

Section Header	Field Label	Choices, Calculations, OR Slider Labels	Branching Logic (Show field only if...)	Comments
**9. MDA TREATMENT**				
MDA TREATMENT	Record Participant ID	BARCODE		
	Please calculate dose using Dosage Card according to Weight = [weight]			AUTOPOPULATE BASED ON MDA DRUG DOSING CHART – IF POSSIBLE
	Drug Regimen:	☐ Double drug therapy (ALB, DEC)<br>☐ Triple drug therapy (ALB, DEC, IVM)<br>☐ Permethrin Only<br>☐ Declined		
	MDA Treatment Date:			
	Albendazole (ALB) dose (400 mg)	1, 1 tablet (400 mg)		
	DEC (diethylcarbamazine citrate) dose (6mg/kg)	1, 1 tablet (100 mg)<br>2, 2 tablets (200 mg)<br>3, 3 tablets (300 mg)<br>4, 4 tablets (400 mg)<br>5, 5 tablets (500 mg)<br>6, 6 tablets (600 mg)		
	Ivermectin (IVM) dose (200 ug /kg)	1, 1 tablet (3 mg)<br>2, 2 tablets (6 mg)<br>3, 3 tablets (9 mg)<br>4, 4 tablets (12 mg)<br>5, 5 tablets (15 mg)<br>6, 6 tablets(18 mg)<br>7, 7 tablets(21 mg)	If triple drug therapy is selected	

Permethrin 5% (1 tube)	☐Yes ☐No
Did Patient vomit or spit out the pills?	☐Yes ☐No
Repeated MDA Treatment Date (DD-MM-YYYY):	______-______-______
Repeated Albendazole (ALB) dose (400 mg)	☐ 1 tablet (400 mg) Not done

Repeated Diethylcarbamazine citrate (DEC) dose (6mg/kg)	☐ 1 tablet (100 mg)<br>☐ 2 tablets (200 mg)<br>☐ 3 tablets (300 mg)	☐ 4 tablet (400 mg)<br>☐ 5 tablets (500 mg)<br>☐ 6 tablets (600 mg)
Repeated Ivermectin (IVM) dose (200 ug /kg)	☐ 1 tablet (3 mg)<br>☐ 2 tablets (6 mg)<br>☐ 3 tablets (9 mg)<br>☐ 4 tablets (12 mg)	☐ 5 tablet (15 mg)<br>☐ 6 tablets (18 mg)<br>☐ 7 tablets (21 mg)

10.

Section Header	Field Label	Choices, Calculations, OR Slider Labels	Branching Logic (Show field only if...)	Comments
**MF TEST RESULTS**				
MICROFILAREMIA (MF) TEST RESULTS	Record Participant ID	BARCODE		
	MF (microfilaremia) positive?	0, No<br>1, Yes		
	W. bancrofti MF count:			

11. Soil Transmitted Helminths (STH) Test Results

STH Lab Test Date (DD-MM-YYYY):	______-______-______
**KK1 Reader:**	
KK1 Ascaris egg counts:	
KK1 Hookworm egg counts:	
KK1 Trichuris egg counts:	
KK1 Enterobius egg counts (*Indonesia Only*)	
KK1 Other egg counts:	
KK1 Other egg name:	

**KK2 Reader:**	
KK2 Ascaris egg counts:	
KK2 Hookworm egg counts:	
KK2 Trichuris egg counts:	
KK2 Enterobius egg counts (*Indonesia Only*)	
KK2 Other egg counts:	
KK2 Other egg name:	
Notes:	

APPENDIX 3: PARTICIPANT MONITORING OF ADVERSE EVENTS FORM [EXAMPLE CONVERSION TO ELECTRONIC DATABASE]

FOR: Day 1 & 2, if needed days 3-7

Section Header	Field Label	Choices, Calculations, OR Slider Labels	Branching Logic (Show field only if...)	Comments
**PARTICIPANT MONITORING**				
PARTICIPANT INFORMATION	Record Participant ID	BARCODE		
	Site:	[LOCATION]		Pre-filled AUTOPOPULATE
	Treatment Date	[MDA Treatment Date]		Pre-filled AUTOPOPULATE
	Monitoring Date			
	Participant Found:	0, No<br>1, Yes		Only for Day 1 and 2 Site Visits<br><br>If not found, do not finish modules for that Site Visit Day

Section Header	Field Label	Choices, Calculations, OR Slider Labels	Branching Logic (Show field only if...)	Comments
**ADVERSE EVENTS**				
ADVERSE EVENTS	Were any adverse events experienced?	0, No<br>1, Yes		
Record unique identifier for each adverse event for this subject. Number sequence for all following forms should not duplicate existing numbers for the subject.	AE Identifier		If any AE are reported, all the following questions are shown…	
Table1: Adverse Events of Interest				
Neurologic	Fever	1, 1 - Mild<br>2, 2 – Moderate<br>3, 3 - Severe<br>4, 4 - Life-threatening	If AE yes	Would like to only have the Section Headers are visible and then if selected, then the individual symptoms would be visible.
	Dizziness, lightheaded	1, 1 - Mild<br>2, 2 – Moderate<br>3, 3 - Severe<br>4, 4 - Life-threatening	If AE yes	
	Headache	1, 1 - Mild<br>2, 2 – Moderate<br>3, 3 - Severe	If AE yes	

		4, 4 - Life-threatening		
	Fatigue	1, 1 - Mild<br>2, 2 – Moderate<br>3, 3 - Severe<br>4, 4 - Life-threatening	If AE yes	
Respiratory	Difficulty breathing (wheezing or dyspnea	1, 1 - Mild<br>2, 2 – Moderate<br>3, 3 - Severe<br>4, 4 - Life-threatening	If AE yes	
	Cough	1, 1 - Mild<br>2, 2 – Moderate<br>3, 3 - Severe<br>4, 4 - Life-threatening	If AE yes	
Musculoskeletal	Joint pain	1, 1 - Mild<br>2, 2 – Moderate<br>3, 3 - Severe<br>4, 4 - Life-threatening	If AE yes	
	Muscle pain	1, 1 - Mild<br>2, 2 – Moderate<br>3, 3 - Severe<br>4, 4 - Life-threatening	If AE yes	
	Weakness	1, 1 - Mild<br>2, 2 – Moderate<br>3, 3 - Severe<br>4, 4 - Life-threatening	If AE yes	
Skin	Rash	1, 1 - Mild<br>2, 2 – Moderate<br>3, 3 - Severe<br>4, 4 - Life-threatening	If AE yes	
	Itchy skin	1, 1 - Mild<br>2, 2 – Moderate<br>3, 3 - Severe<br>4, 4 - Life-threatening	If AE yes	
Lymphatic	Swelling in armpit or groin	1, 1 - Mild<br>2, 2 – Moderate<br>3, 3 - Severe<br>4, 4 - Life-threatening	If AE yes	
	Pain in armpit or groin	1, 1 - Mild<br>2, 2 – Moderate<br>3, 3 - Severe<br>4, 4 - Life-threatening	If AE yes	
Genital	Men only: pain in your testicles or scrotum	1, 1 - Mild<br>2, 2 – Moderate<br>3, 3 - Severe<br>4, 4 - Life-threatening	If AE yes	
Gastrointestinal	Nausea	1, 1 - Mild<br>2, 2 – Moderate<br>3, 3 - Severe<br>4, 4 - Life-threatening	If AE yes	
	Vomiting	1, 1 - Mild<br>2, 2 – Moderate<br>3, 3 - Severe<br>4, 4 - Life-threatening	If AE yes	
	Diarrhea	1, 1 - Mild<br>2, 2 – Moderate<br>3, 3 - Severe<br>4, 4 - Life-threatening	If AE yes	

	Stomach Pain	1, 1 - Mild<br>2, 2 – Moderate<br>3, 3 - Severe<br>4, 4 - Life-threatening	If AE yes	
Unusual Swelling	New Swelling	1, 1 - Mild<br>2, 2 – Moderate<br>3, 3 - Severe<br>4, 4 - Life-threatening	If AE yes	
	Specify the location of the unusual swelling		If AE yes<br>AND<br>if there is any new swelling	Make Free text
Other	Other:		If AE yes	Make Free text
	Other Grade:	1, 1 - Mild<br>2, 2 – Moderate<br>3, 3 - Severe<br>4, 4 - Life-threatening	If AE yes	
	Start Date:		If AE yes	
	Ongoing?	0, No<br>1, Yes	If AE yes	
	Stop Date:		If not Ongoing	
	Did the participant report any symptoms that are Grade 2 or greater?<br><br>Grade 2 - notify a member of the medical team to conduct vital signs.<br><br>Grade 3 - refer participant for vital signs and physical examination by physician/medical team		POP UP	IF YES, THEN POPUP TO REFER PARTICIPANT FOR EXAMINATION BY PHYSICAN/MEDICAL TEAM
	Do you need to enter another Adverse Event for this Participant?	0, No<br>1, Yes	If AE yes	Pop up new AE form

Section Header	Field Label	Choices, Calculations, OR Slider Labels	Branching Logic (Show field only if...)	Comments
**Special Interest Events - LF**				
Lymphedema	Do you have swelling in your arms or legs (lymphedema)?	0, No<br>1, Yes		
	If lymphedema: Confirm and indicate which limbs are affected	1, Left arm<br>2, Right arm<br>3, Left leg<br>4, Right arm	If there is swelling	Needs to be checkboxes for multiple answers, not drop down
	Severity of lymphedema:	1, Mild<br>2, Moderate<br>3, Severe<br>4, Elephantiasis	If there is swelling	
Hydrocele	Are your testicles swollen?	0, No<br>1, Yes	If participant is male or unknown<br>AND if there is swelling and/or pain in testicles or scrotum	
	Is swelling there:	1, All the time<br>2, Only when carrying heavy things	If participant is male or unknown<br>AND if there is swelling and/or pain in testicles or scrotum	

Section Header	Field Label	Choices, Calculations, OR Slider Labels	Branching Logic (Show field only if...)	Comments
**VITAL SIGNS MONITORING**				
Automatic for any Grade 2 and above AE/SAE	Systolic blood pressure, sitting		If Physical Exam is required	Make Free text
	Sitting blood pressure	0, Not done		
	Diastolic blood pressure, sitting		If Physical Exam is required	Make Free text
	Diastolic blood pressure	0, Not done		
	Orthostatic information		If Physical Exam is required	SYSTOLIC BP AT LEAST 20 POINTS LOWER WHEN STANDING and diastolic BP at least 10 points lower when standing.
	Pulse rate (beats per minute):		If Physical Exam is required	Make Free text

	Pulse rate	0, Not done		
	Temperature (in Celsius):		If Physical Exam is required	Make Free text
	Temperature	0, Not done		

**Section Header**	**Field Label**	**Choices, Calculations, OR Slider Labels**	**Branching Logic (Show field only if...)**	**Comments**
**PHYSICAL EXAM MONITORING**				
ABNORMAL PHYSICAL FINDINGS	Was participant referred for further assessment?	0, No<br>1, Yes	If Physical Exam is required	
	Tender Lymph node locations:	1, Axilla<br>2, Inguinal<br>3, Scrotal	If Physical Exam is required<br>AND<br>Lymphatic Yes	
	For participants with GI symptoms, record the following:	1, Abdominal tenderness<br>2, Enlarged spleen<br>3, Enlarged liver	If Physical Exam is required<br>AND<br>GI symptom yes	
	Allergic reaction	0, No<br>1, Yes	If Physical Exam is required	
	Allergic reaction grade	0, 0 - No adverse event<br>1, 1 - Mild<br>2, 2 - Moderate<br>3, 3 - Severe<br>4, 4 - Life-threatening	If Physical Exam is required<br>AND<br>Allergic reaction	
	Lung wheezing	0, No<br>1, Yes	If Physical Exam is required<br>AND<br>Respiratory YES	
	Lung wheezing grade	0, 0 - No adverse event<br>1, 1 - Mild<br>2, 2 - Moderate<br>3, 3 - Severe<br>4, 4 - Life-threatening	If Physical Exam is required<br>AND<br>Respiratory Yes	
	Lymphangitis (streaks of redness, warmth, and swelling in arms or legs)	0, No<br>1, Yes	If Physical Exam is required	
	Lymphangitis Grade	0, 0 - No adverse event<br>1, 1 - Mild<br>2, 2 - Moderate<br>3, 3 - Severe<br>4, 4 - Life-threatening	If Physical Exam is required<br>AND<br>Lymphangitis present	
	Are there any abnormal physical findings?	0, No<br>1, Yes	If Physical Exam is required	
	Additional notes or comments:			

BODY SYSTEMS PHYSICAL EXAM	For participants with Grade 3 or higher, have a clinician fill out this form or refer to local medical facility for physical examination.		If Physical Exam is required	
	PE number		If Physical Exam is required	We will need option to have this be able to be done 13 times, to capture each Body System if needed
	Has anything changed since the last physical exam?	0, No<br>1, Yes<br>99, Non Applicable	If Physical Exam is required	
	Exam performed:	0, No<br>1, Yes	If Physical Exam is required	
	Reason Not Performed:		If Physical Exam is required	
	Exam Date:		If Exam performed	
	Body system:	1, Cardiac<br>2, Ear and Labyrinth<br>3, Endocrine and Lymph<br>4, Eye<br>5, Hepatobiliary, Renal and Urinary<br>6, Mouth, Throat and Gastrointestinal<br>7, Musculoskeletal and Connective Tissue<br>8, Nervous System<br>9, Reproductive System and Breast<br>10, Respiratory, Thoracic and Mediastinal<br>11, Skin and Subcutaneous Tissue<br>12, Vascular<br>13, Other	If Exam performed	
	Result:	1, Normal<br>2, Abnormal<br>3, Not Done	If Exam performed	
	Abnormal Findings:	FREE TEXT	If Exam performed	
	Clinically significant:	0, No<br>1, Yes	If Physical Exam is required	
Assess if an adverse event should be classified as serious based on the serious criteria defined in the protocol.	Is the adverse event serious?	0, No<br>1, Yes	If Physical Exam is required	POP UP<br>If Yes, open SAE Form module, if not, finished and follow up with participant tomorrow to ensure symptoms have resolved
	Reporting Clinician Name		If Physical Exam is required	FREE TEXT

APPENDIX 4: GUIDE TO ASSIGNING ADVERSE EVENT SEVERITY

(G*rade 0 = no symptoms; grade 5 = death from adverse event*)

Symptoms/Signs	Grades			
	**1. Mild**	**2. Moderate**	**3. Severe**	**4. Life-threatening**
Fever (non-axillary temperatures only)	38.0 – 39.0ºC	39.1 – 40.0ºC	> 40.0ºC	> 40.0ºC for > 48 hrs
Dizziness, giddiness, or fainting	Mild, not interfering with work or school	Moderate, unable to work or attend school for 1 day, but no fainting	Any loss of consciousness (fainting)	-
Confusion or excess drowsiness	Mild, not interfering with work or school	Moderate; confusion or drowsiness interfering with ability to work	Confusion, loss of memory, or sleepiness interfering with activities of daily living	Delerium, inability rouse, or coma
Fatigue	Mild, not interfering with work or school	Moderate, unable to work or attend school at least 1 day	Unable to perform activities of daily living, > 1day	Required hospitalization
Headache	Mild pain not interfering with work or school	Moderate pain; pain or analgesics interfering with ability to work or attend school	Severe pain; pain or analgesics interfering with activities of daily living	Disabling, duration > 48 hr
Cough	Mild, relieved by non-prescription medication	Requiring narcotic antitussive	Severe cough or coughing spasms, poorly controlled by treatment	Hospitalization or respiratory failure requiring mechanical ventilation
Difficulty breathing (wheezing or dyspnea)	Mild, not interfering with work or school	Moderate, unable to work or attend school for 1 day	Severe, more than 1 day and required transfer to clinic or hospital	Hospitalization or respiratory failure requiring mechanical ventilation
Nausea	Able to eat	Oral intake significantly decreased	No significant intake, requiring IV fluids	-
Vomiting	1 episode in 24 hours over pretreatment	2-5 episodes in 24 hours over pretreatment	≥ 6 episodes in 24hours, or need for IV fluids (0upatient)	Hemodynamic collapse or overnight hospitalization

Appendix 4: GUIDE TO ASSIGNING ADVERSE EVENT SEVERITY

(*Grade 0 = no symptoms; grade 5 = death from adverse event*)

Symptoms/Signs	Grades			
	**1. Mild**	**2. Moderate**	**3. Severe**	**4. Life-threatening**
Diarrhea	Increase of < 4 stools/day over pre-treatment	Increase of 4-6 stools/ day, or nocturnal stools	Increase of ≥ 7 stools/ day or need for outpatient parenteral support for dehydration	Physiologic consequences wth hemodynamic collapse or requiring hospitalization
Abdominal pain	Mild pain not interfering with work or school	Moderate pain; pain or analgesics interfering with ability to work or attend school	Severe pain; pain or analgesics interfering with activities of daily living	Disabling, duration > 48 hr
Unusual swelling (beyond baseline lymphedema)	Mild, not interfering with work or school	Moderate, unable to work or attend school 1 day	Severe, unable to work/school >1 day	Severe, limiting activities of daily living (unable to walk) > 2 days
Joint or muscle pain	Mild pain not interfering with work or school	Moderate pain; pain or analgesics interfering with ability to work or attend school	Severe pain; pain or analgesics interfering with activities of daily living	Disabling, duration > 48 hr
Swelling or pain in your armpit or groin	Mild, not interfering with work or school	Moderate, unable to work or attend school 1 day	Severe, unable to work/school >1 day	Severe, limiting activities of daily living (unable to walk) > 2 days
Men only: testicular or scrotal pain	Mild, not interfering with work or school	Moderate, unable to work or attend school 1 day	Severe, unable to work/school >1 day	Severe, limiting activities of daily living (unable to walk) > 2 days
Itching skin	Mild, not interfering with work or school	Moderate, unable to work or attend school 1 day	Severe, unable to work/school >1 day	
Rash	Localized rash (covers only one part of the body)	Diffuse rash (covers multiple parts of the body)	Diffuse rash (covers multiple parts of the body) AND has any blisters or ulcers or mouth sores	Extensive areas with blisters or ulcers OR peeling or blackening of skin
Other illness or symptoms	Mild, not interfering with work or school	Moderate, unable to work or attend school at least 1 day	Unable to perform activities of daily living, > 1day	Required hospitalization

Post-Exam Assessment	Grades			
	**1. Mild**	**2. Moderate**	**3. Severe**	**4. Life-threatening**

Appendix 4: GUIDE TO ASSIGNING ADVERSE EVENT SEVERITY

(*Grade 0 = no symptoms; grade 5 = death from adverse event*)

Acute allergic reaction	Transient rash, drug Fever <38ºC (<100.4ºF)	Urticaria, drug fever ≥38ºC (≥100.4ºF) and/or asymptomatic bronchospasm	Symptomatic bronchospasm, requiring parenteral medication(s) with or without urticaria	Anaphylaxis with hypotension required hospitalization
Hypotension (low blood pressure)	Changes, but not requiring therapy (including transient orthostatic hypotension)	Requiring brief fluid replacement (such as oral rehydration) but not hospitalization	Requiring i.v. fluids without overnight hospitalization. No sequelae.	Required overnight hospitalization for i.v. fluids, or Shock (acidemia and impaired vital organ function due to tissue hypoperfusion)
Lymphangitis	Mild, not interfering with work or school	Moderate, unable to work or attend school 1 day	Severe, unable to work/school >1 day	Severe, limiting activities of daily living (unable to walk) > 2 days

Note on general aspects of grading
0 = No adverse event or within normal limits
1 = Mild adverse event, awareness of symptom, but easily tolerated and does not interfere with normal activities (work or school)
2 = Moderate adverse event, interferes with normal activities (work or school) at least 1 day
3 = Severe and undesirable adverse event; interferes with ADL/self-care, requires medical assessment
4 = Potentially life-threatening or disabling adverse event; requires transfer to medical facility
5 = Death

Note: Any event ≥ grade 2 requires a nurse review with vital signs. Any event ≥ grade 3 requires a physician review and an Adverse Event Evaluation and Report Form.

APPENDIX 5: SERIOUS ADVERSE EVENT (SAE) FORM [EXAMPLE]

PROTOCOL: DOLF IDA	Country: _____	Site#: _____	Participant Barcode:

SECTION 1: REPORT TYPE

☐ **Initial** - Date: __-__-____ (DD-MMM-YYYY)

☐ **Follow-up #**_____ - Date: __-__-____ (DD-MMM-YYYY)

SECTION 2: DEMOGRAPHICS

Gender: ☐ Female ☐ Male

Date of Birth: ___-_____ (MMM-YYYY)

SECTION 3: ADVERSE EVENT INFORMATION

SAE Term *(concise medical diagnosis)*: _____

Start Date: __-__-____ (DD-MMM-YYYY)

End Date: __-__-____ (DD-MMM-YYYY)

Serious Criteria *(check all that apply):*

☐ Inpatient or Prolonged Hospitalization - If yes, Date of Admission: __-__-____ (DD-MMM-YYYY)

Date of Discharge: __-__-____ (DD-MMM-YYYY)

☐ Life-threatening (immediate risk of death)

☐ A persistent or significant disability/incapacity

☐ Congenital Anomaly or Birth Defect

☐ Other Serious or Important Medical Event

☐ Death - If yes, Cause of Death: _____ Date of Death: __-__-____ (DD-MMM-YYYY)

Was autopsy completed? ☐ No ☐ Yes - *If yes, please forward report*

Is death certificate available? ☐ No ☐ Yes - *If yes, please forward*

Outcome *(check one):*

☐ Recovered/Resolved

☐ Recovered/Resolved with sequelae *(describe sequelae in narrative)*

☐ Recovering/Resolving

☐ Not Recovered/Not Resolved

☐ Fatal

☐ Unknown

CTCAE Grade Version 4.03 / Severity *(check one):*

☐ Grade 1/Mild ☐ Grade 2/Moderate ☐ Grade 3/Severe ☐ Grade 4/Life-threatening ☐ Grade 5/Death

SECTION 4: STUDY DRUG INFORMATION

MDA Treatment Date	Albendazole (400 mg tablets)	DEC (100 mg tablets)	Ivermectin (3 mg tablets)	Relationship of SAE to MDA *(check one)*
__-__-____ DD-MMM-YYYY	# Tabs ☐	# Tabs ☐	# Tabs ☐<br>NA ☐	☐ Not Related<br>☐ Possibly Related<br>☐ Related

Possible alternate cause other than Study Drug *(select all that apply)*:

☐ Study Disease-related

☐ Pre-existing condition - Specify: _____

☐ Concomitant medication - Specify: _____

☐ Other - Specify: _____

APPENDIX 5: SERIOUS ADVERSE EVENT (SAE) FORM [EXAMPLE]

PROTOCOL: DOLF IDA	Country: _____	Site#: _____	Participant Barcode:

SECTION 5: RELEVANT LABORATORY/DIAGNOSTIC TESTS ☐ None

Test Name	Date	Result	Unit	Normal Range
	___-___-_____ DD-MMM-YYYY			
	___-___-_____ DD-MMM-YYYY			
	___-___-_____ DD-MMM-YYYY			
	___-___-_____ DD-MMM-YYYY			
	___-___-_____ DD-MMM-YYYY			

SECTION 6: RELEVANT CONCOMITANT MEDICATIONS ☐ None

Medication	Start Date	Stop Date or Ongoing	Dose & Unit	Frequency	Route	Indication
	___-___-_____ DD-MMM-YYYY	___-___-_____ OR ☐ Ongoing DD-MMM-YYYY				
	___-___-_____ DD-MMM-YYYY	___-___-_____ OR ☐ Ongoing DD-MMM-YYYY				
	___-___-_____ DD-MMM-YYYY	___-___-_____ OR ☐ Ongoing DD-MMM-YYYY				
	___-___-_____ DD-MMM-YYYY	___-___-_____ OR ☐ Ongoing DD-MMM-YYYY				
	___-___-_____ DD-MMM-YYYY	___-___-_____ OR ☐ Ongoing DD-MMM-YYYY				

SECTION 7: RELEVANT MEDICAL HISTORY ☐ None

Diagnosis	Start Date	Stop Date or Ongoing
	___-___-_____ DD-MMM-YYYY	___-___-_____ OR ☐ Ongoing DD-MMM-YYYY
	___-___-_____ DD-MMM-YYYY	___-___-_____ OR ☐ Ongoing DD-MMM-YYYY
	___-___-_____ DD-MMM-YYYY	___-___-_____ OR ☐ Ongoing DD-MMM-YYYY
	___-___-_____ DD-MMM-YYYY	___-___-_____ OR ☐ Ongoing DD-MMM-YYYY
	___-___-_____ DD-MMM-YYYY	___-___-_____ OR ☐ Ongoing DD-MMM-YYYY

SECTION 8: NARRATIVE SUMMARY

Describe the event in detail from onset through resolution. Include rationale for causality and any interventions given.

APPENDIX 5: SERIOUS ADVERSE EVENT (SAE) FORM [EXAMPLE]

**PROTOCOL:** DOLF IDA	**Country:** _____	**Site#:** _____	**Participant Barcode:**

**SECTION 9: REPORTER INFORMATION**	
Reporter Name (if not Investigator): _____	Location: _____
I, the undersigned investigator, attest that I have reviewed this SAE Report and agree with the content.	
Investigator Name:	
Investigator Signature:	Date: __-__-____<br>DD-MMM-YYYY

APPENDIX 5A: REQUIRED REPORTING & GUIDELINES FOR SAE(S)

A physician will review all $\geq$ grade 3 severity symptoms to decide if the participant is suffering from a suspected serious adverse event. These two events are not mutually exclusive and the majority of grade 3 adverse events will not be classified as SAE. The term "severe" is often used to describe the intensity (severity) of a specific event (as in mild, moderate, or severe myocardial infarction); the event itself, however, may be of relatively minor medical significance (such as severe headache). This is not the same as "serious," which is based on patient/event outcome or action criteria usually associated with events that pose a threat to a patient's life or functioning. Seriousness (not severity) serves as a guide for defining regulatory reporting obligations. A SAE form (Appendix 5) should be completed for every suspected or definite SAE. All SAE must be reported promptly (see 7.5).

Required Reporting

A written report or case report form (CRF—in this study, the SAE form) must be completed for the events listed below. If this is done through the electronic database it will be available for viewing by the Global Medical Monitor and PIs. If it is completed on a paper form it must be scanned and emailed. sent from the local physician and/or local medical monitor by email (scanned records)

Guidelines for Reporting - Standard Reporting Information

The following information should be included in the initial report/CRF (additional information may be requested):

Minimum Criteria for Reporting

Information for final description and evaluation of a case report may not be available within the required time frames for reporting outlined below. Initial reports should be submitted within the prescribed time as long as the following minimum criteria are met: an ***identifiable patient****; **an identifiable reporting source**; and* ***an event or outcome that can be identified as serious***. Follow-up information should be actively sought and submitted as it becomes available.

Complete the following information if available on the initial report and complete a follow-up report as new or additional information becomes available as noted below:

- Description of the event
  Date, time of onset
  Clinical history
  Associated signs and symptoms
  Temporal association with study agent
  Medical management, including rationale
  Pertinent laboratory tests
  Severity – *see definitions or toxicity score*
  Causal relationship to the study drug/vaccine

- <u>Other information</u>
  Relevant past medical history
  Concomitant medications
  Autopsy report or expectation of an autopsy in the case of death

- <u>Outcome of event</u>
  Date, time of resolution, if resolved

- <u>Plans for study participant</u>
  Follow-up
  Treatment of event
  Return to treatment/Contraindicate

- <u>Location/Study Centre</u>

- <u>Reporting Physician</u>

- <u>Verification of notification to IRB and Safety Monitor or DSMB</u>

Definitions

- <u>Adverse Event (AE):</u>

  Any untoward medical occurrence, including dosing errors, that may arise during administration of study agent, and which may or may not have a causal relationship with the study agent.

- <u>Unexpected Adverse Event:</u>

  Any adverse event that has not been previously observed (i.e., included in the labelling), whether or not the event is anticipated because of the pharmacologic properties of the study agent.

- <u>Serious Adverse Event (SAE):</u>

  Any adverse event occurring at any dose that results in any of the following outcomes:

  a. Death

  b. Life threatening – defined as an event that places the patient or participant, in the view of the Investigator, at *immediate risk* of death from the reaction as it occurred. (Note; this does not include a reaction that, had it occurred in a more severe form, might have caused death.)

  c. Requires inpatient hospitalization or prolongation of existing hospitalization

  d. Results in a congenital anomaly or birth defect

e. Results in a persistent or significant disability or incapacity

f. Important medical events that may not result in death, be life-threatening, or require hospitalization may be considered a serious adverse experience when, based upon appropriate medical judgment, they may jeopardize the patient or participant and may require medical or surgical intervention to prevent one of the outcomes listed in this definition. *(The event might be defined as serious based on progression of grade if Toxicity Tables are being used.)*

Severity

Adverse events should be assessed by the on-site investigator as to their severity and/or intensity and graded as follows:

1) Mild: awareness of symptom, but easily tolerated and does not interfere with normal activities (work or school)
2) Moderate: enough discomfort to interfere with normal activities (work or school) at least 1 day
3) Severe: incapacitating and interferes with ADL/self-care, requires medical assessment
4) Life threatening: Potentially life-threatening or disabling adverse event; requires transfer to medical facility
5) Death

Relationship or Association with Use of Study Agent or Participation in the Study

Causal relationship with the investigational study treatment must be assessed by the on-site investigator using the following or similar terms:

- **Definite** – clear-cut temporal association, with a positive re-challenge test or laboratory confirmation.
- **Probable** – clear-cut temporal association, with improvement upon drug withdrawal, and not reasonably explained by the participant’s known clinical state.
- **Possible** – less clear temporal association; other aetiologies are possible.
- **None** – no temporal association with the study drug; related to other aetiologies such as concomitant medications or conditions, or participant’s known clinical state.

APPENDIX 6: PARTICIPANT INFORMATION STATEMENT AND CONSENT FORM [EXAMPLE]

**HREC Project Number:**	36205		
**Research Project Title:**	FIT – Fiji Integrated Therapy		
**Principal Researcher:**	Prof Andrew Steer, Murdoch Children's Research Institute		
**Version Number:**	5.0	**Version Date:**	01/06/2018

Thank you for taking the time to read this **Participant Information Statement and Consent Form**. We would like to invite you to participate in a research project that is explained below.

This document is 6 pages long. Please make sure you have all the pages. It is also available in iTaukei.

What is an Information Statement?
An information statement and consent form tells you about the research project. It clearly explains exactly what the research project will involve. The information is to help you to decide whether or not you would like to take part in the research. Please read it carefully.

Before you decide to take part or not, you can ask us any questions you have about the project. You may want to talk about the project with your family, friends, nurse or community health care worker.

Taking part in the research is up to you

It is your choice whether or not you take part in the research. You do not have to agree if you do not want to. If you decide you do not want to take part, it will not affect the treatment and care you get at the health centre/hospital.

Signing the form

If you would like to take part in the research project, please sign the consent form at the end of this document. By signing the form you are telling us that you:

- understand what you have read
- had a chance to ask questions and received satisfactory answers
- consent to taking part in the project.

We will give you a copy of this form to keep.

APPENDIX 6: PARTICIPANT INFORMATION STATEMENT AND CONSENT FORM

1. What is the research project about?

Lymphatic filariasis (LF, known as *"Waqaqa"* in Fiji) is a disease carried by mosquitoes and caused by tiny worms in the blood. It can cause complications like swelling of the legs (lymphedema) or the scrotum (hydrocele). The Fijian Ministry of Health has given community mass drug administration (MDA) treatment for these worms every year in order to stop LF in Fiji. Though treatment has been given annually since 2002, too many people are still infected with LF in your community.

Scabies (known as *"karo karo"* in Fiji) is a skin disease caused by very small mites. The mites burrow into the skin and cause itching. Scabies can spread from contact with other people's infected skin. Scabies is very common in Fiji. Giving treatment to everyone in your community for scabies can reduce the chances of it spreading.

Soil Transmitted Helminths (STH, known as intestinal worms or *"ukomu"* in Fiji) are worms that come from the soil and live in our intestines. They can cause stomach pain, tiredness, weight loss and poor growth in children. We can get STH from walking barefoot on soil or eating unwashed fruit and vegetables.

The main purpose of the study that happened last year in your community was to find out if adding ivermectin (IVM) to the normal treatment for LF in Fiji, diethylcarbamazine (DEC) and albendazole (ALB), would make any difference to how people feel after treatment. We found that there was no difference adding the extra medication to the safety of the drugs. The World Health Organization has changed their recommendation to three drug treatment for LF MDA for Fiji. This has been approved by the Fiji National LF Taskforce Committee.

This time we will also look at how effective the different drug combinations given last year were at killing LF, scabies and STH. We do this by asking everyone living in your community today to have tests for LF, their skin looked at for scabies and those willing, testing their stool sample for intestinal worms.

Everyone on the island at the time of the study is invited to participate, regardless of whether they took part last year.

All villages this year will be offered the three drug treatment (IDA). If you or anyone in the house is found to have scabies you will be treated with permethrin cream. This is because we know that ivermectin kills scabies but don't yet know if one dose is enough. To be sure the scabies is cured we will give permethrin as well.

If you are pregnant, breastfeeding within the first 7 days of delivery, less than 15kg or very unwell you cannot safely take LF MDA. If you are aged 2-4 years you cannot safely take ivermectin but can still safely take DEC and ALB.

If you are found to have LF or STH but can't safely take the medication, we will ask the health centre to give you treatment later when you are able to safely take the medication.

2. Who is funding this research project?

The Bill and Melinda Gates Foundation is funding this study.

3. Why am I being asked to take part?

We are asking you to be in the study because you live on one of the islands selected for this study where you may become sick with LF, scabies and STH.

4. What do I need to do in this research project?

There are a number of steps involved in this research project. We will ask you to:

- Read and sign a written consent form if you intend to take part.
- Answer some simple questions about yourself, like age and gender.
- Measure your weight.
- Take a blood sample by finger-prick to test you for LF infection. If this tells us you have adult worms, we will test your blood again by finger-prick to look for baby worms. Some blood will be dried on paper to test later for the infections under study. We will not test you, if you are less than 2 years old.
- Look at your skin for scabies and skin sores.

Provide a fresh faecal (waste/poo) sample that you collected at home to test for intestinal worms (only if you are happy to do so).

- Answer questions about your house environment, places you have lived in the last 12 months, involvement in part MDA and provide your opinion about MDA.
- Answer questions about your medical history.
- Take the medication in front of the nurse, unless the team have identified a reason why it would not be safe for you.

5. Can I withdraw from the project?

You can stop taking part in the project at any time. You just need to tell us so. You do not need to tell us the reason why. If you leave the project, we will use any information already collected unless you tell us not to.

6. What are the possible benefits for me and other people in the future?

This study will be giving treatment to everyone in the community at the same time regardless if they have infection or not. This is called mass drug administration (MDA) and is how LF is normally treated in Fiji. If you have LF, scabies and/or intestinal worms this study will help treat your infection. If you don't have one of these infections, then you will still benefit from everyone around you being treated at the same time. This reduces the chance of you getting one of the infections in the future.

The investigators hope that the information learned from this study will benefit people in Fiji as well as in other areas of the world affected by LF, scabies and STH. From this work it may be possible to reduce the number of MDA treatments needed to eliminate this problem in Fiji.

7. **What are the possible risks, side-effects, discomforts and/or inconveniences?**

Side effects from the medications are more likely if you have LF, STH or scabies infection because your body is reacting to the dying worms and/or mites. You might experience itching and swelling of face, headache, joint pain, tiredness, weakness, dizziness, fainting, nausea, vomiting, stomach pain, diarrhea, fever, painful glands in groin/neck/armpits, skin rash, racing heart, or an allergic reaction (itchy rash, difficulty breathing, tightness in chest, swelling of face/tongue). We want to collect information about how your body reacts to the study medications.

The risk of taking blood from a finger prick is small. You might become dizzy, have some slight pain and/or bruising. Young children may cry if they become scared or uncomfortable. Rarely there might be too much blood or an infection may occur.

If you feel unwell, you can approach the study team or health worker for simple medication to relieve your symptoms. If you are very unwell you should go to the local clinic.

8. What will be done to make sure my information is confidential?

Any information we collect for this research project that can identify you will be treated as confidential. We can disclose the information only with your permission, except as required by law. The information will be re-identifiable. This means that we will remove your name and give the information a special code number. Only the research team can match your name to the code number, if it is necessary to do so. The information will be entered in a password-protected computer database. Any paper data forms will be stored securely in a locked room at the Fiji Centre for Communicable Disease Control (FCCDC) in Suva. The following people may access information collected as part of this research project:

- The research team involved with this project.
- An Australian Human Research Ethics Committee.
- The Fiji National Research Ethics Review Committee, Fiji.

We will keep the information until the youngest participant in this project turns 25 years old. After this time, it may be destroyed. The samples collected as part of this project will be destroyed after testing.

In accordance with Fijian National Health Research Policy 1999 and Health Information Policy 2011, as well as Australian and/or Victorian privacy and other relevant laws, you have the right to access and correct the information we collect and store about you. Please contact us if you would like to access your information.

When we write or talk about the results of this project, only group results will be analysed and information will be provided in such a way that you cannot be identified. No information concerning the study or the data will be released to any unauthorized third party.

9. Will I be informed of the results when the research project is finished?

We will tell you the results of your LF rapid test and skin examination on the day. The results for intestinal worms and LF baby worms (if done) will be given to your local health centre to be available at your request. A summary of the overall project results will be sent to your district nurse. Your results will not be able to be identified from the summary.

10. Who should I contact for more information?

If you would like more information about the project or if you need to speak to a member of the research team in an emergency please contact:

Name: Sarah Gwonyoma
Telephone: (+679) 7224288 or (+679) 2983768 or (+679) 3560419
Email: fitstudy2018@gmail.com
OR
Name: Dr Myra Hardy
Telephone: (+679) 7166748 or (+679) 8011098 or (+679) 3560418
Email: myra.hardy@rch.org.au

If you have any concerns and/or complaints about the project, the way it is being conducted or your rights as a research participant, and would like to speak to someone independent of the project, please contact:

Chair, Fiji National Research Ethics Review Committee, Fiji, Telephone (+679) 3221 424

APPENDIX 6: PARTICIPANT INFORMATION STATEMENT AND CONSENT FORM

CONSENT FORM

HREC Project Number: 36205

Research Project Title: FIT – Fiji Integrated Therapy

Version Number: 5.0 **Version Date:** 01/06/2018

- I have read, or someone has read to me in a language that I understand, the information statement version listed above and I understand its contents.
- I believe I understand the purpose, extent and possible risks of my involvement in this project.
- I voluntarily consent to take part in this research project.
- I have had an opportunity to ask questions and I am satisfied with the answers I have received.
- I understand that this project has been approved by an Australian Human Research Ethics Committee and by the Fiji National Research Ethics Review Committee, and will be carried out in line with the National Statement on Ethical Conduct in Human Research (2007) – including all updates.
- I understand I will receive a copy of this Information Statement and Consent Form.

Participant Name	Participant Signature	Date
Name of Witness to Participant's Signature	Witness Signature	Date

Declaration by researcher: I have explained the project to the participant who has signed above, and believe that they understand the purpose, extent and possible risks of their involvement in this project.

Research Team Member Name	Research Team Member Signature	Date

Note: All parties signing the Consent Form must date their own signature

APPENDIX 6A: PARENT/GUARDIAN INFORMATION STATEMENT AND CONSENT FORM [EXAMPLE]

**HREC Project Number:**	36205		
**Research Project Title:**	FIT – Fiji Integrated Therapy		
**Principal Researcher:**	Prof Andrew Steer, Murdoch Children's Research Institute		
**Version Number:**	5.0	**Version Date:**	01/06/2018

Thank you for taking the time to read this **Parent/Guardian Information Statement and Consent Form**. We would like to invite your child to participate in a research project that is explained below.

This document is 6 pages long. Please make sure you have all the pages. It is also available in iTaukei.

What is an Information Statement and Consent Form?

An information statement and consent form tells you about the research project. It clearly explains exactly what the research project will involve. The information is to help you to decide whether or not you would like your child to take part in the research. Please read it carefully.

Before you decide for your child to take part or not, you can ask us any questions you have about the project. You may want to talk about the project with your family, friends, nurse or community health care worker.

Taking part in the research is up to you

It is your choice whether or not your child takes part in the research. You do not have to agree if you do not want to. If you decide you do not want your child to take part, it will not affect the treatment and care that they get at the health centre/hospital.

Signing the form

If you would like your child to take part in the research project, please sign the consent form at the end of this document. By signing the form you are telling us that you:

- understand what you have read
- had a chance to ask questions and received satisfactory answers
- consent for your child to take part in the project.

We will give you a copy of this form to keep.

APPENDIX 6A: PARENT INFORMATION STATEMENT AND CONSENT FORM

1. What is the research project about?

Lymphatic filariasis (LF, known as *"Waqaqa"* in Fiji) is a disease carried by mosquitoes and caused by tiny worms in the blood. It can cause complications like swelling of the legs (lymphedema) or the scrotum (hydrocele). The Fijian Ministry of Health has given community mass drug administration (MDA) treatment for these worms every year in order to stop LF in Fiji. Though treatment has been given annually since 2002, too many people are still infected with LF in your community.

Scabies (known as *"karo karo"* in Fiji) is a skin disease caused by very small mites. The mites burrow into the skin and cause itching. Scabies can spread from contact with other people's infected skin. Scabies is very common in Fiji. Giving treatment to everyone in your community for scabies can reduce the chances of it spreading.

Soil Transmitted Helminths (STH, known as intestinal worms or *"ukomu"* in Fiji) are worms that come from the soil and live in our intestines. They can cause stomach pain, tiredness, weight loss and poor growth in children. We can get STH from walking barefoot on soil or eating unwashed fruit and vegetables.

The main purpose of the study that happened last year in your community was to find out if adding ivermectin (IVM) to the normal treatment for LF in Fiji, diethylcarbamazine (DEC) and albendazole (ALB), would make any difference to how people feel after treatment. We found that there was no differenc by adding the extra medication to the safety of the drugs. The World Health Organization has changed their recommendation to three drug treatment for LF MDA for Fiji. This has been approved by the Fiji National LF Taskforce Committee.

This time we will look at how effective the different drug combinations given last year were at killing LF, scabies and STH. We do this by asking everyone living in your community today to have tests for LF, their skin looked at for scabies and throse that are willing, testing their stool sample for intestinal worms.

Everyone on the island at the time of the study is invited to participate, regardless of whether they took part last year.

All villages this year will be offered the three drug treatment (IDA). If your child or anyone in the house is found to have scabies they will be treated with permethrin cream. This is because we know that ivermectin kills scabies but don't yet know if one dose is enough. To be sure the scabies is cured we will give permethrin as well.

If your child is less than 2 years, less than 15kg or very unwell, they cannot safely take the LF MDA. If your child is aged 2-4 years they cannot safely take ivermectin but can still safely take DEC and ALB.

If they are found to have LF or STH but can't safely take the medication, we will ask the health centre to give them treatment later when they are able to safely take the medication.

2. Who is funding this research project?

The Bill and Melinda Gates Foundation is funding this study.

3. Why am I being asked to take part?

We are asking your child to be in the study because they live on one of the islands selected for this study where they may become sick with LF, scabies and STH.

4. What do I need to do in this research project?

There are a number of steps involved in this research project. We will ask you to:

- Read and sign a written consent form if you intend for your child to take part.
- Answer some simple questions about your child, like age and gender.
- Allow us to measure your child's weight.
- Allow us to take a blood sample by finger-prick to test your child for LF infection. If this tells us they have adult worms, we will test their blood again by finger-prick to look for baby worms. Some blood will be dried on paper to test later for the infections under study. We will not test them if they are less than 2 years old.
- Allow us to look at their skin for scabies and skin sores.
- Provide a fresh faecal (waste/poo) sample from your child that you collected at home to test for intestinal worms (only if you are happy to do so).
- Answer questions about your house environment, places your child has lived in the last 12 months, their involvement in past MDA and if they are 14 years and older, allow us to ask their opinion about MDA.
- Answer questions about your child's medical history.
- Allow your child to take the medication in front of the nurse, unless the team have identified a reason why it would not be safe for your child.

5. Can I withdraw from the project?

You can stop your child taking part in the project at any time. You just need to tell us so. You do not need to tell us the reason why. If your child leaves the project, we will use any information already collected unless you tell us not to.

6. What are the possible benefits for me and other people in the future?

This study will be giving treatment to everyone in the community at the same time regardless if they have infection or not. This is called mass drug administration (MDA) and is how LF is normally treated in Fiji. If your child has LF, scabies and/or intestinal worms this study will help treat their infection. If they don't have one of these infections, then they will still benefit from everyone around them being treated at the same time. This reduces the chance of them getting one of the infections in the future.

The investigators hope that the information learned from this study will benefit people in Fiji as well as in other areas of the world affected by LF, scabies and STH. From this work it may be possible to reduce the number of MDA treatments needed to eliminate this problem in Fiji.

7. What are the possible risks, side-effects, discomforts and/or inconveniences?

Side effects from the medications are more likely if your child has LF, STH or scabies infection because their body is reacting to the dying worms and/or mites. They might experience itching and swelling of face, headache, joint pain, tiredness, weakness, dizziness, fainting, nausea, vomiting, stomach pain,

diarrhea, fever, painful glands in groin/neck/armpits, skin rash, racing heart, or an allergic reaction (itchy rash, difficulty breathing, tightness in chest, swelling of face/tongue). We want to collect information about how their body reacts to the study medications.

The risk of taking blood from a finger prick is small. They might become dizzy, have some slight pain and/or bruising. Young children may cry if they become scared or uncomfortable. Rarely there might be too much blood or an infection may occur.

If your child feels unwell, they will be treated firstly by the study team and if they are found to be very unwell then we will help them to get care from the local health centre or hospital. The chance of this is small. All health centre and hospital care will be provided by the public health service at no extra cost to you.

8. What will be done to make sure my information is confidential?

Any information we collect for this research project that can identify your child will be treated as confidential. We can disclose the information only with your permission, except as required by law. The information will be re-identifiable. This means that we will remove your name and give the information a special code number. Only the research team can match your child's name to the code number, if it is necessary to do so. The information will be entered in a password-protected computer database. Any paper data forms will be stored securely in a locked room at the Fiji Centre for Communicable Disease Control (FCCDC) in Suva. The following people may access information collected as part of this research project:

- The research team involved with this project.
- An Australian Human Research Ethics Committee.
- The Fiji National Research Ethics Review Committee, Fiji.

We will keep the information until the youngest participant in this project turns 25 years old. After this time, it may be destroyed. The samples collected as part of this project will be destroyed after testing.

In accordance with Fijian National Health Research Policy 1999 and Health Information Policy 2011, as well as Australian and/or Victorian privacy and other relevant laws, you have the right to access and correct the information we collect and store about your child. Please contact us if you would like to access your child's information.

When we write or talk about the results of this project, only group results will be analysed and information will be provided in such a way that your child cannot be identified. No information concerning the study or the data will be released to any unauthorized third party.

9. Will I be informed of the results when the research project is finished?

We will tell you the results of your child's LF rapid test and skin examination on the day. The results for intestinal worms and LF baby worms (if done) will be given to your local health centre to be available at your request. A summary of the overall project results will be sent to your district nurse. Your child's results will not be able to be identified from the summary.

10. Who should I contact for more information?

If you would like more information about the project or if you need to speak to a member of the research team in an emergency please contact:

Name: Sarah Gwonyoma
Telephone: (+679) 7224288 or (+679) 2983768 or (+679) 3560419
Email: fitstudy2018@gmail.com
OR
Name: Dr Myra Hardy
Telephone: (+679) 7166748 or (+679) 8011098 or (+679) 3560418
Email: myra.hardy@rch.org.au

If you have any concerns and/or complaints about the project, the way it is being conducted or your child's rights as a research participant, and would like to speak to someone independent of the project, please contact:

Chair, Fiji National Research Ethics Review Committee, Fiji, Telephone (+679) 3221 424

APPENDIX 6A: PARENT INFORMATION STATEMENT AND CONSENT FORM

CONSENT FORM

**HREC Project Number:**	36205
**Research Project Title:**	FIT – Fiji Integrated Therapy
**Version Number:** 5.0	**Version Date:** 01/06/2018

- I have read, or someone has read to me in a language that I understand, the information statement version listed above and I understand its contents.
- I believe I understand the purpose, extent and possible risks of my child's involvement in this project.
- I voluntarily consent for my child to take part in this research project.
- I have had an opportunity to ask questions and I am satisfied with the answers I have received.
- I understand that this project has been approved by an Australian Human Research Ethics Committee and by the Fiji National Research Ethics Review Committee, and will be carried out in line with the National Statement on Ethical Conduct in Human Research (2007) – including all updates.
- I understand I will receive a copy of this Information Statement and Consent Form.

____
Child's Name

Parent/Guardian Name	Parent/Guardian Signature	Date
Name of Witness to Parent/Guardian's Signature	Witness Signature	Date

Declaration by researcher: I have explained the project to the parent/guardian who has signed above, and believe that they understand the purpose, extent and possible risks of their child's involvement in this project.

Research Team Member Name	Research Team Member Signature	Date

Note: All parties signing the Consent Form must date their own signature

APPENDIX 7: TREATMENT ACCEPTABILITY STUDY PROTOCOL

"Treatment acceptability study following the Community Based Safety Study of 2-drug (Diethylcarbamazine and Albendazole) versus 3-drug (Ivermectin, Diethylcarbamazine and Albendazole) Therapy for Lymphatic Filariasis in Fiji, Haiti, India, Indonesia and Papua New Guinea"

Principal Investigator for the acceptability study
Alison Krentel PhD, MScPH
Bruyère Research Institute
85 Primrose Avenue, room 308-B
Ottawa, ON K1R 6M1, Canada
Tel: +1(613) 562-6262 x2954
Email: akrentel@yahoo.ca

Study Site Principal Investigators
Myra Hardy MD
University of Melbourne
Melbourne, Australia
Email: Myra.Hardy@rch.org.au

Josaia Samuela
Ministry of Health and Medical Services Fiji
Suva, Fiji
Email : josaia.samuela@health.gov.fj

Fiji Co-Investigators
John Kaldor, MD, PhD
Public Health Interventions Research Group
Kirby Institute, University of New South Wales
Sydney, Australia
Tel: +61 (0)2 9385 0900
email: jkaldor@kirby.unsw.edu.au

Lucia Romani, PhD
Kirby Institute, University of New South Wales
Sydney, Australia
email: lromani@kirby.unsw.edu.au

Andrew Steer MD, PhD
Department of General Medicine, Royal Children's Hospital
Centre for International Child Health, University of Melbourne
Melbourne, Australia
email: andrew.steer@rch.org.au

Margot Whitfeld, MD
Department of Dermatology
St. Vincent's Hospital

APPENDIX 7: TREATMENT ACCEPTABILITY STUDY PROTOCOL

Sydney, Australia
email: mwhitfeld@stlderm.com.au

Co-investigators in Haiti
Dr. Jean Frantz Lemoine
Guy Emmanuel Pavilus, IMA World Health
Carl Fayette, IMA World Health
Abdel Direny, consultant
Valery Madsen De Rochars, consultant
Christine Dubray, CDC Atlanta

Co-investigators in Indonesia
Taniawati Supali, Universitas Indonesia
Adriani Lomi Ga, Government of Nusa Tenggara Timur
Maddi Djara, consultant

Co-investigators in Papua New Guinea
Dr. Leanne Robinson
Krufinta Bun, Case Western University
Cade Howard, Case Western University

Statistical support
Ken Schechtman, Washington University in St. Louis

A. Summary

**Title of the study:**	Treatment acceptability study following the Community Based Safety Study of 2-drug (Diethylcarbamazine and Albendazole) versus 3-drug (Ivermectin, Diethylcarbamazine and Albendazole) Therapy for Lymphatic Filariasis in Papua New Guinea
**Study Type:**	Survey to understand community acceptability of LF drugs
**Population:**	Participants aged 14 and over who received 3 drugs (IDA) for the community study<br><br>Participants aged 14 and older who received 2 drugs (DA) for the community study
**Number of sites included in the study:**	Study sites will be communities in:<br><br>Fiji: communities in Gau Island, with possibility of Rotuma Island, if alternative site is needed<br><br>Haiti: Commune of Quartier Morin

APPENDIX 7: TREATMENT ACCEPTABILITY STUDY PROTOCOL

	India: Selected communities in Yadgir district, Karnataka<br><br>Indonesia: Kahale and Karang Inda in Sumba Barat Daya district, Nusa Tenggara Timur Province<br><br>Papua New Guinea: Madang Province, Bogia District and as alternatives, Dreikiker District, East Sepik Province and East New Britain Province
**Duration of Study Participation:**	<2 hours
**Primary objective:**	Assess the overall acceptability of the 3-drug treatment in the community as compared to the 2-drug treatment
**Secondary objectives:**	1. Measure the perception of adverse events (AE) reported by safety trial participants, comparing those in the 2-drug versus 3-drug arms<br>2. Assess the overall acceptability in the community of the 3-drug regimen, as compared to the 2-drug regimen<br>3. Assess the overall acceptability in the community of those individuals who are (microfilaria) mf positive, as compared to those who are mf negative<br>4. Investigate the acceptability and feasibility of delivering the 3-drug regimen
**Incorporation into the DOLF project**	Data will be available and reviewed at a country level and at the project level. This protocol outlines the collection and analysis of the aggregated data from all sites,

As part of the larger "Community Based Safety Study of 2-drug versus 3-drug Therapy for Lymphatic Filariasis" a study to assess treatment acceptability in the community is planned in each research site: Fiji, Papua New Guinea, Indonesia, Haiti, and India. The overall aim of this research is to understand the community's acceptance of the 3-drug regimen as well as gain insight into the feasibility of administering this new therapy in the future. Community acceptance will be measured using a survey to community members receiving treatment during the trial. Part of the investigation will include assessing community member's perception of the possible adverse events experienced as a result of the 3-drug therapy, and how that might affect future rounds of mass drug administration (MDA) at the community level. In addition, focus group discussions (FGD) will be carried out with community members and community health workers to further investigate acceptability of the new therapy. To complement the community survey and focus group discussions, a series of key informant interviews are proposed with community leaders and health personnel in the same communities to assess perceptions about the 3-drug *versus* the 2-drug regimen as well as gain insight into the feasibility of distributing the new regimen as well as perceptions about managing adverse events.

B. Rationale for the study

With the introduction of a new treatment regimen for the elimination of lymphatic filariasis (LF), understanding community perceptions about the treatment, its adverse events (AE) as well as its efficacy will be an important component of assessing the acceptability of the 3-drug therapy. In particular, perceptions about the severity of experienced or observed AE, the efficacy of the treatment in killing the worms and understanding the positive presence of AE will be important to investigate.

Research has demonstrated the important impact that AE can have on individuals' acceptance of LF treatment using the 2-drug regimen (18, 19). In some areas where MDA has been ongoing for many years, we might expect these AE to be objectively of minimal clinical significance, yet subjectively community members continue to report "fear of AE" as a deterrent to comply with MDA. In recent research in a low prevalence area in Indonesia, 33% of individuals interviewed reported experiencing some form of side effect or AE as a result of taking the LF treatment (A. Krentel personal experience). Thomsen et al (2016) reported a higher rate of AE in those who were administered the 3-drug regimen versus those who received the 2-drug therapy (20). As the wider application of this new therapy is considered, it will be important to understand if the perception of these AE is different in between the two treatment arms.

Another important deterrent to compliance with MDA is a lack of understanding of the benefit of treatment (21, 22). The 3-drug regimen has been shown to be highly effective in the reduction of microfilariae (20); therefore communicating this message to participants will be of crucial importance. Measuring participants' understanding of this message will be essential in determining their acceptance of AE associated with the treatment. In PNG and in neighboring Indonesia when communities understand the reasons AE occur, they welcome them as a sign that the drugs are working (23, 24). Knowing if this message also works with the 3-drug therapy where more AE are expected to occur is important in the future promotion of this treatment.

For the purposes of this research, a mixed method approach is recommended, combining the use of a community survey, focus group discussions and in depth interviews with key informants. The community survey will allow a robust comparison of treatment acceptability between those receiving the 2-drug regimen and those receiving the 3-drug regimen. A composite score will measure acceptability, combining outcomes like the respondents' intention to take the treatment again and willingness to recommend it to other family members. Acceptability will be analyzed by the impact of some of the known factors that impact compliance: perception of AE, knowledge about AE, perceptions about the drug characteristics (safe, number of pills, taste), knowledge of vector, belief that the treatment is associated with health, and others. In order to assess the difference between the two treatment arms, the sampling frame for the community survey will take into account which regimen the individual received.

To complement the community surveys and provide further in depth analysis, focus group discussions (FGD) are planned with specific groups in the community, namely men, women, young people and community health workers. The FGDs will provide further insight and depth for some of the questions asked in the community survey. Specifically FGDs will investigate issues expected to relate to the 3-drug regimen: number of pills, perception of AE, how to ensure directly observed treatment and proposed messages to encourage compliance.

These results will be further substantiated by interviews with key community leaders, as well as community and professional health workers working in LF elimination at the village

level. These interviews will provide an understanding of the macro level issues that key informants perceive as critical to consider with the use of the 3-drug therapy. With this, interview respondents will be asked what advantages and concerns they have with regards to the 3-drug regimen based on their participation in and understanding of the safety trial.

The outcome of this research will provide operational recommendations to accompany the safety study. These will inform additional acceptability research if the 3-drug regimen is adopted as global policy. An important outcome will be to determine if there are any real differences in community acceptance of the 3-drug regimen when compared to the standard treatment. If there are any differences, then further investigation may be recommended. In addition, the global programme will need to consider how to adjust the delivery protocols and recommended messages used by community drug distributors giving out the 3-drug regimen. The acceptability study will provide a preliminary understanding of these issues and will provide important insights into the use of this regimen on a wider scale.

C. Study objectives

1) Measure the perception of AE reported by safety trial participants, comparing those in the 2-drug *versus* 3-drug arms
2) Assess the overall acceptability in the community of the 3-drug regimen, as compared to the 2-drug regimen
3) Assess the overall acceptability in the community of those individuals who are mf positive, as compared to those who are mf negative
4) Investigate the acceptability and feasibility of delivering the 3-drug regimen

D. Community survey

Community surveys are often called Knowledge, Attitudes and Practice (KAP) surveys because they use a cross sectional survey design to understand what community members know about disease, treatment and prevention; how they perceive factors related to the disease and finally what they do about it (e.g. take a drug, hang a bednet, use a condom). For the purposes of this survey, it is recommended to use a cross sectional survey design. However the terminology and format of the KAP may not be the most appropriate questionnaire design for the study proposed. Specific knowledge about LF disease is not a strong predictor for compliance in MDA for LF, with the exception of knowing that mosquitoes transmit LF (25, 26). For the purposes of this research, focusing on knowledge of LF disease may not inform community acceptability of the 3-drug regimen as compared to the 2-drug regimen. Furthermore research has shown that there are important intrinsic reasons that affect people's decisions to take or not to take the LF treatment during MDA. Social norms of compliance, emotional cues, altruism and an individual's personal situation have all been shown to be associated with taking the LF drug (22, 27-29). Understanding some of these intrinsic factors associated with taking the 3-drug regimen as opposed to the 2-drug regimen will be important in building a picture of community acceptability. As a result, although there may be similarities in some of the questions asked, it is recommended to call the community survey a "treatment acceptability survey" as opposed to a "KAP survey."

<u>1. Timing</u>

In order to allow some space between the clinical assessment and monitoring of AE in the community trial as well as some time for the effects of ivermectin to become apparent, individuals approached for enrollment in the community acceptability survey within four

months after their enrollment in the safety trial. As the safety trial will be a memorable event in the community, it is expected that the effect of recall bias will be minimal among respondants. (30)

2. Questionnaire development

Questionnaire development is based on previous LF surveys carried out in Indonesia and in Papua New Guinea. In addition, known influences based on the most recent literature on compliance will be included in the acceptability survey, where appropriate. Validated questions from acceptability research as well as quality of life indicators for scabies will be included in the questionnaire.

Questionnaires will be written in English and translated into the local language. In order to test the understandability of the questionnaire with the local population, the enumerators will give advice on the vocabulary used during the training and a small sample of individuals will be administered the questionnaire prior to survey implementation. At the end of this testing, these respondents will be asked to comment on the questions themselves, whether they were clear and the language was appropriate. Changes will be made if needed. The questionnaire will then be translated back into English.

3. Sampling frame

In estimating the sample size for the acceptability survey, one of the challenges we have is that we do not know the estimated acceptability rates in people who have received the 3-drug regimen. From recent research in Indonesia (A. Krentel, personal experience) in low (mf rate=1%) and high prevalence (mf=8%) areas, we know that acceptability with DEC+ALB, as measured in the intent to take the LF drugs again, was measured as 79% and 82% respectively.

Because we do not have a 3-drug acceptability rate, we cannot estimate the difference we might expect in between the regimen groups. As a result, this survey will create preliminary data, estimating the difference in acceptability rates between those individuals receiving the 2 and 3 drug regimens as well as the difference in rates between those with positive mf rates at the start of the safety trial and those who are mf negative. This survey will provide insight into possible trends in acceptability and will inform if further investigation is needed.

The range of mf prevalence is expected to be wide in between the study sites. As such, we will oversample those areas where it will be higher. The target samples for the acceptability survey are as follows:

- 400 individuals in Papua New Guinea
- 400 individuals in Indonesia
- 400 individuals in Haiti
- 450 individuals in Fiji
- 400 individuals in India

Within each country, equal numbers of subjects will be enrolled in the two drug and the three drug intervention arms. In PNG, we will oversample those villages were LF prevalence is expected to be higher. With a projected 50% infection rate, this is the only participating country that is expected to have an infection rate in excess of 10%. It is therefore the only country that will provide a universe of infected subjects that is sufficiently large to provide accurate measures of the acceptability of study medications in subjects who are infected. Specifically, if we add the 50 subjects who are expected to be infected in Indonesia, Haiti, and Fiji combined to the 200 anticipated infected subjects in PNG, we anticipate that this acceptability study will enroll 250 infected subjects, or 125 in each

intervention arm. With a total of 125 infected subjects in each arm, a dichotomous measure (yes or no) of the acceptability of a particular drug combination will, with 95% certainty, differ by no more than 9% from the true acceptability rate.

In Fiji, as there will be three study arms, we expect to enroll 150 individuals per treatment arm. For the aggregated analysis, we will include 300 individuals in total, 150 for those receiving the 3-drug regimen once and those receiving the 2-drug regimen once, as is the protocol in the other countries. The data from the total 450 participants will be interpreted and analyzed at the country level.

Because we expect the acceptability of a particular drug combination to be highly correlated within families, our protocol permits the enrollment of precisely one subject from each participating family. Eligible family members will have to be at least 14 years of age. As chronic manifestations of the disease begin to show at adolescence, so personal experience with LF may also begin at this age (31). Both men and women will be included in the sample.

Enrollment procedures are as follows. Our goal will be to interview acceptability study participants beginning at least two weeks after the AE monitoring has been completed within a given village. The reason for waiting at least two weeks is that we want to minimize the likelihood that we will interview individuals who are still experiencing the symptoms of any adverse events that may have occurred in the parent study. If we were to conduct acceptability interviews while subjects were still experiencing adverse effect symptoms, the immediacy of those symptoms might bias subjects' perceptions of acceptability.

All families within a village that participated in the parent safety study will be eligible to participate in this acceptability study, with consent for such participation having been a part of the consent form of the parent study. Within each country, we will enter villages sequentially in the order that they were entered in the parent study. We will continue to interview subjects in a particular country until the target number within a given study arm has been reached. When the target number has been reached in one study arm, we will stop enrolling subjects in that arm and will continue enrolling in the other study arm until the target has been reached.

The family member to be interviewed for this study will be selected using the following procedures. A list of family members over the age of 14 who have been enrolled in the parent study will be compiled as soon as possible after the parent study has been concluded in a village. From this list, a random family member will be selected as the target participant in the acceptability study. When we approach a household, we will seek to interview the randomly selected family member. If that family member is not immediately available, we will discuss whether the logistics of conducting the interview with that family member at a different time are feasible. If we conclude that waiting for that alternative time is not reasonable or if the selected family member does not wish to participate, we will conduct the interview in whatever family member is available, independent of which individual has been randomly selected.

Enumerators will travel to the house to interview the identified individual. Data will be collected using the REDCap system.

4. Outcome of interest:

APPENDIX 7: TREATMENT ACCEPTABILITY STUDY PROTOCOL

Acceptability of the 3-drug therapy will be measured in a composite score from the following questions[1]:

- Intention to take LF drugs in the future measured on a 5-point scale ranging from "I will never take this drug again" to "I will definitely take this drug again." (Adapted from Liau and Zimet 2001)
- Willingness to encourage other family members to take the LF drug, if offered in the future measured as a 5-point scale ranging from "I will never encourage my family to take the LF drugs" to "I will definitely encourage my family to take the LF drugs."
- Overall feeling about the LF elimination program as a 5-point scale ranging from "Very negative" to "Very positive"
- Perception of health since taking the LF drugs as a 5-point scale ranging from "Considerably worse" to "greatly improved"
- In addition to the scoring, each outcome can be converted to a binary variable for multivariate modeling.

5. Inputs / Exposure variables:

- SES data
- Data from safety trial (clinical presence of AE, mf rate, household information)
- Treatment arm (2-drug versus 3-drug)
- Informed about the treatment before receiving the drug (e.g. did they receive any information)
- Belief in the efficacy of the treatment to eliminate / prevent LF (e.g. believe that the drugs work to prevent / treat LF)
- Belief in the efficacy of the treatment to treat scabies (e.g. believe that the drugs work to treat scabies)
- Belief in the efficacy of the treatment to treat other intestinal worms (e.g. believe that the drugs work to treat worms)
- Knowledge of the 'positive' component of AE (e.g. occur because the medicine is working)
- Perception of AE (e.g. none, mild, moderate, severe)
- Understanding that taking LF medicine is good for promoting health
- Knowledge that mosquitoes transmit LF
- Perception that the rest of the family/ household would take the LF drugs, if offered in the future (yes/no)
- Belief that the drug distributors are doing a good job (using a 10-point scale)
- Perceptions of the drugs (e.g. safe, neutral, dangerous)
- Components of the drugs (e.g. number, size, taste of pills)
- Emotions surrounding LF treatment (e.g. how does taking LF treatment make you feel?)

6. Analysis

For the data cleaning and data reduction, the following steps will be performed:

- Check response bias
- Clean the raw data set (range check, consistency checks)
- Transfer corrected data set to STATA statistical software (Stata Corporation, College Station, Texas).

---

[1] Scoring concept amended from Treatment Acceptability Research (see Carter SL (2007) "Review of Recent Treatment Acceptability Research" *Education and Training in Developmental Disabilities* 42(3): 301-316.

- Group continuous variables into categorical variables, namely age. Recode certain variables where needed.

For the analysis, a descriptive analysis of the whole dataset will be prepared. The data from the community survey will be linked to the safety trial within the REDCap system.

Likert scales will be analyzed as both dichotomous and as continuous variables.

For both of the predictors of acceptability (drug regimen and presence of mf) logistic regression models will be created. Presence of AE as measured in the clinical surveys will be considered in the analysis, as will subjective perceptions of AE.

E. Focus Group Discussion

1. Timing

The focus group discussions will take place at the same time as the community survey, in the same communities.

2. Sampling frame

For the focus group discussions, we will identify persons from specific groups of people: women of reproductive age, young people, men and community health workers. The rationale behind the selection of each of these groups is related to the prevailing evidence of their participation in MDA in the literature. Women of reproductive age often do not comply with treatment because they are either pregnant or breastfeeding, however they are often the gatekeepers for health in the household and ensure members of their household takes the treatment when offered. Men and young people have been known to be less compliant with MDA and so understanding their perceptions about the 3-drug regimen, MDA in general and soliciting their advice about how best to promote and reach their communities will be informative. Finally, as community health workers are usually the persons responsible for distributing the drug at the community level, understanding their perspectives on DOT, AE and messaging for the 3-drug regimen is important.

For the FGD, women, young men and men will be selected from the cohort of individuals receiving the 3-drug regimen.

3. Development of the topic guide

Range of issues to explore include:

- How is LF elimination different / similar from the other health programs in their village?
- What are the health benefits from taking the treatment?
- What are the social benefits from taking the treatment?
- Do people like to take the pills in front of the distributor? Why or why not?
- How do you feel about the number of pills that you have to take?
- Why don’t people want to take it?
- Did you have any side effects after you took the drugs (positive or negative)? How did you feel about them?
- What suggestions do you have to promote MDA to their community? Household?
- Are there any specific messages you would recommend to us?

4. Analysis of the FGD

Recorded focus group discussions will be transcribed word for word in the local language. They will be translated into English. A second researcher with knowledge of English and the local language will check translation, sampling portions of each transcript and back translating them from English to the local language to check the reliability of the translation.

The researchers will read through each transcript, recording emergent themes in an Excel matrix. NVivo will be used to assess trends and patterns in the interview transcripts.

F. In depth interviews with key informants

1. Timing

The key informant interviews will take place at the same time as the community survey, in the same communities.

2. Sampling frame

A purposive sampling frame will be used, with individuals identified based on their leadership and cultural position with the village as well as their involvement with LF elimination and with the community trial. With this in mind, a range of 8-10 individuals will be included in the sample. In order to understand the acceptability of administering the 3-drug regimen, individuals to be interviewed would need to be those persons who are either directly involved with LF activities in the village or who *would be* involved in MDA in the future. Suggestions include community and/or religious leaders, community health workers, teachers.

3. Development of the topic guide

Range of issues to explore include:

- What are the advantages of the 3-drug therapy in MDA? Disadvantages?
- What opportunities do they see in the administration of the 3-drug therapy, versus the 2-drug therapy?
- What concerns or challenges do they see in the administration of the 3-drug therapy, versus the 2-drug therapy?
- How do they feel about the number of pills that the community is asked to take?
- How do they feel about the side effects people might have / have?
- What suggestions do they have to promote MDA in this village? This province? The country? What messages would they recommend using?
- Which groups of people do they think will be difficult to reach with future MDA? Why? Any advice to approach them?

4. Analysis of the IDIs

Recorded interviews will be transcribed word for word in the local language. They will be translated into English. A second researcher with knowledge of English and the local language will check translation, sampling portions of each transcript and back translating them from English to the local language to check the reliability of the translation. The researcher will read through each transcript, recording emergent themes in an Excel matrix. Nvivo will be used to assess trends and patterns in the interview transcripts.

G. Ethical Considerations

In the parent safety trial informed consent form in each country, the participant is notified that they may be contacted for a follow-up survey so each participant will have agreed to be identified for the purposes of the acceptability study during the informed consent process for the safety trial.

1. Community survey

Ethical approval will be obtained from the local national research institution in each country as well as Washington University in St. Louis, Case Western University, Royal Children's Hopsital Melbourne and Bruyère Research Institute.

Prior to giving consent to participate, the enumerator will read out the information sheet in the local language containing the aim of the survey, the length of time it is expected to

take (15 minutes) as well as the protection of confidentiality for each respondent. Following this, each respondent will be asked to sign the informed consent form and where respondents are illiterate, a mark can be made. The enumerator will indicate that informed consent has been given. Age of eligible respondents is 14 years of age and older. For those aged 14 – 18 years, parental consent will be sought and provided on the informed consent form before the interview can begin. All forms will remain with the research team and will not contain any personal information other than the individual's signature.

At the end of the interview, each respondent will be given an information sheet with the principal investigator's contact details, should there be any questions. With this sheet, the respondent will also receive a brief information sheet on lymphatic filariasis, the mass drug administration and who is eligible for treatment.

The data will be stored on Washington University servers during the duration of the study. After the study ends, electronic copies of the de-identified datasets will be kept by the PI indefinitely.

<u>2. Focus Group discussions</u>
Ethical approval will be obtained from the local national research institution in each country as well as Washington University in St. Louis, Case Western University, Royal Children's Hospital Melbourne and Bruyère Research Institute.

The interviewer will read the informed consent form to each person participating in the focus group discussion. The respondents will be asked to each sign an informed consent form for their participation. All interviews will be recorded with the permission of the respondent. Where permission is not granted, the interviewer will ask to take notes throughout the interview.

Any identifying information (name, address) will not be recorded. Individuals will not be identified in the transcripts or in the recordings and their anonymity will be maintained in all reporting and in the manuscripts. Transcripts of the interviews will remain with the research team.

The data will be stored with the PI, under password protection. After the study ends, electronic copies of the de-identified datasets will be kept by the PI indefinitely.

<u>3. In depth interviews with key informants</u>
Ethical approval will be obtained from the local national research institution in each country as well as Washington University in St. Louis, Case Western University, Royal Children's Hospital Melbourne, and Bruyère Research Institute.

The interviewer will read the informed consent form to each person participating in the interview. The respondents will be asked to sign an informed consent form for their participation. All interviews will be recorded with the permission of the respondent. Where permission is not granted, the interviewer will ask to take notes throughout the interview.

Any identifying information (name, address) will not be recorded and the identity of the respondent will be kept confidential in reporting. Transcripts of the interviews will remain with the research team. The data will be stored with the PI under password protection. After the study ends, electronic copies of the de-identified datasets will be kept by the PI indefinitely.

H. Fiji Specific Inclusions
Fiji has unique components of the Safety study which allow for further evaluation of the effects of triple therapy on scabies and soil transmitted helminths (STH). The key

difference is that half of the 3 drug villages will receive a second dose of ivermectin on Day 8. As such, the Acceptability Study in Fiji will evaluate how the overall acceptability compares between those with and without scabies, between those with and without STH and between the 3 drug treatment (one dose of ivermectin) and the 3 drug treatment (two doses of ivermectin).

In addition to previously mentioned translational outcomes from the Acceptability Study, the Fiji specific components outlined will influence global decision making around a 2nd dose of ivermectin for scabies.

To assist in the evaluation questions have been added to the FGD and IDI guides as follows:

“This special distribution of LF treatment also treated two other conditions. Do you know what they were?

If they knew the answer (scabies and intestinal worms), then ask:
Did this change how likely you were to take the treatment?

If they didn’t know the answer, tell them the other 2 conditions were scabies and intestinal worms. Then ask:
If you knew the treatment also covered these diseases, would it have changed how likely you were to take the treatment?”

APPENDIX 7: TREATMENT ACCEPTABILITY STUDY PROTOCOL

I. Information sheet and informed consent for community survey
(to be read to each respondent)

Good morning / afternoon, my name is _________. I am working with ______. We are doing a survey about the recent special distribution of LF drugs in your community. Your opinion is important to improve the health of the people living in this community and we thank you for your time.

As part of the "Community Based Safety Study of 2-drug versus 3-drug Therapy for Lymphatic Filariasis" that just happened in your area last month, we are asking some people who participated in that study to take part in a short survey so that we can understand more about lymphatic filariasis [or local name], the drugs used in the safety trial and health in general. Your name was selected randomly from the list of people who participated in that special distribution.

It is important that you understand why we are doing this survey, so please read this information sheet or listen carefully as I read it. If you have any more questions, please ask me (the interviewer) and I will try to answer them for you.

We are interested in the experiences people had participating in the special distribution for LF and what people in your community understand about lymphatic filariasis [or local name]. We would like to talk to about 400 people in this area so that we can understand better how people felt about taking the LF drugs. Your participation is entirely voluntary and you are under no obligation to participate. Whether or not you choose to participate, your status and access to health care will not be affected in any way. There are no anticipated risks or benefits for you if you choose to participate in this survey.

If you do choose to help with this study, we will only need about 20 minutes of your time to ask you some questions. At any time during this discussion, you are free to stop and withdraw from the study. You do not have to give me (the interviewer) a reason.

During the interview, I will be writing down your responses on this paper. We will not record your name during our discussion today and you will be assigned an identification number for our records. The information that you provide during our discussion will be completely confidential. All files will remain with the main investigator and will be kept in a locked cabinet and all electronic files will be password protected. The main research team in this country from _______(Institute name) will access the study files as will researchers from Washington University in St. Louis and the Bruyère Research Institute in Canada, and their ethics boards.

I have read the information sheet provided or it has been read to me concerning this study and I understand what will be required of me if I participate in this study, which will be a verbal interview and discussion.

My questions regarding this study have been answered by: _________________.

I understand that at any time I may withdraw from this study without giving a reason and without having any effect on my access to health care.

I agree to take part in this study.

Signature of the respondent: _______________________________________

Signature of parent/ guardian if respondent under 18 years: ______________________________

Signature of a witness: ____

Signature of the enumerator to indicate that the informed consent has been read and the information sheet given to the respondent: ___________________________________

APPENDIX 7: TREATMENT ACCEPTABILITY STUDY PROTOCOL

J: Information sheet and informed consent for in depth interviews with key informant

As part of the "Community Based Safety Study of 2-drug versus 3-drug Therapy for Lymphatic Filariasis" that just happened in your area last month, we are asking some people who participated in that study to take part in a verbal discussion so that we can understand more about lymphatic filariasis [or local name], the drugs used in the safety trial and health in general. It is important that you understand why we are doing this survey, so please read this information sheet carefully. If you have any more questions, ask the interviewer and they will try to answer them for you.

We are interested in the experiences people had participating in the safety trial and what they understand about lymphatic filariasis [or local name]. We would like to talk to about 8 people in this area so that we can understand better how people felt about taking the LF drugs. Your participation is entirely voluntary and you are under no obligation to participate. Whether or not you choose to participate, your status and access to health care will not be affected in any way. There are no anticipated risks or benefits to participation in this study.

If you do choose to help with this study, we will only need about one hour of your time to ask you some questions and to discuss informally. At any time during this discussion, you are free to stop and withdraw from the study. You may refuse to answer any of the questions. You do not have to give the interviewer a reason.

The information that you provide during our discussion will be completely confidential and we will not even write down your name or address. We will take some written notes during our discussion and if you agree, we may also record the interview using a digital recorder so that it will be easier to remember what we discussed. All digital files will remain with the main investigator and your name and address will not be recorded. We will write down the conversation and store it safely, with a password. Other researchers may ask to look at our discussion together, and we may share it with them, provided that they respect the same rules of confidentiality. Study files may also be accessed by researchers at ________ [local institution], Washington University in St. Louis and the Bruyere Research Institute in Canada and their ethics boards.

I have read the information sheet provided or it has been read to me concerning this study and I understand what will be required of me if I participate in this study, which will be a verbal interview and discussion.

My questions regarding this study have been answered by: _________________.

I understand that at any time I may withdraw from this study without giving a reason and without having any effect on my access to health care.

I agree to take part in this study.

Signature of the respondent: ______________________________________

Signature of a witness: ____

Signature of the enumerator to indicate that the informed consent has been read and the information sheet given to the respondent: __________________________________

APPENDIX 7: TREATMENT ACCEPTABILITY STUDY PROTOCOL

K: Information sheet and informed consent for focus group discussion participants

As part of the "Community Based Safety Study of 2-drug versus 3-drug Therapy for Lymphatic Filariasis" that just happened in your area last month, we are asking some people who participated in that study to take part in a focus group discussion so that we can understand more about lymphatic filariasis [or local name], the drugs used in the safety trial and health in general. It is important that you understand why we are doing this survey, so please read this information sheet carefully. If you have any more questions, ask the interviewer and they will try to answer them for you.

We are interested in the experiences people had participating in the safety trial and what they understand about lymphatic filariasis [or local name]. We would like to talk to about 4 groups of people in this area so that we can understand better how people felt about taking the LF drugs. Your participation is entirely voluntary and you are under no obligation to participate. Whether or not you choose to participate, your status and access to health care will not be affected in any way. There are no anticipated benefits to participation in this study.

If you do choose to help with this study, we will only need about one hour of your time to ask you some questions and to discuss informally. At any time during this discussion, you are free to stop and withdraw from the study. You may also refuse to answer any questions. You do not have to give the interviewer a reason.

We request that participants do not share what is discussed during the focus group with others; however there is the risk that confidentiality will not be maintained. The interviewers will maintain confidentiality in the records by not recording the names of the focus group participants. We will take some written notes during our discussion and if you agree, we may also record the interview using a digital recorder so that it will be easier to remember what we discussed. All digital files will remain with the main investigator and your name and address will not be recorded. We will write down the conversation and store it safely, with a password. Other researchers may ask to look at our discussion together, and we may share it with them, provided that they respect the same rules of confidentiality. Study files may also be accessed by researchers at [local research institution], Washington University at St. Louis and the Bruyere Research Institute in Canada and their ethics boards.

I have read the information sheet provided or it has been read to me concerning this study and I understand what will be required of me if I participate in this study, which will be a verbal interview and group discussion.

My questions regarding this study have been answered by: _________________.

I understand that at any time I may withdraw from this study without giving a reason and without having any effect on my access to health care.

I agree to take part in this study.

Signature of the respondent: ______________________________________

Signature of a witness: ____

Signature of the enumerator to indicate that the informed consent has been read and the information sheet given to the respondent: __________________________________

APPENDIX 7: TREATMENT ACCEPTABILITY STUDY PROTOCOL

L: ACCEPTABILITY SURVEY AND TOPIC GUIDES FOR FGD AND IDI

Final versions of the Acceptability Survey and Topic Guides for the FGD and IDI are submitted in separate documents titled "FIT_Acceptability_Survey_v4.0_17-04-21" and "FIT_Acceptability_Topic_Guides_v4.0_17-04-21".
