# Supplementary material for: Community control strategies for scabies: A cluster randomised noninferiority trial
Source: PLoS Med. 2021 Nov 10;18(11):e1003849. doi: 10.1371/journal.pmed.1003849 (PMC8612541; doi:10.1371/journal.pmed.1003849)
Supplement: S1 Methods — (PDF) [file pmed.1003849.s011.pdf]

Supporting information file for *Community control strategies for scabies: a cluster randomised non-inferiority trial*

S2 Methods. Sample size calculation

The Monte Carlo simulation framework employed repeated sampling from independent Binomial distributions $B_j(n, p(group))$, each representing the anticipated cluster size n and the anticipated prevalence $p(group)$ within a specific cluster j. The target parameter $p(group)$ was set to depend on the respective study exposure level i.e. $group \in \{SAT, IVM\text{-}1, IVM\text{-}2\}$.

For the anticipated number of clusters $j=1,\ldots,24$ a balanced random allocation of exposure levels was implied i.e. 8 clusters per *group*. To induce intracluster correlation, within-cluster prevalence parameters were sampled from respective uniform distributions generating a conservative natural variation of $p(group)\pm5\%$. For example, baseline prevalence was set to range between 25% and 35% across clusters, inducing an intracluster correlation between 0.03 and 0.06 adopting the Fleiss-Cuzick estimator for the ICC [1].

For a grid of values of potential cluster sizes n and the presumed parameter values $p(group)$, data simulations were repeated 1000 times and respective contrasts in group-level prevalence values were computed along with 95% Bonferroni-adjusted one-sided confidence intervals. If at least 80% of the computed intervals across the 1000 simulation runs demonstrated group differences in scabies prevalence, the respective cluster size n was deemed to provide sufficient statistical power.

1. Wu S, Crespi CM, Wong WK. Comparison of methods for estimating the intraclass correlation coefficient for binary responses in cancer prevention cluster randomized trials. Contemp Clin Trials. 2012;33(5):869-80. Epub 2012/05/26. doi: 10.1016/j.cct.2012.05.004. PubMed PMID: 22627076; PubMed Central PMCID: PMCPMC3426610.
