# Supplementary material for: Community control strategies for scabies: A cluster randomised noninferiority trial
Source: PLoS Med. 2021 Nov 10;18(11):e1003849. doi: 10.1371/journal.pmed.1003849 (PMC8612541; doi:10.1371/journal.pmed.1003849)
Supplement: Attachment — Submitted filename: PMEDICINE-D-21-02600_Response_to_Reviewers.docx [file pmed.1003849.s013.docx]

Response to reviewers

Requests from the editors

1. You mention in the article metadata that all data are included in the ms and supplementary files - are you able to include anonymized patient-level data in excel files or similar?

We have added an additional supplementary file “S1 Data” with anonymized individual level data including study identification number, sex, island, and baseline age, village number, treatment group, scabies, impetigo, and at 12 months age, village number with respective treatment group allocation at baseline, scabies and impetigo.

#### 2. PLOS Medicine style is to use "sex" rather than "gender", where appropriate.

All instances are to PLOS Medicine style.

#### 3. Please quote aggregate demographic details for participants in the abstract; along with some key details of villages/clusters.

Added details to abstract as follows:

Line 45: “All residents from 35 villages on two Fijian islands were eligible to participate.”

Lines 53-58: “We enrolled 3812 participants at baseline (July to November 2017) from the 35 villages with median village size of 108 (range 18-298). Age and sex of participants were representative of the population with 51.6% male and median age of 25 years (interquartile range 10-47). We enrolled 3898 at 12 months (July to November 2018).”

#### 4. In the abstract and throughout the paper, please quote p values alongside 95% CI, where available.

We have added p-values to risk difference as suggested by Reviewer 5. Treatment group prevalence 95% CIs do not have p-values associated with them as we were not testing a hypothesis. Since our objective was to assess non-inferiority of absolute reduction between treatment groups, confidence intervals were used instead of p-values, which are normally applied to a superiority hypothesis.

#### 5. Please combine the "Methods" and "Findings" subsections of your abstract.

Combined as suggested.

#### 6. The final sentence of the new combined subsection should begin "Study limitations include ..." or similar, and should quote 2-3 of the study's main limitations.

We had added details as follows, lines 63-65:

“Limitations of the study included the method of scabies diagnosis by non-experts, a lower baseline prevalence than anticipated, and the addition of diethylcarbamazine and albendazole to scabies treatment.”

#### 7. Please remove the information on funding from the abstract. In the event of publication, this information will appear in the article metadata, via entries in the submission form.

We have removed this paragraph as suggested.

#### 8. Please restructure the "Author summary" so that each of the three subsections consists of around 3 bulleted points, each consisting of no more than 1 or 2 short sentences.

We have restructured the “Author summary” as suggested, lines 87-119.

#### 9. Please remove the "Role of the funding source" section from the Methods.

We have removed this section as suggested.

#### 10. Please avoid claims such as "the first", in the final paragraph for example, and where needed add "to our knowledge" or similar.

Corrected as follows:

Lines 99-100: “We found one-dose ivermectin-based MDA was non-inferior to two-dose for reducing scabies prevalence at 12 months.”

Lines 383-386: “However, these previous studies used a two-dose ivermectin-based MDA approach, and our study is the first randomised trial to our knowledge to demonstrate that community wide treatment with a single dose is able to substantially reduce both burden and transmission, despite ivermectin having minimal ovicidal activity.”

Lines 435-437: “Our study provides evidence from a randomised trial that one-dose of ivermectin-based MDA is non-inferior to two-dose, and therefore may be adequate as a strategy for controlling scabies in endemic settings.”

#### 11. Throughout the text, please locate reference call-outs prior to punctuation and remove spaces from within the square brackets (e.g., "... over 26,000 [7,8].").

Corrected.

#### 12. Please confirm that the map in fig. 1 can be published under a CC BY licence, in the event of publication.

The map can be published under a CC BY licence.

Reviewer #1

Community control strategies for scabies: a cluster randomised non-inferiority trial

This is an important report on a cluster randomised trial for prevention and control of scabies. The authors do make a case for the scientific gap is whether a one-dose ivermectin-based mass drug administration (MDA) would not be inferior to a two-dose ivermectin-based MDA. So the authors designed an open-label cluster RCT with 3 arms:
- (IVM-2) Ivermectin-based treatment plus DEC and albendazole followed by a second dose of ivermectin 8 days later
- (IVM-1) Ivermectin-base treatment plus DEC and albendazole
- (SAT) Clinical screen for scabies and treatment (offered as well DEC and albendazole)

General comments

#### 1. The primary outcome in this trial is absolute reduction of prevalence from baseline to 12 months. OK to use the IVM-2 as the reference group.

#### a) The sample size calculation was done under the assumption of higher baseline prevalence and larger absolute reductions. This deserves note and more discussion. In fact the results section almost obscures this because it focuses more on the secondary outcome see below.

We have added the following, lines 413-415:

“Fourth, the baseline scabies prevalence in our study was lower than anticipated, likely due to geographical variation in prevalence, but this did not impact the statistical power of the trial.”

#### b) Please add more notes on how the simulation for sample size calculation was done.

The Monte Carlo simulation framework employed repeated sampling from independent Binomial distributions *B_j_(n, p(group))*, each representing the anticipated cluster size *n* and the anticipated prevalence *p(group)* within a specific cluster *j.* The target parameter *p(group)* was set to depend on the respective study exposure level i.e. *group* *∈ {SAT, IVM-1, IVM-2}*.

For the anticipated number of clusters *j=1,…,24* a balanced random allocation of exposure levels was implied i.e. 8 clusters per *group*. To induce intracluster correlation, within-cluster prevalence parameters were sampled from respective uniform distributions generating a conservative natural variation of *p(group)±5%.* For example, baseline prevalence was set to range between 25% and 35% across clusters, inducing an intracluster correlation between 0.03 and 0.06 adopting the Fleiss-Cuzick estimator for the ICC[1].

For a grid of values of potential cluster sizes *n* and the presumed parameter values *p(group),* data simulations were repeated 1000 times and respective contrasts in group-level prevalence values were computed along with 95% Bonferroni-adjusted one-sided confidence intervals. If at least 80% of the computed intervals across the 1000 simulation runs demonstrated group differences in scabies prevalence, the respective cluster size *n* was deemed to provide sufficient statistical power.

We have added this additional information as supplementary file: S1 Methods, and to the manuscript as follows lines 254-263:

“We assumed an estimated scabies prevalence range of 25 to 35% across clusters, based on previous Fijian surveys [4,5], inducing an intracluster correlation (ICC) between 0.03-0.06 adopting the Fleiss-Cuzick estimator for ICC (S1 Methods) [18]. We assumed an absolute reduction of scabies of 29% in IVM-2 group, 22% in IVM-1 group and 15% in the SAT group, based on findings from SHIFT [5]. Utilising a Monte Carlo simulation tool with 1000 simulations and Bonferroni-adjusted one-sided confidence intervals (CI) that maintain a global type I error of 5%, we estimated 24 clusters of 100 with balanced random allocation of exposure, would be sufficient to achieve at least 80% power for comparison of treatment effect between any of the two groups.”

#### c) Please add information on how cluster correction/adjustment was done (on table 2). The lines 213 to 218 do not explain this.

For absolute reduction the clustering is described in the Methods lines 274-277 as follows:

“To calculate the absolute reduction in prevalence between the two timepoints, the 12-month prevalence was subtracted from the baseline prevalence for every village, before calculating the mean of the difference for each treatment group.”

For the proportions at baseline and 12 months we have added the following details, lines 266-274:

“Overall point prevalence estimates with 95% confidence intervals (CI) at baseline and 12 months were calculated, taking account of clustering by village using Taylor series linearization of complex sample variance and stratification by island.”

#### 2. For the secondary outcome [scabies/impetigo prevalence at 12 months]. The authors state that they used "risk differences". However, the lines 222 and 223 say that they use a GLM regression model with binomial family and log-link. This model is also known as log-binomial in the literature and is typically used to obtain relative risks (or proportion ratios), a clear relative measure of association. I would suggest the authors change the terminology from "risk differences" to "prevalence ratio" (or "proportion ratio" etc...)

Apologies, this was our mistake. We used identity link and not log link. We have corrected as follows lines 280-283:

“Risk differences of scabies prevalence at 12 months between any two groups, were calculated using generalised linear models with binomial distribution and identity link that adjusted for clustering by village and stratification by island.”

#### 3. Please see more comments on the CONSORT checklist.

## CONSORT checklist

### 1)    Outcomes (Item 6a)

#### Completely defined pre-specified primary outcome measure including how and when it was assessed.

#### R: Yes it is clearly defined. Lines 203 and 206. Absolute reduction in community prevalence of scabies between baseline and 12 months. This is in percentage.

No response required.

### 2)    Sample size (Item 7a)

#### How sample size was determined

#### R: Between lines 207 and 210 there is an explanation of how the sample size was computed. Expected effectiveness is collected from the literature review. A simulation procedure of 1000 confidence intervals with Bonferroni correction was used to determine the sample size of 24 clusters. However, I cannot find further details of this approach in the attached protocol. They assumed a power of 80% (β = 20%) and α error level of 5%. Another key assumption was that the scabies prevalence would be between 25 to 35% (their results show that overall baseline prevalence ranged between 11.7 to 15.2%, few communities/clusters reached above 25%).

Please see response to General Comments 1b above.

The sample size (power) calculations assumed a higher than actual observed prevalence of the primary proportion parameter (scabies prevalence). However, statistical power is inversely proportional to the assumed prevalence and reaches its minimum at a prevalence value of 50%. Our assumed prevalence of 25% to 35 was therefore a conservative assumption and the actual statistical power was, given the lower observed prevalence, even larger than the anticipated 80%.

### 3)    Sequence generation (Item 8a)

#### Method used to generate random allocation sequence

#### Does the description make it clear if the "assigned intervention is determined by a chance process and cannot be predicted"?

#### R: Yes. Lines 171 to 173. An independent statistician generated the allocation using Stata.

No response required.

### 4)    Allocation concealment (Item 9)

#### Mechanism used to implement random allocation sequence (such as sequentially numbered containers), describing any steps taken to conceal the sequence until interventions were assigned

#### Is it clear how the care provider enrolling participants was made ignorant of the next assignment in the sequence (different from blinding)? Possible methods can rely on centralised or "third-party" assignment (i.e., use of a central telephone randomisation system, automated assignment system, sealed containers).

#### R: The authors state there was allocation concealment in line 173.

No response required.

### 5)    Blinding (Item 11a)

#### If done, who was blinded after assignment to interventions (for example, participants, care providers, those assessing outcomes)

#### Is it clear if (1) healthcare providers, (2) patients, and (3) outcome assessors are blinded to the intervention? General terms such as "double-blind" without further specifications should be avoided.

#### R: There was no blind. This is said in line 174.

No response required.

### 6)    Outcomes and estimation (Item 17a/b)

#### For the primary outcome, results for each group, and the estimated effect size and its precision (such as 95% confidence intervals)

#### Is the estimated effect size and its precision (such as standard deviation or 95% confidence intervals) for each treatment arm reported? When the primary outcome is binary, both the relative effect (risk ratio, relative risk) or odds ratio) and the absolute effect (risk difference) should be reported with confidence intervals.

#### R: For the primary outcome (absolute reduction of scabies or impetigo prevalence from baseline to 12 months follow up, reported on table 2) no measure of association is presented. The reporting text is a bit unclear on this but it is implicit (according to how the sample size was estimated) that the intention is to check the overlapping of the 95% confidence interval. Per arm baseline and 12 month prevalence are presented and differences corrected for clustering. However, is not described in the manuscript how this adjustment/correction per clustering was done for this particular analysis (that lead to table 2).

Please see response to General Comments 1b and 1c above.

### 7)    Harms (Items 19)

#### All important harms or unintended effects in each group

#### Is the number of affected persons in each group, the severity grade (if relevant) and the absolute risk (e.g. frequency of incidence) reported? Are the number of serious, life threatening events and deaths reported? If no adverse event occurred this should be clearly stated.

#### R: This was not reported and I do not think this is relevant for this cluster RCT.

In response to Reviewers 2 and 5, we have added an additional section in the Methods as follows lines 287-291:

“The broader trial included evaluation of the safety of ivermectin, diethylcarbamazine and albendazole compared to diethylcarbamazine and albendazole, as well as the impact of these treatments on lymphatic filariasis at 12 and 24 months, which have been reported previously [13,14]. In addition, the effect on soil-transmitted helminths at 12 months and the acceptability of MDA in Fiji will be reported separately.”

### 8)    Registration (Item 23)

Registration number and name of trial registry

Is the registry and the registration number reported? If the trial was not registered, it should be explained why.

R: The trial was registered prospectively lines 51 and 52. Clinitrials.gov NCT03177993 and ANZCTR N12617000738325.

No response required.

### 9)    Protocol (Item 24)

#### Where trial protocol can be accessed

#### Is it stated where the trial protocol can be assessed (e.g. published, supplementary file, repository, directly from author, confidential and therefore not available)?

#### R: The protocol is added as supplementary material.

No response required.

### 10)    Funding (Item 25)

#### Sources of funding and other support (such as supply of drugs) and role of funders

#### Are (1) the funding sources, and (2) the role of the funder(s) described?

#### R: There is a section declaring the funding sources (lines 66 to 73, only in the abstract). And there is a section for the role of funding source (lines 229 to 232).

These sections have been removed from the submitted manuscript as per the Editor’s instructions.

Reviewer #2

#### Scabies is an important problem and this will be an excellent addition to the literature on its control. The study design is simple and appropriate. It appears that the work was sound. The paper is concise and clear.

#### I have no major comments and only a few very minor ones for the authors' consideration.

#### 1. Line 153: Having "residing in 35 villages" at the end of this sentence seems odd. Suggest add to the front of the sentence, which would then read, "The trial was conducted in 2017 and 2018 in 35 villages…"

We have amended the sentence as follows lines 189-190:

“The trial was conducted in 2017 and 2018 in 35 villages on Rotuma and Gau, two remote islands within the Eastern Division of Fiji (Fig 1).”

#### 2. Line 237, Table 1 and elsewhere: suggest replace "sex" with "gender", which is more likely to be what was recorded by field teams.

As per the Editor’s comments we have continued to use “sex” where applicable in the manuscript.

#### 3. Line 257: I am not sure how to reconcile these two sentences, which at first reading seem to be inconsistent: "Treatment coverage in the IVM-2 group was 82.1% for at least one dose and 80.2% for two doses. Two doses of ivermectin were given to 1170 (87.5% enrolled) and two doses of permethrin to 128 (9.6%) participants." Are the authors using the resident population as the denominator in the first sentence and consenting participants in the second sentence?

We have amended the paragraph to improve clarity and distinction between resident population treatment coverage and enrolled treatment coverage, as follows lines 325-335.

“Treatment coverage of the recorded resident population in the IVM-2 group was 82.1% for at least one dose and 80.2% for two doses. Two doses of ivermectin were given to 1170 (87.5% of enrolled participants) and two doses of permethrin to 128 (9.6%) participants. In the IVM-1 group, resident population treatment coverage was 85.9%. In this group, ivermectin was given to 1079 participants (91.3% of enrolled participants) and permethrin to 103 (8.7%, S2 Table). In the SAT group, there were 176 people found to have scabies with 340 un-infected household contacts, resulting in 516 participants receiving permethrin treatment (resident population treatment coverage of 31.9%, corresponding to 39.9% of enrolled participants, Fig 2).”

#### 4. Line 267: suggest add "aged" in front of "less" in the sentence, "Children less than 15 years…"

Corrected as suggested, line 338.

#### 5. Line 295: "Of the 82 participants with impetigo at baseline, 62 (75.6%) had concurrent scabies, representing a population risk of impetigo attributable to scabies of 72.7% (95% CI 61.8-83.7, p<0.0001). At 12 months, 9 of 27 cases of impetigo (33.3%) had scabies, representing a population risk of impetigo attributable to scabies of 28.6% (95% CI 7.7-49.4, p=0.007)." Suggest just use two significant digits here, since in my view that's all that's justifiable given the data.

In response to Reviewer 5 point 7 below, we have removed the p-values from this paragraph.

#### 6. Line 320: "This approach would be labour-intensive and expensive, requiring a large workforce of highly skilled clinical examiners to screen all individuals within a population." I agree with this statement. It would be made more powerfully if the authors could add either here or in the methods at line 178 some more detail of how the examiners in the study were trained and assessed for competency.

We have added further detail to the Methods as follows, lines 222-224:

“Both nurses were recruited from the Dermatology Hospital in Fiji and completed half-day, classroom-based training in the clinical diagnosis of scabies. Supervision was provided by a paediatrician during the initial fieldwork at both timepoints.”

As well as adding to the Discussion, lines 407-409:

“Non-experts have been reported to be less sensitive in the diagnosis of mild cases of scabies compared to experts [21], and so it is possible that a proportion of cases of scabies may have been missed.”

#### 7. Line 324: "approaching that of an MDA strategy": suggest simplify to "approaching that of MDA".

Corrected as suggested, line 403.

Reviewer #3

#### Review comments

#### PMEDICINE-D-21-02600R1

#### Community control strategies for scabies: a cluster randomised non-inferiority trial

General comments

#### Scabies is a neglected tropical disease that is endemic in many parts of the world. The WHO Road Map for NTDs 2030 has set targets for the control/ eradication of scabies and this calls for renewed efforts and approaches to control. Optimisation of MDA approaches for scabies treatment is therefore an essential tool to aid the attainments of the targets set out in the road map. The authors are therefore to be commended for undertaking this important study.

#### The authors performed a cluster randomised, non-inferiority, open-label, three group unblinded study to compare the effectiveness of 3 control strategies on community prevalence of scabies on 2 Fijian islands 12 months after the intervention. The rationale for the study is clearly set out in the manuscript. The authors are to be commended for undertaking this important work which enables us answer some key questions on scabies control.

#### The manuscript is very well written.

#### The methodology is clear and results are very well presented. the findings and their public health implications have been well discussed

#### The manuscript has been reported in conformance with the CONSORT checklist. The main concern is regarding adverse events. No account is given regarding adverse events (if any). This is especially important as the trial was nested with co-administration with DEC and albendazole for LF. It is important to make some comment on the safety of this co-administered regimen

We have mentioned in the Introduction (lines 161-164) that this trial included safety and lymphatic filariasis efficacy outcomes as reported elsewhere. Since the safety data for this co-administered therapy has been previously published, we agree with Reviewer 1 above that these results were not relevant to this report.

In response to Reviewer 5 we have also added the following to the Methods, lines 287-291:

“The broader trial included evaluation of the safety of ivermectin, diethylcarbamazine and albendazole compared to diethylcarbamazine and albendazole, as well as the impact of these treatments on lymphatic filariasis at 12 and 24 months, which have been reported previously [13,14]. In addition, the effect on soil-transmitted helminths at 12 months and the acceptability of MDA in Fiji will be reported separately.”

CONSORT checklist

1)    Outcomes (Item 6a)

#### Completely defined pre-specified primary outcome measure including how and when it was assessed

#### Is it clear (1) what the primary outcome is (usually the one used in the sample size calculation), (2) how it was measured (if relevant; e.g. which score used), (3) at what time point, and (4) what the analysis metric was (e.g. change from baseline, final value)?

#### R: Well done

No response required.

2)    Sample size (Item 7a)

#### How sample size was determined

#### Is there a clear description of how the sample size was determined, including (1) the estimated outcomes in each group; (2) the α (type I) error level; (3) the statistical power (or the β (type II) error level); and (4) for continuous outcomes, the standard deviation of the measurements?

#### R: Yes

No response required.

3)    Sequence generation (Item 8a)

#### Method used to generate random allocation sequence

#### Does the description make it clear if the "assigned intervention is determined by a chance process and cannot be predicted"?

#### R: Yes

No response required.

4)    Allocation concealment (Item 9)
Mechanism used to implement random allocation sequence (such as sequentially numbered containers), describing any steps taken to conceal the sequence until interventions were assigned

Is it clear how the care provider enrolling participants was made ignorant of the next assignment in the sequence (different from blinding)? Possible methods can rely on centralised or "third-party" assignment (i.e., use of a central telephone randomisation system, automated assignment system, sealed containers).

No response required.

5)    Blinding (Item 11a)

#### If done, who was blinded after assignment to interventions (for example, participants, care providers, those assessing outcomes)

#### Is it clear if (1) healthcare providers, (2) patients, and (3) outcome assessors are blinded to the intervention? General terms such as "double-blind" without further specifications should be avoided.

#### R: N/A

No response required.

6)    Outcomes and estimation (Item 17a/b)

#### For the primary outcome, results for each group, and the estimated effect size and its precision (such as 95% confidence intervals)

#### Is the estimated effect size and its precision (such as standard deviation or 95% confidence intervals) for each treatment arm reported? When the primary outcome is binary, both the relative effect (risk ratio, relative risk) or odds ratio) and the absolute effect (risk difference) should be reported with confidence intervals.

#### R: Done

No response required.

7)    Harms (Items 19)

#### All important harms or unintended effects in each group

#### Is the number of affected persons in each group, the severity grade (if relevant) and the absolute risk (e.g. frequency of incidence) reported? Are the number of serious, life threatening events and deaths reported? If no adverse event occurred this should be clearly stated.

#### Not included in current report. Authors should include some information on adverse events (if any) and how they were handled

See response above to Reviewer 2’s general comments.

8)    Registration (Item 23)

#### Registration number and name of trial registry

#### Is the registry and the registration number reported? If the trial was not registered, it should be explained why.

#### R: Yes; trial was registered with Clinitrials.gov NCT03177993 and ANZCTR N12617000738325).

No response required.

9)    Protocol (Item 24)

#### Where trial protocol can be accessed

#### Is it stated where the trial protocol can be assessed (e.g. published, supplementary file, repository, directly from author, confidential and therefore not available)?

#### R: Yes; attached as a supplementary file

No response required.

10)    Funding (Item 25)

#### Sources of funding and other support (such as supply of drugs) and role of funders

#### Are (1) the funding sources, and (2) the role of the funder(s) described?

#### R: Yes

No response required.

Reviewer #4: Scabies review

#### Accept

#### This is a well planned, executed, analysed and presented study.

#### However, I believe it would be worthwhile if the authors could make some points regarding the following issue:

#### 1. Is there any evidence that the nurses who conducted the clinical examinations were able to accurately diagnose scabies?  This can sometimes be difficult, even for dermatologists. Are the authors able to provide more information about their training?

In response to Reviewer 2 point 6 above, we have added additional details in the Methods regarding the training received and further elaboration in the Discussion regarding this as a limitation.

#### 2. Although not directly related to the study aims and findings, from the perspective of scabies control in general I think it would be helpful if the authors could (in the discussion) briefly mention that follow up incidence studies would be useful; for example in 2023 and 2028. This could yield information regarding the longevity of the effect. With this knowledge, an optimal frequency of mass treatments could be estimated.

We have added a sentence to the Discussion as follows, lines 421-423:

“In addition, these results are limited to the first 12 months after MDA. Previous studies have demonstrated that one round of MDA may have a prolonged and sustained benefit, but we will not have data to determine if this will be replicated at our study sites [6,8].”

#### 3. Briefly comment on the very low incidence rates in adults greater than ~35 years of age. Does this age group need to be mass treated? If cost is an issue, then halving the number of doses administered may permit mass treatments to be done more often: for example, every 5 years instead of every decade. (And it is certainly significant that the authors have been able to show that they can reduce costs by half by simply avoiding the second dose).

Adults are known to have a lower prevalence of scabies compared to children and the elderly in endemic populations. However, cases do exist among adults, and therefore we believe that adults be included in MDA initiatives to ensure all scabies transmission is reduced. The importance of treating adults is evident in reports of treatment failure when all household contacts do not receive treatment [2].

Reviewer #5: Statistical review

#### This paper reports a cluster randomised trial comparing different strategies for mass drug administration to reduce prevalence of scabies. The trial shows that the intervention arms were non-inferior to the control arm. The trial is generally reported well and the paper written clearly.

#### I have some comments, which I have provided below.

#### 1. Abstract, Line 60 and results: I would recommend providing a p-value for non-inferiority in addition to the 95% CI.

P-value added as suggested.

#### 2. Page 11 - how was stratification by island incorporated into the randomisation? Using stratified blocks? From this paragraph I would presume that villages agreed to participate prior to knowing the allocation and none dropped out after allocation - if this is not the case then please make this clear.

All villages agreed to participate prior to allocation and none dropped out after allocation. Stratification by island was implemented by randomising villages on an island equally to the treatment arms and separately for each island. To improve clarity we have added additional details to our method of randomisation as follows, lines 213-217:

“All 17 villages on Rotuma and 18 villages on Gau agreed to participate prior to treatment allocation. Randomisation of the 35 villages (clusters) was generated and allocated by an independent statistician using Stata software in a 1:1:1 ratio stratified by island. Stratification by island was implemented by randomising villages on an island equally to the treatment arms and separately for each island. No villages dropped out after allocation.”

#### 3. Page 13 - for the sample size calculation it appears the power was averaging over the different prevalence scenarios. I don't think enough detail on this is provided to allow reproduction of the sample size. Also, could the authors add what the assumed clustering coefficient (e.g. ICC) was? Lastly, Bonferroni correction was mentioned, but I did not see how this linked with using the 95% CI for non-inferiority (which implies no correction, unless the overall error rate is 5% one-sided).

The sample size derivations have been explained in more detail above (Reviewer 1 General Comments 1b, and we have added detail to the Supplementary Files). The 95% CI for non-inferiority referred to 95%-Bonferroni-corrected one-sided intervals, i.e. maintaining a global 5% Type I error rate. We did not specifically label these intervals as 98.3% confidence intervals, as the global Type I error is typically the primary matter of concern. We have added this detail as follows, lines 259-263:

“Utilising a Monte Carlo simulation tool with 1000 simulations and Bonferroni-adjusted one-sided confidence intervals (CI) that maintain a global type I error of 5%, we estimated 24 clusters of 100 with balanced random allocation of exposure, would be sufficient to achieve at least 80% power for comparison of treatment effect between any of the two groups.”

#### 4. Page 13-14: for the statistical analysis, two approaches are outlined - using the difference between follow-up and baseline prevalence, and then using a generalised linear approach.

#### a) For the former approach, could more details be provided on the model assumed. Presumably it is using summary data per cluster rather than individual patient data?

There is no model assumed. The summary is calculated per cluster, which is then summarised. This is mentioned above (Reviewer 1 General Comments 1c) and is described in the manuscript as follows, lines 274-277:

“To calculate the absolute reduction in prevalence between the two timepoints, the 12-month prevalence was subtracted from the baseline prevalence for every village, before calculating the mean of the difference for each treatment group.”

#### b) For the latter approach, is the baseline prevalence data used at all?

Baseline prevalence was not included. The generalised linear models would not run when baseline scabies prevalence included in the model.

#### 5.  I would imagine it's normal for this type of study, but the differential consent rates per arm are noticeable in table 1. It would be useful if the authors could discuss implications of this on how robust the results are. I suspect they are robust as it would require a huge difference between non-consenting and consenting participants to change the conclusions.

We agree that it would require a huge difference between consenting and non-consenting participants to alter the conclusions of the study. The proportion of the population consenting to participate ranged from 80-85.9% between treatment arms at baseline, and 78.5-84.8% at 12 months. In a community-based intervention such as our study, this difference would not be expected to significantly bias results.

Furthermore, we do not see any systematic sign of differential consent rate in our data. From Table S2, 51.9% of the general population in the IVM-2 arm were male and median age was 29 years, and among the participants who agreed to join the study, 51.9% were male and median age 25 years. In the IVM-1 arm, 51.1% of the population were male and median age 26 years, and among the participants who agreed to join the study, 50.7% were male and median age 22 years. in the SAT arm, 52.7% of the population were male and median age 28.5 years, and among the participants who agreed to join the study, 52.0% male were male and median age 27 years.

#### 6. Line 281 - many of the new cases were in participants newly in the study at 12 months - does this mean they were unlikely to have actually benefited from the interventions?

New participants at 12 months would not have received treatment as part of the study at baseline, however they would have indirectly benefited from a reduction in community prevalence and subsequent transmission of scabies.

#### 7. Line 297 - it's not clear to me what the significant p-value here means. Is this that it's significantly higher than 0? Can the estimated PAR and CI ever be negative?

We have removed the p-value.

#### 8. There are several secondary outcome measures mentioned on the clinicaltrials.gov registration page that are not reported in this paper. I would recommend that these are mentioned in the outcomes section and either reported here or a statement that they are to be reported separately provided.

As highlighted above in response to Reviewer 2, we have mentioned in the Introduction (lines 161-164) that this trial included safety and lymphatic filariasis efficacy outcomes as reported elsewhere. We have added further details in the Methods as follows, lines 287-291:

“The broader trial included evaluation of the safety of ivermectin, diethylcarbamazine and albendazole compared to diethylcarbamazine and albendazole, as well as the impact of these treatments on lymphatic filariasis at 12 and 24 months, which have been reported previously [13,14]. In addition, the effect on soil-transmitted helminths at 12 months and the acceptability of MDA in Fiji will be reported separately.”

#### 9. The protocol provided appears to be for the larger study rather than the scabies substudy specifically. If there is no specific protocol for the substudy, then pointing out the relevant sections of the protocol in the Supplementary text description may be useful.

We appreciate this suggestion and have added on the cover page of S1 Protocol a summary of the sections in the overall protocol relevant to scabies control as follows:

**“Sections relevant to scabies control include:**

2.1 Fiji specific background, pages 14-15

3.3 Potential participant benefits, pages 16-17

4.1 Study objectives, page 18

4.2 Study design, pages 18-19

4.9 Eligibility, pages 22-27

4.10 Pregnant and breastfeeding females, pages 27-28

4.13 Efficacy and effectiveness of IDA vs DA, page28

4.14 Retreatment, page 28

5.1 Study drug background, page 30

6.1 Triple drug therapy (IDA) and two-drug therapy (DA), page 32

6.3 Skin examination, pages 33-34

9 Statistical considerations, page42

Appendix 2 – Participant data collection form example, pages 60-61

Appendix 6 – Participant information statement and consent form, pages 82-85

Appendix 6A – Parent information statement and consent form, pages 88-91

Appendix 7 – Treatment acceptability study protocol, pages 103-104”

#### James Wason

# Additional corrections

We have removed the diethylcarbamazine abbreviation throughout the manuscript body.

Corrections to values in S2-S3 Tables in ‘Total all’ row, with additional formatting changes to S2-S7 Tables.

# References

1.	Wu S, Crespi CM, Wong WK. Comparison of methods for estimating the intraclass correlation coefficient for binary responses in cancer prevention cluster randomized trials. Contemp Clin Trials. 2012;33(5):869-80. Epub 2012/05/26. doi: 10.1016/j.cct.2012.05.004. PubMed PMID: 22627076; PubMed Central PMCID: PMCPMC3426610.

2.	La Vincente S, Kearns T, Connors C, Cameron S, Carapetis J, Andrews R. Community management of endemic scabies in remote aboriginal communities of northern Australia: low treatment uptake and high ongoing acquisition. PLoS Neglected Tropical Diseases [electronic resource]. 2009;3(5):e444. doi: <http://dx.doi.org/10.1371/journal.pntd.0000444>. PubMed PMID: 19478832; PubMed Central PMCID: PMCPMC2680947.
